# Estimating the economic effect of harm associated with high risk prescribing of oral non-steroidal anti-inflammatory drugs in England: population based cohort and economic modelling study

**DOI:** 10.1136/bmj-2023-077880

**Published:** 2024-07-24

**Authors:** Elizabeth M Camacho, Leonie S Penner, Amy Taylor, Bruce Guthrie, Anthony J Avery, Darren M Ashcroft, Daniel R Morales, Gabriel Rogers, Antony Chuter, Rachel A Elliott

**Affiliations:** 1 Manchester Centre for Health Economics, Division of Population Health, Health Service Research and Primary Care, University of Manchester, Manchester, UK; 2 Centre for Academic Primary Care, School of Medicine, University of Nottingham, Nottingham, UK; 3 NIHR Greater Manchester Patient Safety Research Collaboration, Manchester, UK; 4 Advanced Care Research Centre, Usher Institute, University of Edinburgh, Edinburgh, UK; 5 Centre for Academic Primary Care, School of Medicine, University of Nottingham, Nottingham, UK; 6 Centre for Pharmacoepidemiology and Drug Safety, School of Health Sciences, Faculty of Biology, Medicine and Health, University of Manchester, Manchester, UK; 7 Clinical Research Fellow, Population Health and Genomics, University of Dundee, DD1 4HN, UK; Department of Public Health, University of Southern Denmark, Odense, Denmark; 8 Patient author

## Abstract

**Objectives:**

To quantify prevalence, harms, and NHS costs in England of problematic oral non-steroidal anti-inflammatory drug (NSAID) prescribing in high risk groups.

**Design:**

Population based cohort and economic modelling study.

**Setting:**

Economic models estimating patient harm associated with NSAID specific hazardous prescribing events, and cost to the English NHS, over a 10 year period, were combined with trends of hazardous prescribing event to estimate national levels of patient harm and NHS costs.

**Participants:**

Eligible participants were prescribed oral NSAIDs and were in five high risk groups: older adults (≥65 years) with no gastroprotection; people who concurrently took oral anticoagulants; or those with heart failure, chronic kidney disease, or a history of peptic ulcer.

**Main outcome measures:**

Prevalence of hazardous prescribing events, by each event and overall, discounted quality adjusted life years (QALYs) lost, and cost to the NHS in England of managing harm.

**Results:**

QALY losses and cost increases were observed for each hazardous prescribing event (*v* no hazardous prescribing event). Mean QALYs per person were between 0.01 (95% credibility interval (CI) 0.01 to 0.02) lower with history of peptic ulcer, to 0.11 (0.04 to 0.19) lower with chronic kidney disease. Mean cost increases ranged from a non-statistically significant £14 (€17; $18) (95% CI −£71 to £98) in heart failure, to a statistically significant £1097 (£236 to £2542) in people concurrently taking anticoagulants. Prevalence of hazardous prescribing events per 1000 patients ranged from 0.11 in people who have had a peptic ulcer to 1.70 in older adults. Nationally, the most common hazardous prescribing event (older adults with no gastroprotection) resulted in 1929 (1416 to 2452) QALYs lost, costing £2.46m (£0.65m to £4.68m). The greatest impact was in people concurrently taking oral anticoagulants: 2143 (894 to 4073) QALYs lost, costing £25.41m (£5.25m to £60.01m). Over 10 years, total QALYs lost were estimated to be 6335 (4471 to 8658) and an NHS cost for England of £31.43m (£9.28m to £67.11m).

**Conclusions:**

NSAIDs continue to be a source of avoidable harm and healthcare cost in these five high risk populations, especially in inducing an acute event in people with chronic condition and people taking oral anticoagulants.

## Introduction

Non-steroidal anti-inflammatory drugs (NSAIDs) alleviate pain and inflammation by inhibiting isoenzymes of cyclooxygenase (known as COX-1 and COX-2). However, many adverse events are known to occur in people who use NSAIDs, particularly in relation to gastrointestinal bleeding, renal dysfunction, and cardiovascular function.^1^ ^2^ ^3^ NSAIDs, including aspirin, are responsible for 30% of hospital admissions related to an adverse drug event, mainly due to gastrointestinal bleeding, myocardial infarction, stroke, and renal injury.^4^ ^5^

Due to safety concerns for primary care, prescribing of oral NSAIDs in many countries has been reducing for the past 25 years. Total numbers of prescriptions have decreased and selection of potentially safer NSAIDs and prescribing of gastroprotective drugs have increased. Along with improved management of *Helicobacter pylori* infection, these changes have contributed to a 52% reduction in peptic ulcer incidence between 1997 and 2005,^6^ and reductions in NSAID prescribing in people with cardiovascular disease after regulatory warnings in 2004.^7^ ^8^ ^9^ In England, primary care prescribing of NSAIDs fell by about 12% between 2017 and 2022.^10^ Overall trends showed that, in 2022, naproxen accounted for 69% of prescriptions, prescribing was reduced for NSAIDs associated with higher cardiovascular risk, COX-2 inhibitor prescribing increased, and topical NSAID prescribing was sustained.^10^

NSAIDs have been the target of many prescribing safety initiatives including: increased emphasis on safer prescribing in general practitioner (GP) training and in GP targeted educational packages; supporting practices to identify patients with high risk prescribing; improvements to GP clinical record prescribing safety tools^11^ ^12^ ^13^; and community pharmacist prescribing quality schemes.^14^ However, NSAID prescribing is still common in people at high risk of adverse events due to older age, previous peptic ulcer, heart failure, chronic kidney disease, or prescription of other medications that can increase bleeding risk.^15^ ^16^ ^17^ These priority areas are being targeted by IT based, medical safety interventions, led by pharmacists, originally rolled out across the East Midlands region,^17^ followed by national roll-out in England.^18^ However, evaluation of these initiatives has largely been restricted to examining effect on prescribing rather than impact on population level outcomes. Identifying the most harmful and costly types of hazardous prescribing is likely to be important for policy makers, clinicians, and patients. For example, the main purpose of the Medicines Safety Improvement Programme in England is to address the most important causes of severe harm associated with medicines.^19^

The aims of this study were to quantify the prevalence in England of five types of high risk prescribing of oral NSAIDs, and to estimate the harm and NHS costs incurred by this prescribing to inform the targeting and evaluation of policy interventions.

## Methods

Previous work has identified five prescribing safety indicators as the most likely to be associated with avoidable harm from NSAIDs.^20^ ^21^ The five high risk areas are (1) prescription of an oral NSAID, without co-prescription of gastroprotection, to a patient aged 65 years or older; (2) prescription of an oral NSAID, without co-prescription of gastroprotection, to a patient with a history of peptic ulceration; (3) prescription of warfarin or directly-acting oral anticoagulant in combination with an oral NSAID; (4) prescription of an oral NSAID to a patient with heart failure; and (5) prescription of an oral NSAID to a patient with chronic kidney disease (estimated glomerular filtration rate <45 mL/min).

If a patient in one of these high risk areas was affected by potentially hazardous NSAID related prescribing, we have referred to this occurrence as a hazardous prescribing event (HPE). In this study, use of low dose aspirin was not included in the analysis.

We first obtained the prevalence of each HPE in the general population of England. We then estimated the harm associated with each of the five NSAID specific HPEs at the patient level, expressed as quality adjusted life years (QALYs) lost and the cost to the NHS in England of managing that harm. The combination of these two sets of data allowed the estimation of annual national levels of patient harm and NHS costs linked to NSAID prescribing in the five defined high risk groups.

### Prevalence of each HPE in the general population of England

The prevalence of each HPE in the general population of England was estimated from data reported from a nationwide study of prescribing safety in England carried out by this team.^18^ This study determined the number of patients affected by each of the five prescribing safety indicators (ie, numerator) and the number of patients in each of the five groups classified as being at risk of being affected (ie, denominator). The report for this study provides a baseline number of people who were affected by each HPE cross sectionally in April 2020, based on data from the national roll out of PINCER.^18^ The study included 2430 of 7131 general practices in England), from 130 clinical commissioning groups, and searches of over 23m patient records. The prevalence data came from the 1060 general practices (total patient population of 10 906 453) that provided data at two or more time points. We expect the sample to be representative of GP practices in England due to geographical spread of practices (across England) and size of the sample. The prevalence of each HPE was calculated by dividing the number of people with the HPE by the total patient population. The supplementary file (appendix 1) provides details of the practices that provided data for these prevalence estimates.

### Patient level harm and NHS cost of hazardous prescribing of NSAIDs

To estimate the patient level harm and costs associated with each HPE, a treatment pathway, or model, need to be designed to reflect the likely events that occur when people experience a HPE related to an NSAID. This model included what acute events happened, how frequently, and how serious was it for the patient. Additionally, details were needed as to how was short and long term quality of life, mortality risk, and health care resource consumption likely to be affected by this acute event. These models (also called cohort level state transition (Markov) models) were developed to generate estimates of patient outcomes (measured as QALYs) and cost to the NHS in England associated with each of the five high risk areas described above. QALYs are a measure of health status that combines quality of life and quantity of life into a single numerical value. Quality of life is measured as health utility on a 0-to-1 scale where 1 indicates perfect health and 0 indicates death. One QALY is equivalent to one year lived in perfect health, or two years lived in middling health (ie, a health utility of 0.5 over two years). In our model, the QALYs included reflect the negative health effects of adverse health events and do not include health benefits of taking NSAIDs. The supplementary file provides details of the overall methodological approach (appendix 2, section 1) and methods used to develop each of the models (appendix 2, sections 2 to 5).

#### Model design

The models were developed and reported according to standard validation and reporting criteria.^22^ ^23^ A health economic analysis plan was developed and is available from the authors. Replicable literature reviews were used to identify model inputs. Face validity was ascertained through continuous feedback from clinical and patient experts during the model build. Where possible, we used and adapted existing published models to optimise design.

Reflecting the prescribing indicator descriptions, we did not include topical NSAIDs because this method of application is considered relatively safe,^24^ or other parenterally administered NSAIDs because of the very low levels of prescribing.^25^ We did not treat COX-2 inhibitors separately from other NSAIDs because all NSAIDs can cause serious adverse effects, albeit with some variation in specific risks of gastro-intestinal and cardiac events, for example.^26^

This study estimates the harm associated with long term prescribing of oral NSAIDs in high risk groups by estimating the risk of the outcomes in the presence and absence of the HPE. In the HPE cohort, the probabilities of an adverse event are increased by the presence of the NSAID (in heart failure, co-prescription of oral anticoagulant, and chronic kidney disease), or by the use of the NSAID in the absence of gastroprotection (in older people or those with previous peptic ulcer). The cohort in the non-HPE group was assumed to have had the following treatments: older people, or those with previous peptic ulcer were assumed to have had NSAIDs plus gastroprotection; people taking oral anticoagulant, or those with heart failure or chronic kidney disease were assumed to have taken paracetamol. These assumptions were validated with our patient and clinical experts.

Data were required for each of the five economic models. We collected cohort baseline characteristics. All data categories reflected real-life patient cohort characteristics (age, sex, and relevant diagnosis) as closely as possible, by selecting data sources that either were from relevant UK cohorts, or from cohorts that closely resembled UK real life patients. We collected data for health states. Each model included health states for key adverse events associated with the HPE and death. Relevant adverse events were identified from published literature and verified through discussion with experts. Data for probabilities of harm associated with HPEs and subsequent events after harm has occurred were collected. The absolute risk of harm (adverse events), associated with HPEs was derived from large population based observational studies, wherever possible. Health status was collected and expressed as utility, associated with a particular health state, where 1.0 is equivalent to perfect health and 0 is equivalent to being dead. We used published estimates of health status to attach a utility value to each health state in the Markov models. Resources consumed in a particular health state were noted. Where available, data from large population based observational datasets were used to estimate resource use associated with harm. If these were not available, we used expert opinion to estimate resource use. We attached UK reference unit costs to resource use to construct a total cost for each health state (eg, NHS costs post-gastric bleed). Table 1 summarises the health states included in each of the models. The probability, utility, and cost parameters, and their sources, are summarised for each model in tables 6.1 and 6.2 in the appendix.

**Table 1:** Summary of health states included in each model

High risk group	Adverse events that are included in each model
Non-steroidal anti-inflammatory drug (NSAID) in older people without gastroprotection	Gastrointestinal discomfort, symptomatic ulcer, serious gastrointestinal event (eg, a bleed), death
NSAID with previous peptic ulcer with no gastroprotection	Gastrointestinal discomfort, symptomatic ulcer, serious gastrointestinal event (eg, a bleed), death
NSAID with oral anticoagulant	Gastrointestinal discomfort, symptomatic ulcer, serious gastrointestinal event (eg, a bleed), stroke, death
NSAID with heart failure	Minor heart failure exacerbation, major heart failure exacerbation requiring hospital admission, death
NSAID with chronic kidney disease	Acute kidney injury episode managed in primary care, acute kidney injury episode requiring hospital admission with or without renal replacement therapy, residual effect on kidney function, and progression of chronic kidney disease, death

#### Analysis

We determined the impact by estimating the effect of HPE on a patient via the impact on QALYs and costs. The follow-up period was chosen to be sufficient to capture relevant costs and effects without exacerbating uncertainty owing to excessive extrapolation. We set the period at 10 years in the primary (base case) analysis, after consultation with our clinical experts who suggested that this time was sufficient to encompass events of plausible interest. As such, our base case models estimate QALYs and costs over a 10 year period following the onset of the HPE. We tested this decision by varying the time horizon between five and 20 years in sensitivity analysis. We took the perspective of the NHS: we only included the costs incurred by the NHS of managing the consequences of the HPE. People value present costs and benefits more than future costs and benefits, so studies with a time horizon over one year needed to discount future costs and benefits. The QALYs and costs were discounted at the recommended rate of 3.5% per annum, and the resulting effect was examined in a sensitivity analysis.^27^ The cost year used was 2020-21 (currency: UK £). Models were built in Microsoft Excel. Each model was populated with probability, cost and health status data, to allow for the generation of the point estimates and uncertainty in distributions of discounted outcomes (QALYs) and NHS costs in a cohort affected by a specific type of HPE, and a cohort not affected. We assumed that the association between exposure time and risk of adverse event was roughly linear, supported to an extent by the literature for gastrointestinal, renal, and cardiovascular outcomes.^28^ ^29^ For the probabilistic analysis, distributions appropriate for input parameters were chosen.^30^ If no measure of uncertainty was available for the β or gamma distribution, we assumed that the standard deviation defining the distribution was 20% of the mean.^30^ The probabilistic analysis was based on 10 000 samples. We made an assumption in the absence of empirical evidence around usual length of exposure to the HPE without an event precipitating review. Length of exposure to the HPE was assumed to be the lifetime of the model (10 years), unless the patient had an adverse event when it was assumed that the hazardous prescribing was stopped.

Sensitivity analysis was used to examine the effects of varying the following parameters: time horizon of five years and 20 years; no discount rate; duration of HPE exposure varied from 0.25 to 10 years. Our primary analysis assumes effects are additive (ie, people who have had multiple HPEs may experience multiple harms). Sensitivity analysis was carried out to minimise the overlap by deducting the number of people at risk for anticoagulants, heart failure, and chronic kidney disease from the group of people older than 65 who are at gastrointestinal risk so that people aged over 65 will only be at risk of gastrointestinal harm if they are not part of the oral anticoagulant, heart failure, or kidney disease cohorts. Further sensitivity analysis examined the effect of assuming NSAIDs in use are a weighted average of celecoxib, diclofenac sodium, ibuprofen, and naproxen (compared with the base case assumption of 100% naproxen).

### Scaling up prevalence, harm, and cost

To estimate the number of people with each HPE across England, the prevalence was multiplied by the number of people registered with a GP practice in England on 1 December 2023 (n=63 049 603).^31^ Finally, the number of people estimated to be affected by each HPE was multiplied by the respective per person QALY loss and cost to estimate the overall burden of each HPE in England. The QALY losses and costs were added together across all five HPEs to estimate an approximate combined burden.

### Patient and public involvement and engagement

Patients and clinicians were involved in all stages of this work including AC, who is a co-author. Extensive consultation was undertaken during the development of the economic models, with patients, GPs, and pharmacists consulted at multiple design stages. Their input influenced model structure and treatment pathways, as well as how to report findings.

## Results

The cost and harm associated with each HPE is summarised in table 2. A net QALY loss was observed for each HPE when comparing the mean QALYs generated per person in the presence and absence of the HPE. The QALY losses per person ranged from 0.01 (95% credibility intervals (CI) 0.01 to 0.02) in those with a previous peptic ulcer, to 0.11 (0.04 to 0.19) in people with chronic kidney disease. The difference in the mean costs to the NHS in the presence and absence of the HPE ranged from non-statistically significant increases (£14 (€17; $18) (−£71 to £98) per person with heart failure) to statistically significant increases (£1097 (£236 to £2542) per person concurrently taking oral anticoagulants.

**Table 2:** Summary of key cost and outcome parameters derived from each hazardous prescribing event specific model

Type of hazardous prescribing event	Mean costs generated per patient (£) (95% CI)				Mean QALYs generated per patient (95% CI)		
	Hazardous prescribing event	No hazardous prescribing event	Difference (event minus no event)		Hazardous prescribing event	No hazardous prescribing event	Difference (event minus no event)
NSAID in older people with no gastroprotection	258 (195 to 339)	234 (174 to 312)	23 (6 to 44)		5.836 (5.762 to 5.910)	5.853 (5.777 to 5.927)	−0.017 (−0.021 to −0.012)
NSAID with previous peptic ulcer with no gastroprotection	277 (209 to 363)	253 (187 to 337)	24 (6 to 45)		6.700 (6.630 to 6.769)	6.715 (6.643 to 6.785)	−0.014 (−0.019 to −0.010)
NSAID with oral anticoagulant	2526 (1372 to 4260)	1429 (935 to 1990)	1097 (236 to 2542)		4.812 (4.598 to 5.022)	4.904 (4.703 to 5.106)	−0.093 (−0.176 to −0.039)
NSAID with heart failure	12 607 (8378 to 17 755)	12 594 (8374 to 17 723)	14 (−71 to 98)		2.998 (2.784 to 3.202)	3.045 (2.829 to 3.252)	−0.046 (−0.071 to −0.026)
NSAID with chronic kidney disease	50 215 (48 342 to 52 185)	50 008 (48 260 to 51 803)	207 (−163 to 703)		4.523 (3.936 to 5.051)	4.634 (4.024 to 5.177)	−0.111 (−0.194 to −0.039)

Cost year 2020-21.

CI=credible intervals; NSAID=non-steroidal anti-inflammatory drug; QALY=quality adjusted life year.

HPE prevalence is summarised in table 3. HPE prevalence per 1000 patients ranged from 0.11 in people with a previous peptic ulcer who were given NSAIDs (and no gastroprotective drugs) to 1.70 in older adults given NSAIDs (and no gastroprotective drugs).

**Table 3:** Prevalence of hazardous prescribing events (HPEs), total cost, and QALY loss for England over 10 years

Outcome		NSAID in older people without gastroprotection		NSAID with previous peptic ulcer		NSAID with oral anticoagulant			NSAID with heart failure		NSAID with chronic kidney disease	Total	
**Sample of GP practices in England (n=10 906 453)** 18													
No of people at risk		1 355 707		83 104		245 778			87 804		136 749	1 909 142	
No of people at risk of HPE per 1000 people (95% CI)		124.3 (124.1 to 124.5)		7.6 (7.6 to 7.7)		22.5 (22.4 to 22.6)			8.1 (8.0 to 8.1)		12.5 (12.5 to 12.6)	N/A	
No of people affected by an HPE		18 591		1188		4005			1544		2733	28 061	
No of people affected by an HPE per 1000 people (95% CI)		1.70 (1.68 to 1.73)		0.11 (0.10 to 0.12)		0.37 (0.36 to 0.38)			0.14 (0.13 to 0.15)		0.25 (0.24 to 0.26)	N/A	
**English population registered with a GP (n=63 049 603)**													
Estimated no of people affected by an HPE (95% CI)		107 474 (105 935 to 109 023)		6868 (6483 to 7264)		23 153 (22 441 to 23 875)			8926 (8486 to 9376)		15 799 (15 213 to 16 397)	162 219 (160 329 to 164 120)	
**Base case**													
Total cost impact (£, millions*) (95% CI)		2.46 (0.65 to 4.68)		0.16 (0.04 to 0.31)		25.41 (5.25 to 60.01)			0.13 (−0.60 to 0.89)		3.27 (−2.41 to 11.24)	31.43 (9.28 to 67.11)	
Total QALY impact (95% CI)		−1929 (−2452 to −1416)		−114 (−148 to −81)		−2143 (−4073 to −894)			−411 (−628 to −232)		−1738 (−3043 to −640)	−6335 (−8658 to −4471)	
**Sensitivity analyses**													
Five year period:													
Total cost impact (£, millions*) (95% CI)		2.39 (0.59 to 4.57)		0.16 (0.04 to 0.30)		16.12 (3.59 to 35.48)			0.59 (0.05 to 1.32)		4.36 (0.26 to 10.02)	23.62 (9.70 to 44.04)	
Total QALY impact (95% CI)		−1597 (−2108 to −1128)		−98 (−131 to −67)		−832 (−1536 to −361)			−304 (−453 to −177)		−688 (−1278 to −235)	−3518 (−4532 to −2587)	
20 year period:													
Total cost impact (£, millions*) (95% CI)		2.44 (0.64 to 4.71)		0.16 (0.04 to 0.30)		31.11 (5.76 to 71.49)			−0.07 (−0.91 to 0.70)		−5.11 (−12.88 to 1.97)	28.53 (1.87 to 71.93)	
Total QALY impact (95% CI)		−2273 (−2843 to −1721)		−129 (−164 to −94)		−3560 (−6658 to −1558)			−449 (−681 to −258)		−2587 (−4356 to −1003)	−8998 (−12 554 to −6390)	
0% discount rate:													
Total cost impact (£, millions*) (95% CI)		2.57 (0.76 to 4.84)		0.17 (0.05 to 0.31)		28.97 (5.55 to 65.48)			−0.09 (−0.92 to 0.70)		3.20 (−3.46 to 11.85)	34.82 (9.37 to 71.63)	
Total QALY impact (95% CI)		−2095 (−2664 to−1553)		−123 (−159 to −89)		−2571 (−5076 to −973)			−473 (−727 to −278)		−2160 (−3810 to −772)	−7423 (−10 291 to −5169)	
Minimising overlap of populations at risk:													
Total cost impact (£, millions*) (95% CI)		1.37 (0.28 to 2.62)		0.16 (0.03 to 0.31)		24.79 (5.12 to 59.15)			0.14 (−0.56 to 0.88)		3.31 (−2.35 to 10.97)	29.78 (8.13 to 64.25)	
Total QALY impact (95% CI)		−1065 (−1359 to −782)		−114 (−150 to −81)		−2094 (−4026 to −868)			−408 (−616 to −233)		−1739 (−3120 to −599)	−5420 (−7658 to −3678)	
NSAIDs as a weighted average compared with base case†:													
Total cost impact (£, millions*) (95% CI)		2.45 (0.59 to 4.67)		0.16 (0.04 to 0.31)		27.02 (7.20 to 60.22)			−0.25 (−0.84 to 0.25)		1.31 (−4.50 to 9.45)	30.68 (9.11 to 64.75)	
Total QALY impact (95% CI)		−1930 (−2464 to −1417)		−114 (−150 to −81)		−2145 (−4030 to −893)			−519 (−708 to −359)		−1756 (−3079 to −642)	−6464 (−8786 to −4590)	

CI=credible intervals; GP=general practitioner; NSAID=non-steroidal anti-inflammatory drug; QALY=quality adjusted life year

* Cost year 2020-21.

† Weighted average of celecoxib, diclofenac sodium, ibuprofen, and naproxen compared with base rate of 100% naproxen.

**Fig 1:**
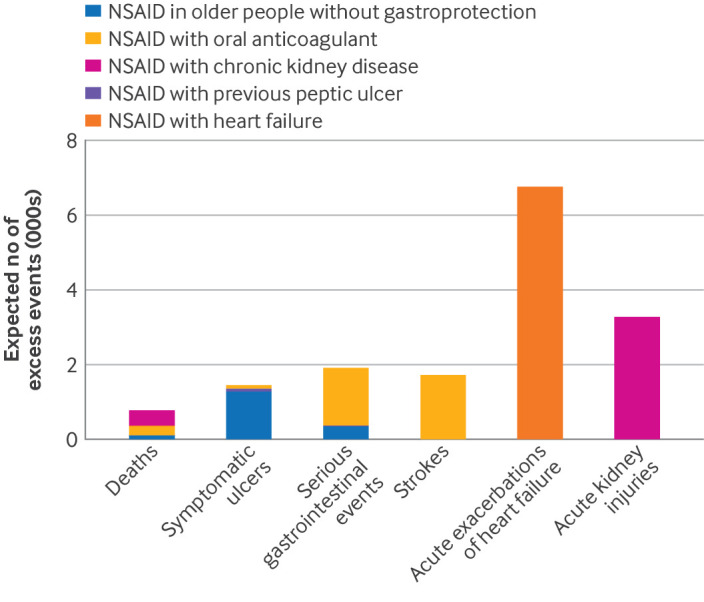
Expected number of excess events over 10 years in the English population. NSAID=non-steroidal anti-inflammatory drug

Scaling up the HPE prevalence from the sample of 10 906 453 to the English population registered with a GP (n=63 049 603) produced an estimate of 162 219 HPEs associated with NSAIDs in one year in England. Of these events, 66.3% were prescribing NSAIDs to older adults with no gastroprotective drug (table 3). We estimated that these HPEs will be associated with 778 excess deaths in England in the subsequent 10 years, as well as substantial gastrointestinal, neurological, cardiac, and renal morbidity (fig 1). In particular, the analyses highlighted NSAIDs’ propensity to induce an acute event in people with a chronic condition (>6700 heart failure exacerbations and >3200 acute kidney injuries over 10 years).

The estimated total cost and QALY loss over 10 years following the onset of each of these HPE related to NSAIDs is estimated for the population of England (table 3). The most common HPE (NSAIDs in older adults with no gastroprotective drug) resulted in 1929 (95% CI 1416 to 2425) QALYs lost, costing £2.46m (95% CI £0.65m to £4.68m). The HPE associated with greatest harm and cost nationally was in people taking oral anticoagulants, with 2143 (894 to 4073) QALYs lost, costing £25.41m (£5.25m to £60.01m). The estimated total QALY loss across all the HPEs was 6335 (4471 to 8658) and an NHS cost of £31.43m (£9.28m to £67.11m).

**Fig 2:**
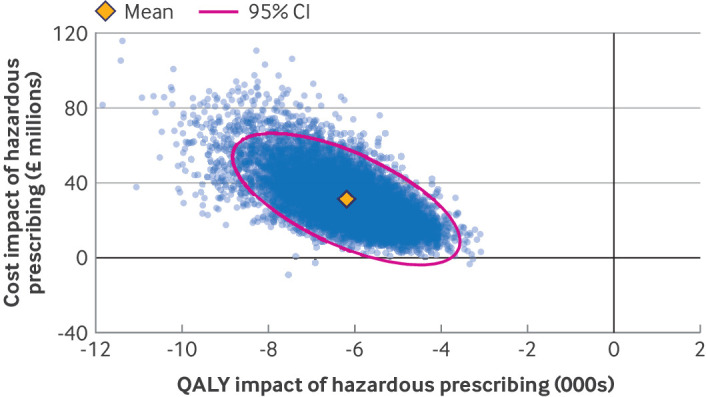
Total cost and quality adjusted life year (QALY) loss for England over 10 years associated with hazardous prescribing events related to non-steroidal anti-inflammatory drug (NSAID). Mean incremental QALY loss and incremental cost increase is presented with the simulated distribution from 10 000 simulations, presented as a data cloud. The pink dotted ellipse represents the boundary within which 95% of estimated incremental QALY loss and incremental cost increase is expected to occur

Figure 2 shows the degree of uncertainty around this estimate. Across 10 000 simulations drawing from the full distribution of uncertain parameters, 99.94% suggested that hazardous prescribing related to NSAIDs is associated with additional costs to the NHS and all showed that these prescribing events had a negative impact on patient health.

**Fig 3:**
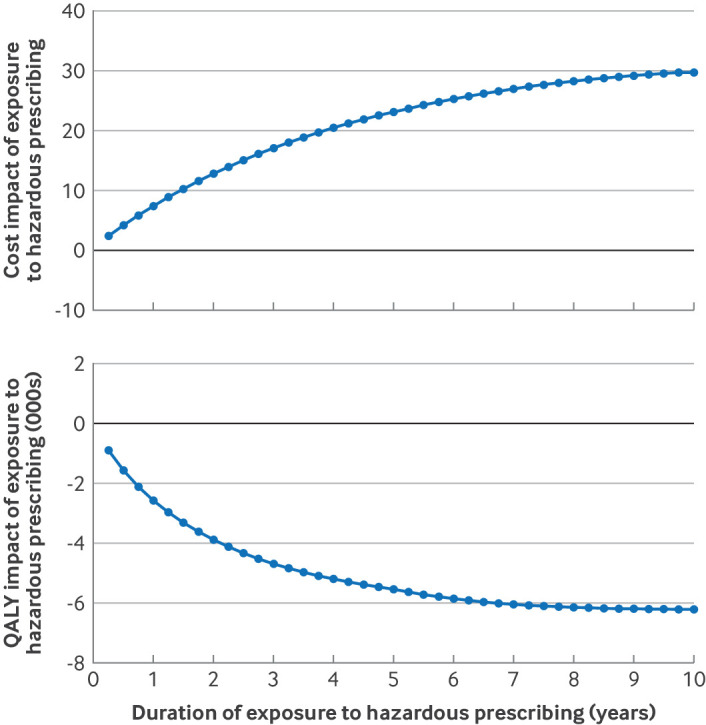
Impact of assumptions around length of exposure to the hazardous prescribing event

When we varied each model parameter over the range of its plausible values, none was influential enough in isolation to overturn the cost and QALY impacts associated with NSAID related HPEs (figure S2, appendix 3). The inputs with the biggest effect on costs were those relating to stroke, an outcome that only featured in our analysis of NSAIDs in people taking oral anticoagulants. The parameters that affected QALYs most related to stroke and acute kidney injury. However, the parameter that made the biggest difference in both dimensions was the length of time for which we assumed people remained affected by the HPE. Further exploration of this parameter showed that NSAIDs were associated with harm and cost even if exposure was brief, and that at least half of the harm was likely to be experienced within the first one and a half years (fig 3).

When we shortened the models’ time horizons, the effect of QALY losses and cost associated with NSAID related hazardous prescribing reduced. The opposite was mostly the case when we lengthen the time horizon or reduce the discount rate. Counterintuitively, however, expected excess costs were slightly lower with a 20 year time horizon than in the base case. This effect was due to the kidney disease model, where people affected by an HPE have a higher chance of premature death whereas, in the absence of the HPE, a greater proportion of people live long enough to consume expensive resources as their disease progresses. In a final sensitivity analysis, we explored the potential for double counting in our analysis by deducting 100% of people in the oral anticoagulant, heart failure, and kidney disease cohorts from the group of people aged over 65 who are at risk of gastrointestinal harm. The data showed that the results were not meaningfully affected by our base case assumption of additive effects, with cost and QALY reducing by 5-10%.

## Discussion

### Principal findings

The most commonly occurring HPE is of NSAIDs in older people without gastroprotective drugs (prevalence 1.70/1000 patients), which was associated with an estimated total loss of 1929 QALYs and a cost of £2.46 m. However, although NSAID with oral anticoagulant was much less common (0.37/1000 patients), it was associated with larger QALY loss (2143) and much larger costs (£25.41m). The five NSAID related HPEs caused a loss of 6335 QALYs at a cost to the NHS of £31.43m in England over 10 years. Reducing the length of time the patient was assumed to be affected by the HPE substantially reduced estimates of harm and cost, but at least half the estimated harm in the full model accrued within the first one and a half years of treatment.

### Comparisons with other literature

Most studies worldwide have estimated the harm and costs associated with NSAIDs and focused on the short term costs of admission to hospital for gastrointestinal bleeds related to NSAIDs, sometimes including dyspepsia costs.^32^ ^33^ ^34^ ^35^ ^36^ ^37^ ^38^ Our modelling approach incorporated a more complete estimate of gastrointestinal effects over a longer time period, including that associated with rebleeding, and includes more comprehensive primary care costs, which increased the estimates of harm and cost.^39^ The effect of NSAIDs on outcomes beyond gastrointestinal outcomes, such as renal and cardiovascular outcomes, has been less widely investigated. A review reported effects on these three types of outcomes but did not estimate costs.^29^ Two studies incorporated NSAID related cardiovascular risk into their model when estimating the harm and costs associated with NSAIDs.^40^ ^41^ While both studies used approaches similar to ours, they did not explicitly report the harms and costs associated with cardiovascular risk related to NSAIDs. Interest in the burden associated with renal harms of NSAIDs has been increasing in recent years: a 2021 Japanese study estimated the economic burden of renal events associated with NSAID use in patients with chronic kidney disease.^32^ This study only calculated one year costs and did not assess the effect of acute kidney injury on risk of chronic kidney disease progression. As such, their results are difficult to compare directly with our 10 year results; however, even in one year, they suggest that costs in Japan can reach $1779 for hospital admissions and $33 018 for dialysis per person.

### Strengths and limitations

Key strengths of the study are the estimation of the harm and NHS costs incurred by NSAID prescribing across range of common gastrointestinal, cardiovascular, and renal harms possible in key groups at high risk for adverse events. These factors included longer term harms and costs, such as those associated with gastrointestinal rebleeding, and acute kidney injury associated increases in the risk of chronic kidney disease progression. Combining these model estimates with real-world data on HPE rates provides a more complete picture of the scale of harm associated with NSAIDs than previous analyses.

The study has several limitations, around assumed dose, length of exposure, accounting for all harms, and assumptions around independence of multiple HPEs, although we explicitly explored many of these limitations in sensitivity analysis. Firstly, we did not have access to data about the length of time people are affected by specific HPEs. Recent work in the UK suggests that more than 60% of patients in England who have an NSAID prescribed for regular use have it prescribed for more than two months.^42^ Based on advice from clinicians, our models assumed exposure for the length of the model (10 years), unless an adverse event alerts the prescriber to take corrective action. A sensitivity analysis for shorter durations of exposure found (as expected) that shorter durations of exposure were associated with lower harms, but that at least half of observed harm accrued in the first one and a half years. In addition, NSAID exposure often varies as underlying pain increases or decreases, but the model assumes constant dosage. However, the source data for risk of adverse events comes from routine data where exposure will have similar variation, so we do not expect this to cause bias. The model is also constrained to a 10 year time horizon, which might underestimate costs and harms from HPEs, although sensitivity analysis using a 20 year time horizon does not alter the core conclusions. As naproxen was the most prescribed NSAID in England, the models assume the NSAID is standard dose naproxen. Therefore. not accounting for variation in drug or dosage is a limitation. However, data for the effect of drug or dose on all the adverse events under investigation were sparse.

Secondly, the models assume that harms are additive but independent, but a sensitivity analysis testing this assumption found only small reductions in estimated cost and QALY impacts. Our previous work on the incidence of these HPEs suggested that very few patients had more than one type of HPE.^17^

Thirdly, not all harms are accounted for which underestimates harm and cost. We have focused on selected high risk groups prioritised for the PINCER roll-out but not all NSAID harms were captured. For example, we did not include the harm of acute kidney injury that might occur in people with normal renal function^43^ or cardiac outcomes in people with ischaemic heart disease taking NSAIDs.^44^ The prescribing data used in our study did not capture over-the-counter use of NSAIDs, although over-the-counter doses tended to be lower than prescribed doses, with a lower risk of adverse drug events.^45^ ^46^

The use of UK prescribing data, patient management pathways, and unit costs means that providers and policy makers from other settings will need to interpret our findings with caution before extrapolating to their settings. However, the types, severity, and probability of harm related to NSAIDs are likely to be transferable, and can be combined with local prescribing data, patient management pathways, and unit costs.

### What this work adds, and implications for prescribers and policy makers

NSAIDs continue to be an important source of avoidable harm and healthcare costs, despite a range of regulatory and prescribing quality initiatives to reduce their use in high risk populations.^47^ ^9^ ^11^ ^17^ ^41^ The key implication for policy and practice is that despite quite large improvements in high risk prescribing of NSAIDs in the past 10-15 years, more work needs to be done. In practice, reducing NSAID prescribing on an individual level by prescribers is challenging because it depends on how much pain a patient is in, how well they respond to NSAIDs, and how well they respond to other analgesics or interventions. We have focused on use of NSAIDs in high risk groups, rather than all NSAID prescribing because reducing NSAID prescribing in low risk groups should not be a policy priority. Our findings are designed to be relevant to current policy initiatives in England, focusing on problematic polypharmacy (ie, taking multiple drugs concurrently) as one of the main consequences of overprescribing.^48^ This study provides a baseline estimate of harm and costs, and the models provide a framework to support robust evaluation of interventions to reduce high risk prescribing of NSAIDs.

Further research should extend this work: to other kinds of NSAID risk; to better understand exposure over time and account for it; and to optimise effectiveness of NSAID alternatives perhaps, which might be pharmacological or non-pharmacological. We also suggest that increasing patient awareness of the risks of NSAIDs needs to be considered as part of interventions to reduce high risk prescribing of NSAIDs. Other areas targeted by national policy might also benefit from the same explicit approach to inform choice of focus.

### Conclusions

NSAIDs continue to be a source of avoidable harm and healthcare costs, despite a range of initiatives to reduce their use, especially in populations at high risk. The risk of harm and associated costs appear to outweigh their benefit in these populations; therefore, a concerted effort should be made to continue to include NSAIDs in patient safety and deprescribing initiatives.

**What is known on this topic**

Non-steroidal anti-inflammatory drugs (NSAIDs) are one of the most widely prescribed groups of medicines worldwide, although prescribing rates are reducing
NSAIDs can cause patient harm, such as gastrointestinal bleeding, myocardial infarction, stroke, and renal damage

**What this study adds**

Problematic NSAID prescribing in high risk groups is prevalent, with 107 000 people older than 65 years being prescribed NSAIDs without gastroprotection, annually
NSAIDS can cause most harm when prescribed in people who are also taking oral anticoagulants
Problematic NSAID prescribing costs NHS England an estimated £31m (€37m; $39m) over 10 years, with a loss of about 6300 quality adjusted life years

## Data Availability

All data and the economic model code are available in the manuscript and supplementary materials.

## Web extra

Extra material supplied by authors

Web appendix: Harm calculations of non-steroidal anti-inflammatory drugs

Web appendix: Extra material supplied by authors

## Notes

Contributors: All authors contributed to the study concept and design, and the acquisition, analysis, or interpretation of data. Economic models were developed and reviewed by EC, GR, RAE, LP, BG, DM, AJA, AC, and DMA. RAE wrote the first draft of the manuscript. EC, GR, and AT conducted the analyses. All authors critically revised the manuscript for important intellectual content and approved the final version. AJA obtained the funding. The corresponding author (RAE) attests that all listed authors meet authorship criteria and that no others meeting the criteria have been omitted. EC and RAE have full access to all the data and all authors have full access to the statistical reports and tables in the study. EC and RAE can take responsibility for the integrity of the data and the accuracy of the data analysis.

Transparency: The lead author (EC, manuscript guarantor) affirms that the manuscript is an honest, accurate, and transparent account of the study being reported; that no important aspects of the study have been omitted; and that any discrepancies from the study as planned (and, if relevant, registered) have been explained.

Dissemination to participants and related patient and public communities: The results of the study will be sent to relevant patient and public communities, partnering with our Patient Safety Research Collaboration to target patient, professional and policy audiences, using Facebook, Instagram, and X accounts, plus newsletters.

Provenance and peer review: Not commissioned; externally peer reviewed.
