# Supplementary material for: Estimating the economic effect of harm associated with high risk prescribing of oral non-steroidal anti-inflammatory drugs in England: population based cohort and economic modelling study
Source: BMJ. 2024 Jul 24;386:e077880. doi: 10.1136/bmj-2023-077880 (PMC11268384; doi:10.1136/bmj-2023-077880)
Supplement: Supplementary file 2 — Web appendix: Extra material supplied by authors [file came077880.ww2.pdf]

Appendix 1: Characteristics of general practices providing hazardous prescribing events data

Table 1.1 Reproduction of data from 'PINCER National Rollout Progress Report to NHS England and the AHSN Network Extended Executive Summary' (1) - Summary characteristics of general practices providing data used to estimate the prevalence of each HPE in the general population of England

AHSN	Number of CCGs involved in uploading data (n)	Number of practices involved in uploading data (n)	Total practice population (n)	Number of practices who uploaded at least two sets of data (n)*
East Midlands	15	204	1,833,712	109
Eastern	11	116	1,495,135	50
Health Innovation Manchester	1	4	27,109	0
Health Innovation Network	11	211	2,111,582	78
Imperial College Health Partners	8	202	1,496,066	45
Innovation Agency	8	153	1,210,980	37
Kent Surrey Sussex	14	119	1,247,611	43
Oxford	4	204	2,266,584	133
South West	3	99	1,033,696	54
UCL Partners	12	239	2,123,566	83
Wessex	9	234	2,652,721	202
West Midlands	14	252	2,068,728	16
West of England	4	176	1,873,604	153
Yorkshire & Humber	16	217	1,909,602	57
Total	130	2,430	23,350,696	1060

*Baseline total practice population = 10,906,453; values in the table are reproduced from Appendix 4 and 5 of the PINCER Progress Report (1)

CCG: Clinical commissioning group; n: number

Appendix 2: Derivation of models to determine harm from Non-steroidal anti-inflammatory drugs (NSAID)-related hazardous prescribing events

1. GENERAL METHODOLOGICAL APPROACH TO BUILDING DISEASE-SPECIFIC MODELS

To estimate the patient-level harm and costs associated with each hazardous prescribing event (HPE), it was necessary to design a treatment pathway, or "model" that reflected the likely events that occur when people experience an NSAID-related HPE. This included what acute events happen, how commonly, and how serious is it for the patient; and also included how is short and long-term quality of life, mortality risk and health care resource consumption likely to be affected by this acute event. These models (also called cohort-level state transition (Markov) models) were developed to generate estimates of patient outcomes (measured as quality-adjusted life-years (QALYs)) and cost to the NHS in England and patient outcomes (measured as QALYs) associated with the five NSAID-specific HPEs.

The economic models needed to reflect the likely events that occur when people experience an NSAID-related HPE:

- What happens and how serious is it for the patient?
- Is this likely to result in primary or secondary care management?
- Is this event likely to increase risk of mortality or reduce quality of life in the short-term?
- How is long-term quality of life, mortality risk and health care resource consumption likely to be affected by this acute event?

States included were specific to the HPE under investigation. The length of time (also called sojourn time) a person spends in one health state is referred to as "cycle length". A cycle length of three months was used as this reflected the natural potential rate of change from one state to another. A half-cycle correction was used to account for the timing of events within a health state sojourn time. Each model started with an "event-free" health state. There are assumed to be no costs associated with the "event-free" health state. Each model contained an absorbing health state ("dead"). We determined the impact by estimating the effect on costs and QALYs using a 10-year time horizon. We took the perspective of the NHS in terms of direct costs of managing the consequences of hazardous prescribing. Total QALYs were calculated as the time in each state weighted by state-specific utility score. The costs and outcomes were discounted at the recommended rate of 3.5% per annum (2). The cost year used was 2020/21. Where it was necessary to inflate costs, the Hospital & Community Health Services (HCHS) index was used to inflate costs up to 2014/15 and then the newer NHS Cost Inflation Index (NHSCII) was used to inflate from 2014/15 to 2020/21.(3) The models were developed and reported according to standard validation and reporting criteria (4, 5). Validation was carried out by a team member not involved in the model build. Face validity was ascertained through continuous feedback from clinical and patient experts during the model build.

1.1. SOURCES OF DATA AND MODEL DESIGN

The development of each model is described below. Where possible we utilised and adapted existing published models to optimise design. A literature search for each model was conducted through Medline (1946-01/2020), Embase (1974-01/2020), HTA database (01/01/2010 to 31/03/2018 when database was last updated), NHS EED (01/01/2010 to 2015 when database was last updated) and Web of Science (01/2000-01/2020) using treatment pathway-specific search terms. If a relevant systematic review had been carried out, the search was updated and this is detailed in the specific search strategy. Inclusion criteria for studies could

not be too restrictive due to the range of types of parameters required, and the lack of data available for some parameters. Inclusion criteria are listed below:

- Publication language – English
- Publication date – any, although preference given to newer studies
- Country – any, although preference given to UK-based studies
- Population – reflective of patient group in the model, otherwise general population samples of adults including male and female participants
- Publication type – papers published in peer-reviewed journals preferable, but grey literature included if necessary
- Study design – any relevant to parameter of interest, although preference given to large sample sizes
- Setting – any healthcare or community setting, preference given to setting relevant to HPE
- Systematic reviews – included and preferable to single-study sources
- Animal studies - Human studies only

We used standard hierarchies of evidence to ensure the best quality data were used. Quality of data used was explicitly discussed in each model. After excluding duplicate records, studies were included if they examined issues on the incidence, prevalence, treatment or resource use of the consequences of each HPE. Reference lists of the retrieved references were hand-searched. The search strategy for each model is summarised in the model-specific sections below.

Data required for the economic models were probabilities of harm associated with an HPE, subsequent events after harm has occurred, the health status associated with a particular health state (e.g. health status post-gastric bleed), and the resources consumed in a particular health state (e.g. NHS costs post-gastric bleed). These data came preferentially from up-to-date UK sources that reflected the characteristics of the patient populations at risk of hazardous prescribing. If possible, individual patient data were used, with associated measures of mean and variation. If these were not available, point estimates were used, with carefully specified deterministic ranges, using standard methods for allocating distributions to these data. If necessary, expert opinion or explicitly stated assumptions were used to generate some parameters, which were discussed with the project team clinical and lay experts, and if necessary, the effect of these assumptions was tested in sensitivity analysis.

We used the same data sources between models where appropriate, where model design and parameters needed meant that we could do this. This information is provided in detail in the summary of each model.

1.2 COHORT BASELINE CHARACTERISTICS

All data categories reflected real-life patient cohort characteristics (age, sex, relevant diagnosis) as closely as available. Where possible, UK-based HPE studies and the large population-based observational studies used to link harm to HPEs were used to define initial cohort characteristics. This allowed age-specific health status and mortality to be assigned. The values used for age-specific general population utility and mortality are taken from large United Kingdom (UK)-based studies and routine datasets.(6) As the models simulate cohorts, rather than individuals, a single mean age and sex-split is specified for each. Where sex-specific parameters are used (e.g. for general population life expectancy), we use an average of values for men and women, weighted according to the stated proportion of each in the overall cohort.

1.3 PROBABILITY OF HARM

The absolute risk of harm associated with HPEs was derived from large population-based observational studies, wherever possible. These data were used to generate the probability of serious harm outcomes in the presence and absence of an HPE, adjusted to the 3-month cycle length if necessary.

1.4 RESOURCE USE AND COSTS ASSOCIATED WITH HARM

Where possible data from large population-based observational datasets were used to estimate resource use associated with harm. If these were not available, we utilised expert opinion to estimate resource use. Where possible, disaggregated resource use data were used. We attached UK reference unit costs to resource use to construct a total cost for each health state.

Unit costs associated with these resources were estimated from the National Health Service (NHS) electronic Drug Tariff (7), Personal Social Services Resource Use (PSSRU): Unit costs of health and social care (3), and NHS reference costs database.(8) The most up-to-date (at the time of analysis) unit cost databases were used, this was 2020/21 for NHS/PSSRU unit costs and March 2021 for the NHS Drug Tariff. For hospital treatment, the most up-to-date NHS reference costs reflected the 2019/20 financial year; we used these and uplifted them to 2020/21 values using PSSRU 2020/21 inflators. The costs included in the model are summarised in Table 6.2.

1.5 UTILITY AND DISUTILITY ASSOCIATED WITH HARM

We used published estimates of health status to attach a utility to each health status in the Markov model. Preferentially, we used utility estimates derived from UK populations using (Euroqol-5 dimension 3-level) EQ-5D-3L(9) or (Euroqol-5 dimension 5-level) EQ-5D-5L(10) for generic health status as the utility elicitation method, with UK tariffs, using the National Institute for Health and Care Excellence (NICE)-recommended 5L to 3L crosswalk algorithm to estimate QALYs(11, 12) as recommended in the NICE reference case (13). If the utility estimates were derived from non-UK populations, we used UK tariffs where possible.

The probability, cost and utility data are summarised in Tables 6.1 and 6.2 in this Appendix.

1.6 LENGTH OF TIME EXPOSED TO HAZARDOUS PRESCRIBING

In the absence of empirical evidence around usual length of exposure to the HPE without an event precipitating review, length of exposure to the HPE in each model was discussed with clinical experts on the team. Length of exposure to the HPE was assumed to be the lifetime of the model, unless the patient has an adverse event, which would lead to corrective action and the hazardous prescribing being corrected in some way Therefore "time to routine correction of HPE" can be defined as 10 years, unless the patient has an adverse event, and the hazardous prescribing being corrected.

However, it may also be possible that the HPE would be detected and corrected in routine care (e.g. following an annual medicines review). Therefore, we explore the impact of assuming shorter average exposure to the HPE in sensitivity analysis.

1.7 ANALYSIS

The difference in QALYs gained between a cohort who did and did not experience a HPE was determined by subtracting the QALYs gained in the no-HPE cohort from the HPE cohort:

$\Delta QALYs = QALYs_{HPE} - QALYs_{no\text{-}HPE}$

The difference in costs incurred between a cohort who did and did not experience a HPE was determined by subtracting the QALYs gained in the no-HPE cohort from the HPE cohort:

$\Delta Cost = Cost_{HPE} - Cost_{no\text{-}HPE}$

Models were built in Excel. Each model was populated with probability, cost and health status data, to allow for the generation of the point estimates and distributions of discounted outcomes (QALYs) and NHS costs in a cohort exposed to a specific type of HPE, and a cohort not exposed. Distributions appropriate for input parameters were chosen (14). If no measure of uncertainty was available for the beta or gamma distribution, we assumed that the standard deviation defining the distribution equals 20% of the mean. The probabilistic analysis was based on 10,000 samples.

1.8 SENSITIVITY ANALYSIS

The following parameters were varied in the HPE models to generate further estimates of costs and outcomes: Time horizon: 5 years, 20 years, no discount rate, duration of HPE exposure varied from 1-10 years.

We also carried out the following sensitivity analyses:
Our primary analysis assumes effects are additive (i.e. people exposed to multiple HPEs may experience multiple harms). Sensitivity analysis was carried out to minimise the overlap by deducting the number of people at risk for anticoagulants, heart failure, and CKD from the group of people over 65 who are at GI risk so that people aged over 65 will only be at risk of gastrointestinal harm if they are not part of the oral anticoagulant, heart failure, or kidney disease cohorts.

1.9 SUMMARY OF ASSUMPTIONS USED IN THE MODELS

Table 1.1: Key assumptions affecting models of NSAID-relating hazardous prescribing and/or calculations by which they are combined and scaled up to English population

- 10-year time horizon, i.e. all relevant impacts are likely to arise within 10 years[a,b,c,d,e*]
- 3-month cycle length, i.e. relevant events can be captured by simulating people's status on a 3-monthly basis[a,b,c,d,e]
- Only one type of event can occur per 3-month cycle[a,b,c,d,e]
- The hazardous prescribing will remain in place indefinitely, unless an adverse event occurs, at which point the HPE will be identified and corrected[a,b,c,d,e]
- Cohorts start in a state representing no adverse events[a,b,c,d,e]
- All risks remain constant over duration of exposure to hazardous prescribing (though some, e.g. probability of death, increase with the age of the cohort)[a,b,c,d,e]
- No assumptions are made about the underlying reason for NSAID prescription[a,b,c,d,e]
- NSAIDs are not associated with distinctive benefits, measurable in QALYs, that cannot be matched by alternative medicines[c,d,e]
- A patient' quality of life was only assumed to be affected by an NSAID if one of the events in the models occurred.[a,b,c,d,e]
- General population mortality applies, other than as a result of adverse events with a risk of death[a,b]

- The ratio between expected mortality in different age categories of the general population can be applied to mortality data from the affected population to derive age-stratified, condition-specific probabilities of death[c,d,e]
- Health-related quality of life associated with a given health state or event can be estimated by applying additive disutilities to age- and sex-stratified expectation for the UK population[a,b,c,e]
- As the most prescribed NSAID, naproxen represents a reasonable proxy for all NSAIDs; when varied in sensitivity analysis, this assumption affects costs[c,d,e] and impact on adverse events[d]*. Prevalence of HPEs is based on all NSAIDS prescribed in the study population.
- For people receiving gastroprotection while taking NSAIDs, we assume costs of omeprazole and effects of all proton pump inhibitors pooled in published meta-analyses[a,b]
- Only symptomatic ulcers incur heath service costs and impair quality of life[a,b,c]
- A serious GI event is one that involves hospitalisation for bleeding or perforation[a,b,c]
- Most GI events affect people for a single cycle (3 months), but some last for 2 cycles (6 months); all events are considered resolved by then, though people remain at risk of repeat events[a,b,c]
- If people recover from an adverse event, it has no long-term impact on risk of future events[a,b,c,d,e]
- GI discomfort and exacerbations of heart failure that can be managed in primary care are not associated with an increased risk of death[a,b,c,d]
- Any effect hazardous prescribing has on mortality is captured via the modelled adverse events[a,b,c,e]*, with the exception of population d, in which the base case assumes an additional risk of death in people exposed to NSAIDs[d] *
- Impacts of hazardous prescribing in each population are independent and additive (i.e. any people who are in more then one population face the risk of all relevant events and the expected cost and QALY impacts of each) [a,b,c,d,e]*
- The sample of practices providing HPE data (aggregate n = 10,906,453) is representative of the population of England given the geographical spread of practices (across England) and size of the sample [a,b,c,d,e]
- The number of people registered with a GP practice in England is used to scale up the impact (even though the estimate is known to double-count some people), on the assumption that the extent of double-counting is approximately equivalent in the study from which the prevalence of HPEs is derived and other practices in England [a,b,c,d,e]*

Superscript letters indicate the modelled population each assumption affects:
[a] NSAID in older people without gastroprotection
[b] NSAID with previous peptic ulcer without gastroprotection
[c] NSAID with oral anticoagulant
[d] NSAID with heart failure
[e] NSAID with chronic kidney disease
* Base-case assumption subject to sensitivity analysis

2. PRESCRIPTION OF AN ORAL NSAID, WITHOUT CO-PRESCRIPTION OF AN ULCER HEALING DRUG, TO A PATIENT AGED ≥65 YEARS (A) OR TO A PATIENT WITH A HISTORY OF PEPTIC ULCERATION (B)

This section describes the model development for: Prescription of an oral NSAID, without co-prescription of an GPA, to a patient aged ≥65 years; Prescription of an oral NSAID, without co-prescription of an GPA, to a patient with a history of peptic ulceration. These two HPEs required the same model structure populated with different parameters, so are reported together in this section.

2.1. BACKGROUND

The most frequent causes of peptic ulcer disease are *Helicobacter pylori* infection and the use of NSAIDs (15). Risks of bleeding are increased when the drug is prescribed long-term (16). Older adults (≥65 years) are at increased risk of gastrointestinal (GI) complications when taking NSAIDs. Following a GI bleed there are higher rates of hospitalization, morbidity and mortality when compared with younger patients and age is an independent risk factor for mortality in upper GI bleeds (17). In people with a history of peptic ulceration, NSAID use is a risk factor for the recurrence of peptic ulcers and complications such as rebleeding (18). Current guidance in England is varied with the British National Formulary recommending a GPA should be prescribed to older adults or those with a history of peptic ulcer (19), whilst some specify that when offering long-term (more than 3 weeks) treatment with an oral NSAID, the drug selected should be co-prescribed with a GPA (16, 20). There is evidence that the co-prescription of GPA, such as proton pump inhibitors (PPIs), is associated with significant reduction in the risk of GI events (21, 22).

2.2. DEVELOPING THE MODEL

For this model, individuals in the model cohort are at high risk of a GI event (1: over 65s; or 2: history of peptic ulceration) and prescribed NSAIDs long-term, either with (non-HPE) or without (HPE) co-prescription of a gastroprotective drug (assumed to be a PPI).

A similar HPE (involving NSAIDs and GI events) was included in an earlier economic evaluation as a Markov model which was used as a starting point for developing the new model (23). Literature searches were conducted to identify any developments in more recent models in this area and more recent data to inform model parameters so that the resulting model reflects current thinking and evidence.

2.3. LITERATURE SEARCH

The search terms used in the development of this model are reported in Table 2.1. To identify input parameters associated with the chosen health states, the literature was searched for serious GI events, GI discomfort, symptomatic ulcer and stroke. Search terms for adverse GI events were developed using published search criteria of functional dyspepsia from Agah et al. (2020) (24) and the diagnoses defining GI discomfort in the MUCOSA trial that were diarrhoea, abdominal pain, dyspepsia, nausea, vomiting and flatulence. Because, for symptomatic ulcer, various subheadings were available in Medline and Embase, the search was mainly based on those with the addition of keywords on typical locations in the upper GI tract with ulcerations, such as gastric ulcer. To identify studies looking at mortality, search terms were used from Tian et al. (2020) (25). The search terms for NSAIDs included general terms for NSAIDs and specific terms for the most common seven NSAIDs according to the 2009 NHS NSAID safety audit (26) and MeSH terms/subheadings for these if available in Medline or Embase. In addition, three existing health technology assessment reports/systematic reviews relevant to NSAIDs and GI events/toxicity were identified (27-29), the search strategies from these reviews also informed the search terms.

Table 2.1. Literature searches conducted

Searches	Search terms
Existing models (including costs and utility values)	gastrointestinal OR ulcer OR dyspepsia<br>OR<br>(proton AND pump AND inhibit*) or gastroprotect* or (ulcer AND healing) or burimamide or cimetidine or famotidine or metiamide or misoprostol or nizatidine or omeprazole or ranitidine OR (histamine AND H2 AND antagonist*)<br>AND<br>Markov AND model

	gastrointestinal OR ((proton AND pump AND inhibit*) or gastroprotect*)
Risks (impact of PPIs)	(NSAID* OR (nonsteroidal AND anti-inflammatory) OR (non-steroidal AND anti-inflammatory) OR ibuprofen OR diclofenac OR naproxen OR celecoxib).ab<br>AND<br>(proton AND pump AND inhibit*) or gastroprotect* or (ulcer AND healing) or burimamide or cimetidine or famotidine or metiamide or misoprostol or nizatidine or omeprazole or ranitidine OR (histamine AND H2 AND antagonist*).ab
Utility values	((EQ-5D*) or (SF-12) or (QALY*) or (quality-adjusted*)).ab.<br>AND<br>(gastrointestinal OR ulcer OR dyspepsia).ab

2.4. EXPERT CONSULTATION

Key uncertainties remaining after the literature reviews centred on:

- What is the likelihood of recurring/unresolved GI events?
- For how many consecutive cycles would people plausibly remain in GI event state before the event is resolved?
- Does the level of resource use associated with GI events reflect current practice?
- What is the most common means of H. Pylori testing?

The model structure and key assumptions were discussed with the multi-disciplinary members (including general practitioners (GPs), pharmacists, and lay collaborators) of the study team.

2.5. MODEL STRUCTURE

The literature searches identified seven Markov models which examined the impact of gastroprotection on GI events in people prescribed NSAIDs (23, 30-35). The health states in our model were based our previous work, (23), the newer models identified through literature searches, and expert input. Since our previous model was developed, there has been work to suggest that use of NSAIDs can increase the risk of cardiovascular (CV) adverse events (36, 37). The increased risk of CV events which may be relevant to this model would relate to exposure to NSAIDs rather than absence/presence of gastroprotection. For this model, both the HPE and non-HPE cohorts will have comparable exposure to NSAIDs and only the presence/absence of gastroprotection will be different. Therefore, it is not relevant to include CV events in this model.

Figure 2.1. Final model structure prescription of an oral NSAID, without co-prescription of a gastroprotective agent, to a patient aged ≥65 years or to a patient with a history of peptic ulceration

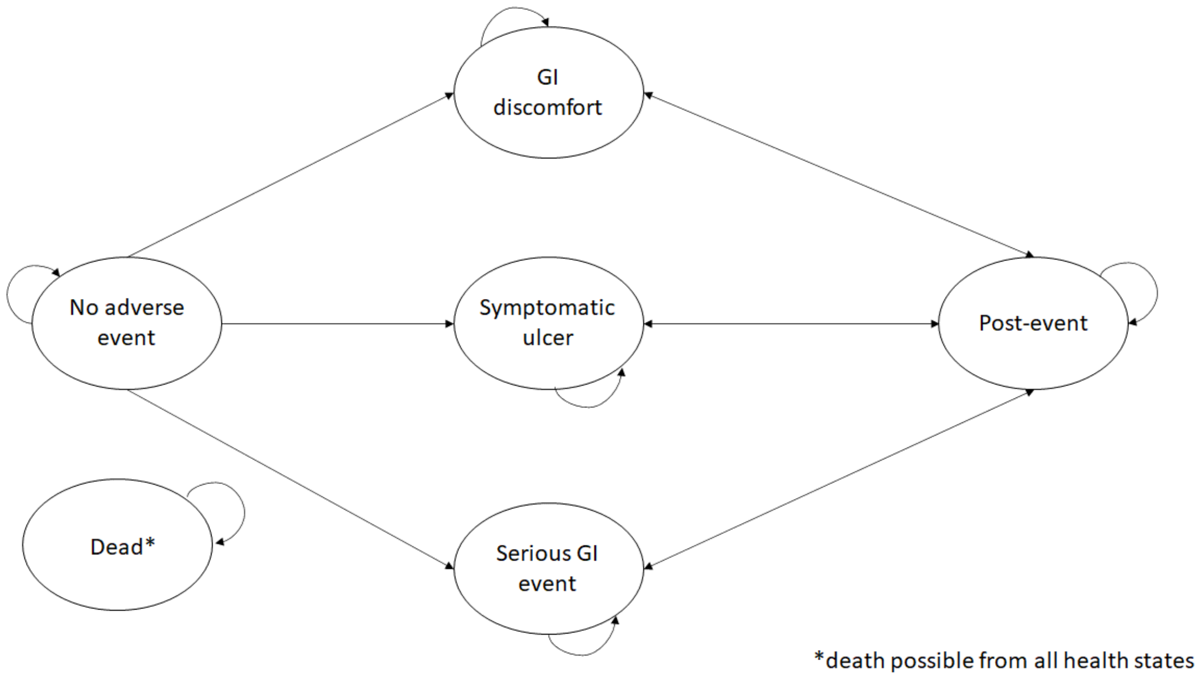

Figure 2.1 describes the model structure, and assumes that there is no long-term impact on the risk of having a subsequent GI event once any initial event is resolved (i.e. from the post-event health state).

In this model, the probability of adverse GI events is adjusted according to the presence (no HPE cohort) or absence (HPE cohort) of a gastroprotective agent to allow calculation of the differential effect on outcomes and costs. All people have a risk of death, according to the health state they are in. "Dead" is an absorbing state.

2.6. HEALTH STATES

The health states are summarised in Table 2.2.

Table 2.2. Health states used in model of prescription of an oral NSAID, without co-prescription of an ulcer healing drug, to a patient aged ≥65 years or to a patient with a history of peptic ulceration

No adverse event	This is the point of model entry.<br>Model 1 - people aged over 65 years who are prescribed an NSAID<br>Model 2 - people with a history of peptic ulceration who are prescribed an NSAID<br>No assumptions are made regarding the underlying reason for NSAID prescription.
GI discomfort (27)	People experience GI discomfort (e.g. dyspepsia) requiring treatment by a primary care physician.
Symptomatic ulcer (27)	People have a clinical diagnosis of a peptic ulcer following adverse GI symptoms, which may include endoscopic confirmation.
Serious GI event (27)	People experience a serious GI event including perforation, bleed (including melaena), or obstruction.
Post-event	Following resolution of a GI event, people return to a health state where the long-term risk of subsequent events is not increased but they can still experience further events.

Dead	There is a risk of death from all health states.

Description of health states adapted from Brown et al (2006) (27)

2.7. COHORT CHARACTERISTICS

Cohort characteristics were selected to reflect as closely as possible the target population in the PINCER/Optimise Rx interventions.

2.7.1. Query A - patients aged 65 years and over

This query specifies that the model cohort are aged over 65. According to census data for the UK, the mean age within this group is 70 (38) therefore the age at model entry for this query is assumed to be 70 years. As we have made no assumption about the reason for the NSAID prescription, the cohort is assumed to have health utility values and background mortality rates (i.e. when death is unrelated to GI event) typical of the general population in this age group as this would include people with a range of health conditions.

2.7.2. Query B - patients with a history of peptic ulcer

The at risk group for this Query includes anyone aged 18 years and over with a history of peptic ulcer. Ulcer incidence is negligible in people under the age of 55 (39) therefore the age at model entry was assumed to be 55 years to reflect that, while minimal, younger people are present in the at risk group. Although this cohort are younger at model entry than in Query A (and so would typically be at lower risk of the GI events), it is assumed that because of their history of peptic ulcer their risk of subsequent GI events are the same as for the older cohort in Query A. The cohort is assumed to have age-specific health utility values (i.e. higher than for Query A) and background mortality rates (i.e. lower than for Query A) typical of the general population.

2.8. TRANSITION PROBABILITIES

The transition probabilities used in the model are described in this section and summarised in Tables 3-5. The literature search to source the probabilities of health events and the impact of NSAIDs on these probabilities is provided in Table 2.1. There were few recent studies of the relationship between NSAIDs, gastroprotection, and adverse GI outcomes therefore although many of the source studies were quite old they were still assessed to be the highest quality evidence available. The source studies identified tended to report the rate of adverse GI events in the absence of gastroprotection (i.e. relevant to the HPE model) therefore these parameters were identified and then the relative risk of events in the absence/presence of gastroprotection was used to derive the transition probabilities for the non-HPE model. The estimation of the probability of death from the different health states is described below and summarised in Table 2.3.

2.8.1. Probability of GI discomfort in presence of NSAIDs without gastroprotection and probability of death from event

A paper published in 2009 synthesised data from 3 large randomised controlled trials (RCTs) evaluating GI events in people taking NSAIDs (which at the time were the largest RCTs in the area) (40). For people taking naproxen the rate of GI discomfort (dyspepsia) per 10,000 person years at risk was 6481 (95% CI 6091 to 6883) (40). This rate was used to derive a 3-month probability. It was assumed that following a primary-care managed episode of GI discomfort there would be no increase in the risk of death therefore age-adjusted general population mortality rates, according to the Office for National Statistics (41), were applied to this health state.

2.8.1.1. Probability of recurring GI discomfort in subsequent cycle

No literature was identified regarding the short-term recurrence/resolution of GI discomfort in the presence of NSAIDs. Following consultation with clinicians, it is assumed that once

somebody enters the GI discomfort state, their risk of recurring dyspepsia for the following cycle is the same as the risk of recurring serious GI events for which there is some observational data (see below). After a maximum of two cycles of dyspepsia it is assumed that it is resolved, and the person enters the post-event state.

2.8.2. Probability of symptomatic ulcer in presence of NSAIDs without gastroprotection and probability of death from event

A paper which pooled data from 3 large RCTs (40) was used to estimate the 3-month probability of a symptomatic ulcer in the model. For people taking naproxen the rate of symptomatic ulcers per 10,000 person years at risk was 112 (95% confidence intervals (CI) 66 to 169) (40). An observational study collected data across a Danish county (population 470,000) between 1993 and 2002 (42). In the first month following an uncomplicated (i.e. non-bleeding) gastric ulcer, the standardised mortality rate (i.e. factor by which the expected number of deaths in the absence of an ulcer is multiplied to get the observed number of deaths) was 11.6 (95% CI 9.6-13.9) (42). A Finnish observational study collected data on 4,154 patients with uncomplicated peptic ulcers between 2000 and 2008. The reported standardised mortality ratio was 2.16 (95% CI: 2.05; 2.29) at one year (43). In our base case, we used a weighted average of these numbers, assuming the higher one applied for the first 30 days and the lower for the remainder of the cycle – i.e. (11.6 × 30 + 2.16 × [91.3 – 30]) / 91.3, where 91.3 is the number of days in a 0.25-year cycle). This value was multiplied by the probability of death in the general population (Office for National Statistics (41)) to estimate the increased probability of death in the first cycle post-ulcer (after which the probability of death is assumed to return to that in the general population).

2.8.2.1. Probability of recurring symptomatic ulcer in subsequent cycle

No literature was found which reported a robust estimate of the probability of short-term ulcer recurrence. A model by Pettit et al assumed that the likelihood of ulcer recurrence was the same as for serious GI events (bleeds) (44). Following consultation with clinical experts, this was considered a reasonable assumption, therefore it was assumed that the likelihood of ulcer recurrence was the same as for serious GI events in this model (see below).

2.8.3. Probability of serious GI event in presence of NSAIDs without gastroprotection and probability of death from event

The NHS medication safety dashboard reports quarterly data for England, from 2015 to present (2019/20 Q3), on the number of people over 65 who are prescribed an NSAID without gastroprotection and then how many of those people are admitted to hospital with a GI bleed (45). According to these data, an aggregate of 3,259 events occurred over 948,099 patient-yrs. This equates to a 3-month probability of a bleed in the presence of the HPE of 0.09%.

A UK-based study published in 2012 by Crooks et al analysed linked primary and secondary care records to explore the GI bleeding and mortality (46). They reported a 28-day mortality rate of 8.1%. This was used to estimate the probability of death following a serious GI event for the 65-69 age group (the mean age of people experiencing a GI bleed was 68 according to the 2007 acute upper gastrointestinal bleeding (AUGIB) UK Audit (47)). The probability of death for the other age groups was estimated from this value and the relative risk of death by age in general population according to the Office for National Statistics (41). An alternative potential data source for the probability of death following serious GI event was also identified, a meta-analysis of studies published 1997-2008 (48). This paper reported a similar mortality rate to the Crooks study, 7.4% (95% CI 7.2-7.6).

2.8.3.1. Probability of recurring serious GI event in subsequent cycle

A systematic review and meta-analysis of complicated (i.e. bleeding/perforated) peptic ulcers reported that the average 1-month recurrence rate (of bleeds) was 15.8% (95% CI: 11.0–20.5), that approximately 90% of recurrent bleeds occur in the first 7 days (18). These data were used in the model to estimate the probability of remaining in this health state for consecutive cycles, with the assumption that the one-month rate of recurrence was the same as the 3-month rate (because 90% happen in the first 7 days). An observational study reported that the risk of rebleeding was highest in the first 3 months (18, 49). Therefore people were permitted to remain in the serious GI event state for one additional cycle, after which they move to the post-event state.

2.8.4. *Probability of death (from the no event and post event states)*

A large meta-analysis reported that among NSAID users, the absence or presence of gastroprotection was not significantly associated with all-cause mortality (gastroprotection versus no protection: OR 0.95 (95% CI 0.50−1.81)) (22). Therefore in the model the risk of death (from the no event and post event states) is assumed to be equivalent to age-specific general population risk according to the Office for National Statistics (41).

The probability of death from each health state is summarised in Table 2.3.

Table 2.3. Age-specific 3-month probability of death for GI model health states

**Health state**	**Age group (years)**					**Source**
	55–59	60–64	65–69	70–74	75–79	
No event/ post event	0.0012	0.0019	0.0030	0.0047	0.0083	Office for National Statistics, 2018 (41)
GI discomfort	0.0012	0.0019	0.0030	0.0047	0.0083	Office for National Statistics, 2018 (41)
Symptomatic ulcer	0.0064	0.0099	0.0157	0.0244	0.0429	Observational study reporting post-ulcer SMR (42)
Serious GI event	0.0328	0.0510	0.0810	0.1268	0.2242	Analysis of primary and secondary care electronic records reporting 1-month post-event mortality for 65-69 age group (46)

GI: gastrointestinal; SMR: standardised mortality ratio

2.8.5. Detection and correction of HPE

We assume that, for people exposed to the HPE (NSAID prescription without a gastroprotective agent), the HPE will be identified following an adverse GI event. Once the HPE is identified, it is rectified through co-prescription of a gastroprotective agent (a proton pump inhibitor (PPI), in line with current guidelines (20)). Both cohorts (HPE and non-HPE) have the same probability of an adverse GI event once the HPE has been corrected; this approach has been used in other Markov models in this area (30).

2.8.6. Impact of absence/presence of gastroprotection on the probability of adverse event

As noted above, the absence or presence of gastroprotection was not significantly associated with all-cause mortality (22). Therefore, the probabilities affected by the presence or absence of the HPE are: GI discomfort, symptomatic ulcer and serious GI event. The probability of these health events in the absence of gastroprotection (i.e. the HPE condition) has been outlined above. This section summarises the evidence used to generate the probability of these health

events when a gastroprotective agent has been co-prescribed with NSAIDs (i.e. the non-HPE condition).

A meta-analysis of four studies comparing dyspepsia between NSAID/PPI combination and NSAIDs alone estimated a 66% relative risk reduction for NSAID/PPI (21). A recent meta-analysis reported pooled odds ratios for GI events (symptomatic ulcers and bleeds) in the absence and presence of a gastroprotective agent (22). The results from these two studies are summarised in Table 2.4.

Table 2.4. Impact of presence of PPI (i.e. risk reduction) on transition probabilities

Description	OR/RR (95% CI)	Reference
Decreased likelihood of GI discomfort	RR 0.34 (0.22-0.54)	Spiegel et al (21)
Decreased likelihood of symptomatic ulcer	OR 0.28 (0.17-0.46)	Scally et al (22)
Decreased likelihood of serious GI event	OR 0.35 (0.24-0.52)	Scally et al (22)
Decreased likelihood of death	OR 0.95 (0.50−1.81), There is no increased likelihood of death applied in the model	Scally et al (22)

GI: gastrointestinal: OR = odds ratio; RR = risk ratio

The values in Table 2.4 were combined with data in the sections above to generate the probabilities of events in the non-HPE cohort i.e. accounting for the protective effect of gastroprotection. The transition probabilities used in the model for both the HPE and non-HPE conditions are summarised in Table 6.1.

2.9. COSTS

It was assumed that people in all health states (other than dead) and both cohorts (HPE and non-HPE) would receive comparable treatment with NSAIDs (e.g. specific drug prescribed, duration etc) therefore no costs were included for this. An additional cost was included in the non-HPE model and in the post-event state for the cost of the PPI prescription. The NICE guidance for dyspepsia management recommends a dosage of omeprazole 20mg/day which costs £1.18 for 28 days (7). A paper published in 2015 followed a UK-based cohort of people admitted to hospital with a GI bleed and reported the costs associated with their initial inpatient admission and 28-days post discharge (£2851, cost year 2012/13) (50). This was inflated according to the hospital & community health services (HCHS) index and the new NHS Cost Inflation Index (NHSCII) (51) and used to inform the cost for the serious GI event health state. Previous work by Elliott et al provided relevant disaggregated resource use data for GI discomfort and symptomatic ulcer (52), however this paper was from 2006 therefore expert opinion was sought from clinical members of the project team to confirm whether the more recent estimate reflects current practice.

2.10. UTILITIES

The utility values used in the model are reported in Table 6.2. For the “prescribed NSAIDs” and “post event” health states, age-specific utility values from the English general population were used. These were derived from the 3-level EQ-5D which was collected for over 40,000 people across four waves (2003, 2004, 2005 and 2006) of the Household Survey for England (HSE) (53). The HSE is a well-established annual survey conducted on randomly selected samples of the population living in private households in England. The utility value for the dead health state was 0.

2.10.1. GI discomfort

The Euro heart survey collected data on quality of life in patients with AF, the most common diagnosis associated with the use of OACs, in 35 European countries from 2003 to 2004 (54). The study used the EQ-5D-3L questionnaire and results were translated into utilities using UK tariffs for the different dimensions. The Euro heart survey estimates have been used in other peer-reviewed models (55, 56) and a NICE technology appraisal (57, 58). Mean age of participants in the survey was slightly lower than the start age of this model with a mean age of 66 years. Percentage of men (60%) and women (40%) was very similar to the proportions in this model population. The majority of patients (68%) were prescribed OACs. A mean utility was reported of 0.779 (standard deviation (SD) 0.253). This utility was used to calculate QALYs generated in the "No adverse event" health state in the HPE and non-HPE model and in the Post-GI event state. To account for quality of life decreasing with age, the utility was multiplied by the ratio of the utility for a given age range relative to a reference age (66–70 years), based on general population utilities estimate. This method to account for the decreasing utility with age, was also used in a HTA (57). The relative relationship between the general population estimate from the English household survey for the age group 66-70 years, and the utility estimate from Berg et al, was used to calculate age dependent utilities.

2.10.2. Symptomatic ulcer

We calculated disutility for this state using EQ-5D-3L values from the Health Surveys for England for people with 'other digestive complaints (stomach, liver, pancreas, bile ducts, small intestine duodenum, jejunum and ileum)' (53). The 666 respondents who reported experiencing complaints of this type had a mean EQ-5D-3L of 0.734 (95%CI: 0.707 to 0.760), whereas respondents of a similar age not affected by the health condition (n=620) reported a value of 0.836 (95%CI: 0.815 to 0.856). This equates to a decrement of −0.102 (95%CI: −0.136 to −0.068). For comparison, a 2001 study included a time trade-off study and reported that the QALY-loss associated with a peptic ulcer was 0.11 (59), and the previous PINCER model included a decrement of −0.13(23, 60).

2.10.3. *Serious GI event*

A recent systematic literature review of utility decrements associated with bleeding events in people taking dual antiplatelet therapy reported that for 'gastrointestinal bleeds' the QALY-losses reported in the literature identified ranged from 0.005 to 0.016 (61). Campbell et al. (2015) reported utility values derived from EQ-5D-3L for a cohort of 936 participants who were admitted to one of six UK university hospitals with an upper GI bleed (50). The participants completed the EQ-5D 28 days after they were admitted. The mean age of the sample was 59.4 years; the general population mean utility value for the 55 to 60 group is 0.8222 (53). In the Campbell study the utility value for those who survived to 28 days was 0.735 (SD 0.02). Using these values to calculate a decrement (i.e. 0.8222 − 0.735 = 0.09) would imply less impact on quality of life than the utility decrements we use for symptomatic ulcer and a similar level of disutility as for GI discomfort. We judged this to lack face-validity. For the previous PINCER model the authors estimated a greater utility decrement (0.18); however, this was based on values originating from an obsolete US instrument with unclear valuation methods (23) (60).

In our base case, we use an estimate from Doble et al. (2018). The authors elicited EQ-5D values from people with experience of DAPT – and, in some cases, experience of DAPT-related bleeding – using vignettes describing minor and major bleeding events (61). When using EQ-5D-3L, they found that a major bleed is associated with a decrement of −0.239 (95%CI: −0.384 to −0.0933). The authors also elicited participants' opinions about the likely length of impairment (their average guess was 45.38 days); however, we do not use these data, instead applying the

disutility to a full, 3-month cycle of our model. We have multiple reasons for doing so: (i) the participants had no experiential basis on which to provide an estimate, and – unlike the vignette describing the severity of the imagined incident – no other support to guide their answer; (ii) the vignette describes a bleeding event that is somewhat less serious than the defined state in our model (it describes an event that requires some sort of medical advice and/or treatment, whereas we envisage hospital admission will be necessary in every case); (iii) we know from other evidence that disutility from GI bleeds last for longer than 45 days (e.g. Wang et al. (2017)(62) show persisting deficits at 12 months after a major GI bleed). Therefore, we judged that applying the decrement to a full cycle would represent an appropriate level of disutility for the type of event our model simulates.

2.10.4. Disutility of correcting HPE

Correction of this HPE is assumed to involve the additional prescription of a gastroprotective agent rather than discontinuation of the NSAID. There should therefore be no disutility from correcting this HPE.

3. Patients aged ≥18 years prescribed an oral anticoagulant (OAC) in combination with an oral NSAID (Query D)

3.1. DEVELOPING THE MODEL

The cohort consists of patients at high risk of GI bleeding that are prescribed an OAC. A literature search identified no previous decision analytic models comparing patients with concomitant OAC and NSAID treatment with patients treated with OACs but without NSAID treatment.

Because the exposure to HPE for this model is defined by the concomitant treatment with NSAID, the effect of NSAID exposure and non-exposure needed to be captured in the model structure. That is why literature on decision models on NSAID use was reviewed. A similar HPE (involving NSAIDs and GI events), but with a different cohort was included in the original PINCER economic evaluation as a Markov model which was used as a starting point for developing the new model (23). Literature searches were conducted to identify any developments in more recent models in this area and more recent data to inform model parameters so that the resulting model reflects current thinking and evidence. To get an understanding of how disease progression and treatment in the underlying denominator population with OACs is usually modelled a literature review on models comparing different OAC treatments in AF was conducted. To get an understanding of how model designs in cohorts using OACs incorporated GI events the literature around decision analytic models assessing the cost-effectiveness of OAC treatments was screened.

3.2. LITERATURE SEARCH

Search terms for the literature searches conducted are reported in **Table 3.1**. Search terms describing OAC and NSAID users were based on all medications relevant for these drug groups. Search terms for OACs were based on the drug substances available in the UK and the main diagnoses of OACs.

Table 3.1. Literature searches conducted

Searches	Search terms

Markov models comparing NSAID use in anticoagulated patients	Medline, Embase, Scopus: search terms for the concepts: (1) Population: "Antithrombotics (oral anticoagulant/antiplatelet)"; (2) Intervention/Exposure: "Hazardous prescribing"; (3) Outcome: "bleed"; (4) study type I: "decision analytical model"; (5) Study type II: "Health economics". Search terms were mainly derived from the Centre for Reviews and Dissemination (63) as suggested by NICE (64), from previous NICE guidelines (65-67).<br>HTA database: ((rivaroxaban or dabigatran or apixaban or edoxaban or warfarin) OR (doac or NOAC or oral anticoagulant or atrial fibrillation)) AND ((meloxicam or naproxen or ibuprofen or diclofenac or mefenamic or celecoxib or etoricoxib) OR (NSAID or anti-inflammatory drug or coxib or cyclooxygenase inhibitor or cyclooxygenase 2 inhibitor))
Markov models of NSAIDs	Medline, Embase, Scopus:: (Modeling or modelling or Model or Markov or transition or markov chains). Mp AND (Economics, Pharmaceutical/ or Costs and cost analysis/ or budgets /or cost-benefit analysis/ or (cost or pharmacoeconomic or value for money or budget or cost-effectiveness or cost-effectiveness analysis).mp) AND<br>AND ((meloxicam or naproxen or ibuprofen or diclofenac or mefenamic or celecoxib or etoricoxib) OR (NSAID or anti-inflammatory drug or coxib or cyclooxygenase inhibitor or cyclooxygenase 2 inhibitor))<br>HTA database: (meloxicam or naproxen or ibuprofen or diclofenac or mefenamic or celecoxib or etoricoxib) OR (NSAID or anti-inflammatory drug or coxib or cyclooxygenase inhibitor or cyclooxygenase 2 inhibitor)
Markov models of OACs	Medline, Embase, Scopus: ((Modeling or modelling or Model or Markov or transition).mp or markov chains) AND (Economics, Pharmaceutical/ or Costs and cost analysis/ or budgets /or cost-benefit analysis/ or (cost or pharmacoeconomic or value for money or budget or cost-effectiveness or cost-effectiveness analysis).mp) AND<br>(Atrial Fibrillation/ or Anticoagulants/ or Warfarin/ or Factor Xa Inhibitors/ or rivaroxaban/ or dabigatran/ (oral anticoagulant or doac or NOAC or edoxaban or rivaroxaban or apixaban or dabigatran<br>dabigatran or Eliquis or Pradaxa or Xarelto or lixiana).mp
Risks (impact of NSAIDs in OAC patients-GI events)	Medline, Embase, Scopus: (((NSAID or anti-inflammatory drug or coxib or cyclooxygenase inhibitor or cyclooxygenase 2 inhibitor) or (meloxicam or naproxen or ibuprofen or diclofenac or mefenamic or celecoxib or etoricoxib)).ab. or Anti-Inflammatory Agents, Non-Steroidal/<br>Cyclooxygenase 2 Inhibitors/ or Cyclooxygenase Inhibitors/<br>Meloxicam/ or Naproxen/ or Ibuprofen/ or Diclofenac/ or Mefenamic acid/ or Celecoxib/ or Etoricoxib/)<br>AND<br>((eliquis or pradaxa or lixiana or xarelto) or<br>(doac or NOAC or oral anticoagulant or atrial fibrillation) or<br>(rivaroxaban or dabigatran or apixaban or edoxaban or warfarin)).ab. or Factor Xa Inhibitors/ or Atrial Fribrillation/ or Anticoagulants/ or anticoagulant agent/ or rivaroxaban/ or dabigatran/ or warfarin/<br>AND(<br>((((gastrointestinal or gi) and (symptom or diseases or disorder or adverse or diagnosis)) or (dyspepsia or diarrhoea or flatulence or vomiting or nausea or abdominal pain or epigastric pain)).ab. or<br>Gastrointestinal diseases/ or Dyspepsia/ or Abdominal pain/ or gastrointestinal symptoms/ or epigastric pain/)<br>OR

	(((peptic or digestive system or duodenum or gastric or stomach or esophagus or jejunum or colon or symptomatic) and (ulcer)).ab. or peptic ulcer bleeding/ or ulcer incidence/ or digestive system ulcer/ or duodenum ulcer/ or acetic acid-induced gastric ulcer/ or gastric ulcer bleeding/ or stomach ulcer/ or duodenal ulcer bleeding/ or esophagus ulcer hemorrhage/ or esophagus ulcer/ or jejunum ulcer/ or ulcer perforation/ or peptic ulcer/ or recurrent peptic ulcer/ or indomethacin-induced gastric ulcer/ or colon ulcer/ or ulcer/)<br>OR<br>((death OR died OR fatal OR mortality OR deceased OR non survivor OR non Survival).ab. or Death/ or Hospital Mortality/ or Mortality/<br>Fatal Outcome/ or Survivors/ or Survivor/ or Survival Rate/ or Survival/ or mortality rate/ or standardized mortality ratio/))<br>HTA database: ((rivaroxaban or dabigatran or apixaban or edoxaban or warfarin) OR (doac or NOAC or oral anticoagulant or atrial fibrillation)) AND ((meloxicam or naproxen or ibuprofen or diclofenac or mefenamic or celecoxib or etoricoxib) OR (NSAID or anti-inflammatory drug or coxib or cyclooxygenase inhibitor or cyclooxygenase 2 inhibitor))
Risks (impact of NSAIDs in OAC patients - cardiovascular events)	Medline, Embase, Scopus: (((NSAID or anti-inflammatory drug or coxib or cyclooxygenase inhibitor or cyclooxygenase 2 inhibitor) or (meloxicam or naproxen or ibuprofen or diclofenac or mefenamic or celecoxib or etoricoxib)).ab. or Anti-Inflammatory Agents, Non-Steroidal/<br>Cyclooxygenase 2 Inhibitors/ or Cyclooxygenase Inhibitors/<br>Meloxicam/ or Naproxen/ or Ibuprofen/ or Diclofenac/ or Mefenamic acid/ or Celecoxib/ or Etoricoxib/)<br>AND<br>(Stroke or myocardial or heart failure or cardiovascular or systemic embolism or thromboembolic).ab<br>AND<br>(Atrial Fibrillation/ or Anticoagulants/ or Warfarin/ or Factor Xa Inhibitors/ or rivaroxaban/ or dabigatran/ (oral anticoagulant or doac or NOAC or edoxaban or rivaroxaban or apixaban or dabigatran<br>dabigatran or Eliquis or Pradaxa or Xarelto or lixiana).mp

Utility values	Medline, Embase, Scopus: ((EQ-5D*) or (SF-12) or (QALY*) or (quality-adjusted*) or (qol) or quality of life) or (HRQoL)) .ab.<br>AND(((((gastrointestinal or gi) and (symptom or diseases or disorder or adverse or diagnosis)) or (dyspepsia or diarrhoea or flatulence or vomiting or nausea or abdominal pain or epigastric pain)).ab. or<br>Gastrointestinal diseases/ or Dyspepsia/ or Abdominal pain/ or gastrointestinal symptoms/ or epigastric pain/)<br>OR<br>(((peptic or digestive system or duodenum or gastric or stomach or esophagus or jejunum or colon or symptomatic) and (ulcer)).ab. or peptic ulcer bleeding/ or ulcer incidence/ or digestive system ulcer/ or duodenum ulcer/ or acetic acid-induced gastric ulcer/ or gastric ulcer bleeding/ or stomach ulcer/ or duodenal ulcer bleeding/ or esophagus ulcer hemorrhage/ or esophagus ulcer/ or jejunum ulcer/ or ulcer perforation/ or peptic ulcer/ or recurrent peptic ulcer/ or indomethacin-induced gastric ulcer/ or colon ulcer/ or ulcer/)<br>OR<br>(stroke.mp. or Stroke/)<br>OR<br>(Gastrointestinal Hemorrhage/ or gastro intestinal bleeding.mp. or Peptic Ulcer Hemorrhage/ or bleeding.mp. or Hemorrhage/ or Peptic Ulcer Hemorrhage/ Or Duodenal Ulcer/ Or Peptic Ulcer/ Or Peptic Ulcer Perforation/ Or Ulcer/ Or Stomach Ulcer/ Or Ulcer.mp.)

* HTA = health technology assessment

3.3. EXPERT CONSULTATION

Different experts were involved in the design of the model and to assess face validity of the assumptions made during the model conceptualisation. A summary of key assumptions made were sent to two GPs from the study team, to confirm these were reasonable. The final model was also presented to a patient representative in a video conference call for face validation. The patient is diagnosed with AF and is experienced in NSAID use and NSAID related adverse events.

3.4. MODEL STRUCTURE

The literature search identified no previous state-transition models comparing patients with concomitant OAC and NSAID treatment with patients treated with OACs only. An additional literature review was performed to identify state-transition models that incorporated health states following treatment with NSAIDs for patients with any health condition. There were 70 published state-transition models that compared either (i) different NSAIDs or (ii) an NSAID strategy to a non-NSAID strategy. Among the identified models on NSAIDs, nine examined the impact of gastroprotection on GI events in people prescribed NSAIDs (31, 34, 35, 68-73). Others investigated different treatment strategies including NSAIDs for lower back pain, rheumatoid or osteo-arthritis (74-79). Only two models were identified that assessed the economic impact of a type of HPE (80, 81). These models had the advantage that they described in detail, how and when actions to resolve the HPE type were taken. Moriarty et al. (2019) compared patients over 65 years of age with the hazardous prescription of an NSAID and without the NSAID. The original economic evaluation of the PINCER intervention by Elliott et al. (2014) compared patients with a history of peptic ulcer with a concomitant prescription of an NSAID or without. The latter was used as a starting point for developing the new model (68). This model was chosen because forgoing literature searches, methods and results were reported more transparently and in more detail than in Moriarty et al. (2019). The other NSAID models identified were searched for any developments in more recent models in this area and more recent data to inform model parameters so that the resulting model reflects current evidence.

While NSAIDs have always been associated with GI adverse events, their cardiovascular risk was under-recognized for a long time (82). The following sections describe how and if GI adverse events and cardiovascular adverse events were included in published state-transition models: first, in models around the exposure to NSAIDs and, second, models in the denominator population of anticoagulated patients.

3.5. GI ADVERSE EVENTS REPORTED IN NSAID MODELS

The model aims to represent key GI adverse events associated with NSAID use as health states and uses the same approach and definitions for GI health states as in Sections 1 and 2 in this appendix.

3.6. GI ADVERSE EVENTS REPORTED IN OAC MODELS

To get an understanding of how adverse events in the underlying denominator population with OACs were usually modelled a literature review on models comparing different OAC treatments in AF was conducted.

The main published models identified that were frequently used by other researchers were (i) a state-transition model by Sorensen et al. (2009) (85) used in a health technology appraisal for NICE (TA249) (86) and other studies (87-95), (ii) the model by Dorian et al. (2014) used in TA275 (96) and multiple other studies (97-104), and (iii) a structure by Gage et al. (1995) (105) that was adapted by Lee et al. (2012) (106-108). Five additional model structures were identified using a different structure from the three main published models (55, 109-111).

The review of the published models focused on how serious GI events were included in the model structure. Of the GI adverse events described in the NSAID models, only serious GI events, such as GI bleeding, were modelled in the published models on OACs. Serious GI events were mainly modelled as extracranial haemorrhages of which the majority of the events are serious GI events. Long-term impact of serious GI events was not incorporated in the structures of the three main models (85, 96, 105). Serious GI events were included as temporary events or transient states with no effect on future transition probabilities but with a case-fatality, a one-time cost and disutility in the three main published models. Only three of the five models using a different structure form the three main published models predicted a long-term effect of serious GI events in subsequent cycles. Ademi et al. (2015 and 2016) (110, 112) included a separate health state for the time after a serious GI event to account for a prolonged reduced quality of life and Sing et al. (2013) (109) and Lopez-Lopez et al. (2017) (55) expected an increased mortality in subsequent cycles. In the published models, only acute costs for the serious GI event were included. For the model, it was assumed that a serious GI event does not impact subsequent cycles with regards to healthcare costs.

3.7. INCLUSION OF CARDIOVASCULAR EVENTS

More recently, evidence has suggested that NSAIDs could be associated with an increased risk of cardiovascular events (82). For anticoagulated patients, the effect of NSAIDs on thrombotic cardiovascular adverse events, such as stroke, was of specific interest because anticoagulated patients already have an increased risk of these events. In the literature review evidence on an increased risk of stroke, systemic embolism, heart failure and MI was investigated. The evidence on an increased risk of stroke and systemic embolism associated with NSAIDs was inconclusive. The results of a cohort study in linked primary and secondary care data from England (Clincal Practice Research Datalink (CPRD)/Hospital Episode Statistics (HES)/Office of National Statistics (ONS)] indicate a significant increase of the stroke risk in the presence of concomitant NSAID therapy and no significant increase in the risk of systemic embolism.(113). The cohort

study investigated a population of OAC users with NSAID use as a time-dependent exposure. Patients with at least one NSAID during follow up were matched 1:1 with patients with no NSAID during follow up using propensity scores. Similar results were reported by Kent et al. (2018) in a subgroup of the RE-LY trial in anticoagulated patients. Consequently, stroke was included as a cardiovascular adverse event in the model. The risk of systemic embolism in both OAC, and OAC and NSAID users was low and the difference non-significant. As a result, it was not included as a cardiovascular adverse event. Heart failure (114) and myocardial infarction (MI) (115) were not significantly associated with NSAID use, so were, due to lack of evidence to suggest otherwise, not included as a health state.

3.8. FINAL MODEL STRUCTURE

The health states in this model were based on the original NSAID model used in our earlier work (23), the newer models identified through literature searches, and expert input. From literature reviews and expert panel input, the following model structure was developed [Figure 3.1].

Fig 3.1. Final model structure (Death is possible from all health states)

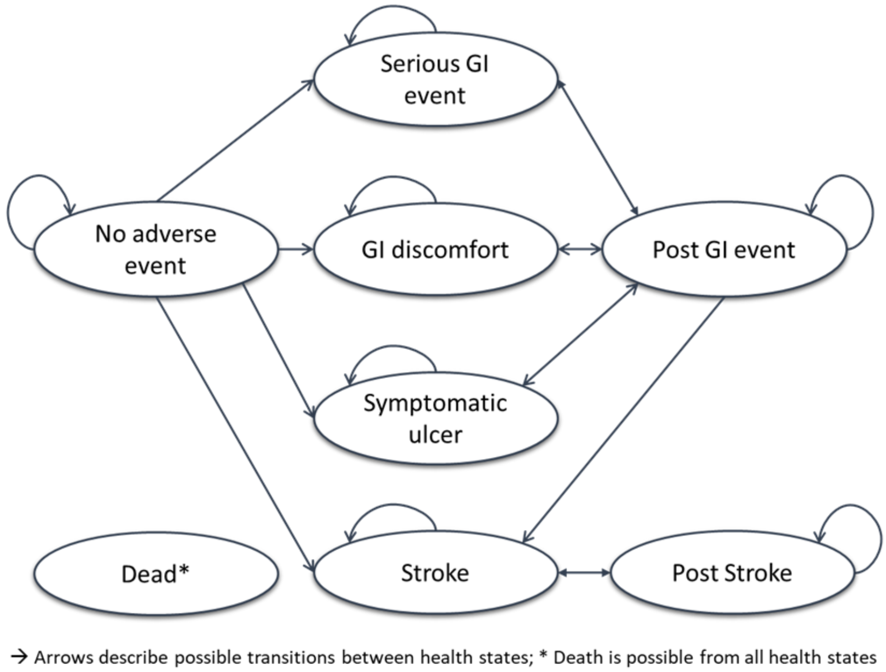

A list of assumptions with regards to the model structure is provided in Table 3.2.

Table 3.2 Summary of key model assumptions

**Model assumption**	**Justification (and approved by clinicians)**
Only one type of event can occur per three-months cycle.	In a Markov model some form of simplification has to be done. A three months cycle was considered appropriate and has been used previously in NSAID (32, 116) and atrial fibrillation (AF) Markov models for NICE technology appraisals(57, 86, 117)
All patients experiencing an adverse event stay on the	Guidelines recommend to continue OAC treatment after stroke or serious GI events (118, 119)

OAC therapy they used prior to the event	
No long-term effects of GI adverse events expected on cost, utilities and transition probabilities expected. All patients not experiencing a recurrent event or die move to the post-GI event state	There is no sufficient data to support the assumptions that there is a prolonged effect of the adverse GI events after the cycle in which the event occurred. The impact was always highest in the first... months after the event and considered negligible thereafter (this was validated by clinicians in the team)
NSAID is removed after any of the adverse events.	Clinicians suggested, that each of the adverse events, would trigger a review of the medication and discontinuation of the NSAID.
No disutility associated with removing NSAID	It was assumed that alternative treatments, such as paracetamol have the same utility, because there was no appropriate data to suggest otherwise. One model that included a disutility with paracetamol use conducted a meta-analysis of arthritis index in Ontario(120). However, it was not clear how this was achieved.
OAC is not discontinued or interrupted after any of the adverse events	Cost of interruptions of OAC treatment were considered negligible and is recommended for 7 days. Discontinuation rates associated with GI bleeding were very diverse(121-125). It was assumed that discontinuation rates were the same in the HPE and non HPE model and were therefore not considered to impact the overall results.
Only one recurrence per adverse GI event after initial event	Observational data on serious GI events and that the majority of recurrences occur in the first 3 months (126). It was assumed that this was the same for the other adverse GI events

Two cohorts are followed through the same state-transition model. The non-HPE cohort consisted of patients with OAC treatment and the HPE cohort consisted of patients with OAC and concomitant NSAID treatment. The two cohorts only differ in specific transition probabilities as described later. All patients are assumed to start in the no adverse event state. Adverse events potentially attributable to NSAID use were stroke and GI adverse events. The GI adverse events were serious GI events (GI bleeding events, ulcer perforation and ulcer bleeding), GI discomfort (diarrhoea, abdominal pain, dyspepsia, nausea, vomiting, flatulence) and symptomatic ulcer (excluding ulcers identified during endoscopy that did not cause symptoms). From all health states patients could transition to the dead state.

After entering an adverse event health state (serious GI event, GI discomfort, symptomatic ulcer or stroke), the model assumes that the HPE is resolved and the NSAID is removed in the subsequent cycle. The model structure assumes that there is no impact of the NSAID on the risk of having a subsequent adverse event. Once any initial adverse event is resolved, the patient either experiences a recurrent event or transitions to a post-event state. In the two post-event states, it is assumed that there is no increased risk of any adverse event compared with the non-HPE cohort (who received no NSAID treatment). The post-GI event state is equivalent to the no adverse event state in the non-HPE cohort with regards to transition probabilities, resource use and health related quality of life. This structural assumption was supported by the two GPs and two pharmacists consulted. All four experts agreed with the assumptions that the HPE was

preferably resolved by removing the NSAID and that following any GI adverse event, removing the NSAID would be the first choice. For stroke, the GPs indicated that they would remove the NSAID in only 50% of the cases. This was not reflected in the model because data to populate this were not available. There was no evidence on how NSAIDs increase the risk of stroke recurrences. Assuming the recurrence risk is the same, the only difference would be the cost of the NSAID, which is negligibly small. Therefore, it was considered justifiable to assume that patients moving to the post-stroke state stop the NSAID treatment if they were exposed to the HPE.

From the post-stroke state, patients could either die, experience a second stroke or remain in the health state. Stroke was considered the most severe adverse event, and no GI adverse events were possible afterwards. This approach to use stroke as a semi-absorbing state is widely used in the decision models comparing OAC treatments (97-104). In contrast, after entering the post-GI event state transitions to all other adverse event states are possible.

There are no transitions possible between the adverse event states. The adverse events are not precursor events but alarming events. Patients go to the state of the worst adverse event that happens and then the HPE is resolved. All patients have a risk of death according to the health state they are in. Death can be a result of the adverse events or death as a consequence of age. 'Dead' is an absorbing state.

3.9. HEALTH STATES

Table 3.3. health states

Health states	
No adverse event	This is the point of model entry for either the HPE cohort (OAC+NSAID) or the non-HPE cohort (OAC only)
GI discomfort (27, 74)	People experience GI discomfort (diarrhoea, abdominal pain, dyspepsia, nausea, vomiting, flatulence), requiring treatment by a primary care physician.
Symptomatic ulcer (27, 74)	People have a clinical diagnosis of a peptic ulcer following adverse GI symptoms, which may include endoscopic confirmation.
Serious GI event	People experience a serious Gi event (GI bleeding, peptic ulcer bleeding or GI ulcer perforation) requiring treatment in hospital.
Post-event	Following an alarming GI event, people return to a health state where the risk of subsequent events is the same as in the non HPE cohort. From here they can experience further events
Stroke (27, 74)	People experience stroke requiring hospitalisation.
Post stroke	Following an alarming cardiovascular event, people return to a health state where the risk of subsequent events is the same as in the non HPE cohort. From here they can experience only further cardio-vascular events or die.
Dead	There is a risk of death from all health states

3.10. COHORT CHARACTERISTICS

Cohort characteristics were selected to reflect as closely as possible the target population in the UK. Results from the epidemiological study conducted as part of LP's PhD on patient characteristics were used to define the cohort characteristics in this model. This study was described in detail in Chapter 4 and will be referred to as the cohort study conducted as part of this PhD. The baseline characteristics of the cohort were representative of patients who were prescribed an OAC in primary care. The mean age of 70 years in the CPRD/HES/ONS cohort study

was used as the age at model entry. The proportion of men (55.11%) and women (44.89%) was also used from the primary care records.

3.11. TRANSITION PROBABILITIES

In this section the transition probabilities used to populate the state transition model are described. A summary of all input parameters is provided in Table 6.1.

3.11.1. Probability of GI discomfort in the absence of an NSAID

In the phase 3 trials of directly-acting oral anticoagulants (DOACs) compared with warfarin, ARISTOTLE, ROCKET-AF and Engage AF TIMI 48, the risk of GI discomfort was not increased in patients with the DOAC under investigation compared with warfarin, and therefore the incidence of GI discomfort was not reported. The only DOAC that was found to have an impact on dyspepsia in particular was dabigatran (127). Due to this increased risk, results on the effect on GI discomfort were reported in more detail for the RE-LY trial comparing dabigatran and warfarin. A combined outcome including upper abdominal pain, abdominal pain, abdominal discomfort and dyspepsia was reported for the dabigatran and warfarin cohort. Because dabigatran is rarely used in the UK (128) and the other RCTs in AF found no difference in the probability of GI discomfort between warfarin and the DOAC, the probability for warfarin was assumed to be representative of the OAC cohort. The mean age in the RE-LY trial was 71, which is very close to the age at cohort entry in this state-transition model. In the warfarin cohort, the incidence was reported to be 348 cases out of 6,022 participants with a median follow-up of 2 years, equivalent to a rate of 2.9 (95% CI 2.6 to 3.2) per 100 person years(127). This probability was used to derive a three-months probability for GI discomfort. Risk of GI discomfort was assumed to be constant over time for warfarin and dabigatran users as reported in a Danish observational study (129).

3.11.2. Probability of death from GI discomfort

Following consultation with the GPs, it was assumed that subsequent to a primary-care managed episode of GI discomfort there would be no increase in the risk of death. Therefore, age-adjusted general population mortality rates, according to the Office for National Statistics (29), were applied to this health state. Other decision analytic models, such as by Dorian et al. (2014) (96) or Lip et al. (2012, 2014) (101, 130) also used a constant probability of dyspepsia with no fatal events.

3.11.3. Probability of recurring GI discomfort in subsequent cycle

No literature was identified regarding the short-term recurrence/resolution of GI discomfort. Following consultation with clinicians, it is assumed that once somebody enters the GI discomfort state, their risk of recurring dyspepsia for the following cycle is the same as the risk of recurring serious GI events for which there is some observational data. After a maximum of two cycles of dyspepsia it is assumed that it is resolved, and the person enters the post-event state. Consultation with GPs confirmed this was a reasonable approach.

3.11.4. Probability of symptomatic ulcer in the absence of an NSAID

No evidence was found in epidemiological studies that OACs were associated with an increased risk of symptomatic ulcers compared with the general population (83, 131, 132). Clinically the mucosa is not affected by OACs and the RCTs did not report an increased risk of peptic ulcer. The probability of reported peptic ulcer was low in the ARISTOTLE trial with 0.01% of warfarin and 0.01% of apixaban patients and in the ROCKET-AF trial with 0.03% for rivaroxaban and 0% for warfarin according to results presented on clinicaltrials.gov as reported adverse events during the trials. The adverse events reported in the review of adverse events were not seen as representative as was not clear, if these self-reported events were complete. An observational cohort study published after the review using the UK THIN database analysed trends in

symptomatic ulcer incidence until 2005. High risk patients with bleeding or peptic ulcer (PU) history were excluded. The risk of uncomplicated peptic ulcer was 1.4 (95% CI: 1.3; 1.5) and 1.1 (95% CI: 1.0; 1.2) per 1000 person years for men and women, respectively (132). This rate for the age group 65-74 years of age by Cai et al. was used to calculate the 3 months peptic ulcer risk in the OAC cohort weighted by the proportion of female and male patients in the model population. For patients 85 years or older the estimate from Cai et al for the subsequent age group was used.

A similar incidence rate was reported in a systematic review including studies until February 2009. Incidence of uncomplicated peptic ulcer was 0.9 (95%CI: 0.78; 1.04) per 1000 PY with most studies dated before 2000 and only one from the UK (133).

3.11.5. Probability of death from symptomatic ulcer

An observational study collected data across a Danish county (population 470,000) between 1993 and 2002 (42). In the first month following an uncomplicated (i.e. non-bleeding) gastric ulcer, the standardised mortality rate (i.e. factor by which the expected number of deaths in the absence of an ulcer is multiplied to get the observed number of deaths) was 11.6 (95% CI 9.6-13.9) (42). A Finnish observational study collected data on 4154 patients with uncomplicated peptic ulcers between 2000 and 2008. The reported standardised mortality ratio was 2.16 (95% CI: 2.05; 2.29) at one year (43). In our base case, we used a weighted average of these numbers, assuming the higher one applied for the first 30 days and the lower for the remainder of the cycle – i.e. (11.6 × 30 + 2.16 × [91.3 – 30]) / 91.3, where 91.3 is the number of days in a 0.25-year cycle). This value was multiplied by the probability of death in the no adverse event state to estimate the increased probability of death in the first cycle post-ulcer (after which the probability of death is assumed to return to that in the general population).

3.11.6. Probability of recurring symptomatic ulcer

No study was found that assessed the risk of symptomatic ulcer recurrence. A Korean and a Finnish study were available that looked at any ulcer recurrence reporting a cumulative incidence over five and 1 year, respectively (134, 135). The study by Malmi et al. included all types of hospitalised peptic ulcers, such as perforated, bleeding, asymptomatic and symptomatic ulcers. The observational study collected data from hospitals in a district in Finland and reported a cumulative incidence of recurrent ulcers of 13.1% in the first year (95%CI: 12.4; 13.9)(135, 136). The observational study by Yoon et al. investigated recurrences of NSAID induced peptic ulcers and reported a five-year cumulative incidence of 10.9% (95% CI: 2.6% to 19.2%). However, sample sizes were small with only 57 NSAID related ulcers.

Both studies did not report a three months probability of the recurrence. Assuming a linear increase over time, a three months probability could be calculated, but Malmi et al. 2014 showed that the recurrence risk is highest in the first months. They also did not measure recurrence of symptomatic ulcer, but measured ulcers in hospital including bleeding events, which were considered serious GI events in this model. Instead of using these estimates, that were reported for a general population, the meta-analysed estimates in anticoagulated patients used for serious GI events, were considered more appropriate (137). Tapaskar et al. report a three-month probability of 10.1 %, that did not need to be transformed further. The two GPs involved in the clinical validation considered 10.1% a reasonable recurrence rate for symptomatic ulcer.

3.11.7. Probability of serious GI events in the absence of an NSAID

Incidence rates for serious GI events in patients with OAC treatment from a cohort analysis conducted in linked UK primary and secondary care data (CPRD/HES) were used to derive the three months probability of serious GI events.(113) The mean age of OAC patients was 72 years and the incidence rate was 6.87 (95% CI: 5.25; 9.00) serious GI events per 1000 person-years for

OAC users with no concurrent NSAID use. To adjust for the increasing risk of serious GI events with age, a risk adjustment factor was previously used in a NICE technology appraisal (117) and other models (97-100, 102-104, 138, 139). The risk adjustment factor was based on a systematic review on intracranial haemorrhage (140). However, more recent data from the ATRIA study, a cohort study using routinely collected health care data in the US, reported a two-fold increase of the bleeding risk for warfarin patients between 70 and 79 years of age compared to patients aged under 60 years, but only reported an adjusted RR of 2.1 (95% CI: 0.7; 6.4) for patients ≥80 years compared with patients aged under 60. The entry age of this cohort is already after the risk levelled off and therefore, no increased risk of serious GI bleeding events over time was applied.

3.11.8. Probability of deaths from serious GI events

No UK study was found that reported fatality of serious GI events in anticoagulated patients. Serious GI event rates reported in the cohort study in linked CPRD/HES/ONS were too small to show robust incidence rates of death from these.(113)

An observational study using Swedish routinely collected health care data assessed 90 day mortality of OAC users after 4291 hospitalisations for GI bleeding events (141). Of 652 DOAC patients with a serious GI event, 71 (10.9%) died within 90 days. For the 1293 warfarin users the 90-day mortality was 11.4% (n=147) and 10.9% for the 652 DOAC users. The mean age of the cohort was 78 years for warfarin and DOAC patients experiencing GI bleeding events. The incidence estimates for warfarin and DOACs reported in the study were combined (weighted by the number of patients in each group) and used as 3 months probability of death following the serious event in the age group 75-79 years. After validation from GPs, for other age groups the estimate was adjusted using the relative relationship to the according mortality in the general population (41).

3.11.9. Probability of recurring serious GI events

A systematic review and meta-analysis of studies published up to February 2019 assessing the risk of GI bleeding recurrence, thromboembolism and mortality in patients with a GI bleeding event conducted a meta-analysis of the 90 day re-bleeding probability (137). The identified studies investigated OAC users from the incident bleeding event taking the OAC for AF, deep vein thrombosis or pulmonary embolism. The mean age in the studies was 75 years. Among 1,912 patients that continue OAC therapy after the incident bleeding event, as assumed in this model, 192 experienced recurrent bleeding, for a 90-day probability of recurrence of 10.1% (95%CI 8.8% to 11.5%). A Danish observational study reported almost no change in the cumulative incidence of recurrent GI bleeding after 3 months (126). Therefore, people were permitted to remain in the serious GI event state for a maximum of one additional cycle, after which they move to the post-event state.

3.11.10. Probability of stroke in the absence of an NSAID

Incidence rates for stroke in anticoagulated patients reported in a cohort analysis conducted in linked UK primary and secondary care data (CPRD/HES) were used to derive the three-months probability of stroke(142). The incidence rate of strokes that lead to hospital admission in anticoagulated patients without NSAID treatment was 7.97 (95% CI: 6.21; 10.22) per 1000 person years for anticoagulated patients with no concurrent NSAID use and a mean age of 72 years. From this incidence rate, a three-months probability of 0.18% was generated and applied in the model. This estimate was considered reasonable because it was in line with estimates from the ARISTOTLE trial (143). From the one-year probability in ARISTOTLE a three-months probability of stroke of 0.24% for apixaban and 0.26 for warfarin was calculated.

The risk of stroke increased with age in patients with and without OACs and was adjusted for the age groups over 79 years (144). An analysis of the Atrial Fibrillation Investigators database with patient level data from 12 RCTs comparing treatments in AF reported a 1.45 (95% CI 1.26 to 1.66) increase in stroke risk with every ten year increase in age for patients on OAC, antiplatelet or placebo (145). The data suggested that the HRs of the effect of age were similar between OAC, antiplatelet and placebo treatment. Lip et al. (2015) also demonstrated similar trends with age in stroke and thromboembolism Kaplan-Meier curves for patients with and without OACs (144). As a result, it was assumed that estimates from an AF population including patients with OACs, antiplatelets and non OAC users were appropriate to estimate the risk increase due to age in a population on OAC treatment.

3.11.11. Probability of death from stroke

A study using Swedish routinely collected health care data investigated the 90-day mortality after 6017 strokes. For warfarin and DOACs 296 patients died resulting in a 90-day mortality of 17.6% (141). Compared with trial estimates from ARISTOTLE this estimate seemed reasonable. The one-year probability of death after a stroke was found to be 30.1%, with over half of these deaths within 30 days after the event (143). The mean age of the Swedish stroke cohort fell in the range 80–84; for other age groups we adjusted the estimate using the relative relationship to the according mortality in the general population (41).

3.11.12. Probability of recurring stroke

The probability of experiencing a second stroke in the subsequent cycle after a stroke event was highest in the first three months after the incident event (146, 147). Data from the South London Stroke Registry, a register of all first stroke incidences in a defined population in London, reported cumulative mortality rates from Kaplan-Meier estimates. The three-months probability of a recurrent stroke was 2.1% (CI 95% 1.3% to 3.4%) (148).

A multivariate analysis found an increased risk of stroke recurrences with every ten years of age (HR 1.16, 95% CI 0.98 to 1.37) (146). This HR was applied to the probability of recurring stroke for the age groups over 79 years in the sensitivity analysis.

3.11.13. Probability of stroke in the post stroke state

The probability of experiencing a stroke event in secondary prevention of stroke, hence the probability of transitioning from the post stroke state to the stroke state was investigated in sub-group analyses of the RCTs comparing DOACs and warfarin according to a systematic review (149). The three most common OACs in the UK are apixaban, rivaroxaban and warfarin, which were compared in the ROCKET-AF and ARISTOTLE trial. The subgroup analyses of patients with a history of stroke or TIA identified annual rates of recurrent stroke of 2.66 (95%CI 2.28 to 3.09) and 2.71 (95%CI 2.32 to 3.15) for rivaroxaban and warfarin, respectively (150), and 2.26 (95%CI 1.75 to 2.87) and 3.17 (95%CI 2.57 to 3.87) for apixaban and warfarin (151). A weighted average weighted by the number of patients with a previous stroke in each study was used to generate the three months probability of experiencing a stroke in the post stroke health state.

3.11.14. Probability of death in the post stroke state

A subgroup analysis of the ARISTOTLE trial investigated the impact on death of the existence of a previous stroke or TIA in patients with warfarin and apixaban compared with patients without a previous stroke or TIA (151). Previous stroke or TIA was associated with an increased risk of death with a HR of 1.27 (95% CI: 1.11; 1.45). This study was seen as representative of the post-stroke population, because it represented two of the three most common OACs in the UK (128).

3.11.15. Probability of death from the "No adverse event" or post GI event state

OAC use is associated with a reduced mortality rate compared with non-use in an AF population (152). A meta-analysis of AF trials identified a mortality rate of 46.3 (CI 95%: 39.9–53.2) per 1000 person years (153). Age varied with a mean or median age of 70 to 73 years between the trials. Age and a predominance of men were similar to the model cohort characteristics. Because patients age with time while they move through the model, the probability of death was adjusted with age. The probability of death for other age groups was estimated from the identified base value according to the relative risk of death by age in the general population. The death probabilities in the general population by age group that were used as reference were derived from the Office for National Statistics (41). The probability of death from each health state dependent on age groups in the model is summarised in
Table 2.4.

Table 2.*4*: Age-specific 3-month probability of death for each health state. In bold is the base value identified in the literature. The probability of death for other age groups was estimated from this value based on the relative risk of death by age in general population according to the Office for National Statistics

**Health state**	**Age group (years)**				**Source of base value[a]**
	70–74	75–79	80-84	85-89	
No adverse event/ Post-GI event	**1.15%**	2.02%	3.55%	6.51%	Gomez-Outes (2016) (153)
Serious GI event	6.34%	**11.21%**	19.81%	36.93%	Komen (2019) (141)
GI discomfort	**1.15%**	2.03%	3.56%	6.58%	Gomez-Outes (2016) (153)
Symptomatic ulcer	**5.91%**	10.21%	17.38%	30.11%	Lassen (2006) (42)
Stroke	5.64%	9.95%	**17.59%**	32.78%	Komen (2019) (141)
Post-stroke	**1.46%**	2.56%	4.50%	8.28%	Easton 2012 (151)
[a]Base value is the value identified in the literature for the respective age group. The base value is in bold letters. The probability of death for other age groups was estimated from this value based on the relative risk of death by age in general population as per Office for National Statistics					

3.11.16. Impact of absence/presence of NSAIDs on the probability of adverse events

This section reports the probabilities affected by the presence of NSAIDs in patients with OACs. The probability of these ADEs in the absence of NSAIDs was outlined before. The key feature of the HPE and the non-HPE cohort was that the transition probabilities only differed in the no adverse event health state. All other transition probabilities were the same. Hence, the probabilities affected by the presence of the NSAID are those from no adverse event to any of the ADEs (stroke, serious GI events, symptomatic ulcer, GI discomfort). Risk ratios were used to reflect the effect of NSAIDs on the likelihood of the ADEs in the model and are summarised in Table 3.5.

Table 3.5: Summary of impact of the presence of the NSAID on transition probabilities

Description	**Risk ratio (95% CI)**	**Distribution[a]**	**Reference**

Increased likelihood of serious GI event	HR 3.01 (1.63; 5.55)	Lognormal	(142)
Increased likelihood of GI discomfort	OR 2.12 (1.73; 2.58)	Lognormal	(154)
Increased likelihood of symptomatic ulcer	OR 1.70 (1.49;1.94)	Lognormal	(155)
Increased likelihood of stroke	HR 2.71 (1.48; 4.96)	Lognormal	(142)
[a]Distribution for probabilistic analysis; GI: gastro-intestinal; HR: hazard ratio; NSAID: non-steroidal anti-inflammatory drug; OR: odds ratio			

The impact of NSAIDs on death was assumed to be captured by the fatal events in the adverse event health states. As a result, the probability of transitioning from the no adverse event state to the dead state was the same in the HPE and non-HPE cohort. From the cohort study in CPRD/HES/ONS hazard ratios were derived for the increased risk of serious GI events and stroke events (142). The cohort study only investigated events recorded in secondary care. Risk ratios for the increased likelihood of symptomatic ulcer and GI discomfort were defined as adverse events managed in primary care in the model specifications. Consequently, the risk ratios need to be derived from other sources.

The impact of NSAIDs on the risk of symptomatic ulcer was investigated among other risk factors in a case-control study using the UK THIN database (155) that is broadly representative of the UK population (156). For 3914 cases of uncomplicated peptic ulcer disease and 9969 controls, an OR of 1.70 (95% CI 1.49 to 1.94) was reported for current NSAID use. The increased risk of GI discomfort with NSAIDs was investigated in a case-control study using health records from the UK QRESEARCH database (154). In the study period from 2000 to 2004, uncomplicated GI adverse events were more likely in patients with NSAID use. The risk of GI discomfort with different NSAIDs varies. The risk ratio of the most common NSAID, with 59% of prescriptions in the UK, was used to represent the general population in the model (26). The OR of GI discomfort for naproxen compared to no naproxen use was 2.12 (95% CI 1.73 to 2.58) (154) and was used in the base case analysis. This study represented a UK population and has been used for the transition probabilities to GI discomfort/dyspepsia in a previous economic model that informed national guidelines in the management of osteoarthritis (120).

3.12. COSTS

It was assumed that people in all health states (other than dead) in the HPE and the non-HPE cohort would receive their standard OAC treatment, which was not included in the health state costs. Temporary discontinuations of OACs after serious GI or stroke events were considered negligible and were not included.

3.12.1. No adverse event (HPE and non-HPE)

In the no adverse event state and the post-GI event state, no cost other than the resource use required for the hazardous prescription or the alternative treatment are generated. After consultation with the GPs, and in accordance with other published state-transition models (70, 80), paracetamol was used as the alternative non-hazardous prescription. The cost of paracetamol for 90 days is included in the no adverse event state in the non-HPE cohort and in the post-GI event state. The cost of NSAIDs for 90 days are included in the no adverse event health state in the HPE cohort. NSAIDs were costed in the HPE cohort in the no adverse event

health state until the HPE is resolved. Hence, once the HPE is resolved the same resource use applies in the HPE and the non-HPE cohort in the no-adverse event state.

The resource use of treatment with the two drugs was generated as follows: The most commonly prescribed NSAID in England was naproxen with 59% of all oral NSAID prescriptions (26) and most frequently dispensed as 500mg tablets (157). The defined daily dose was 500mg, resulting in one naproxen tablet per day (158). Paracetamol was most commonly dispensed as 500 mg tablets (157) and its defined daily dose is 3000mg. The daily dose paracetamol was set to six tablets per day.

3.12.2. *Resource use associated with GI discomfort*

Previous work by Elliott et al. provided relevant disaggregated resource use data for GI discomfort (52). However, this paper was from 2006. Therefore, expert opinion was sought from two GPs to confirm whether this still reflects current practice. Resource use included cost of treatment according to UK guidelines and cost of 1 GP visit. The UK guideline recommendations for un-investigated dyspepsia include a full dose PPI for four weeks once per day (159). Of the full dose PPIs listed in appendix A of the guideline omeprazole 20mg was the most frequently dispensed PPI in England as of June 2020 (157).

3.12.3. *Resource use associated with symptomatic ulcer*

For symptomatic ulcer Elliott et al. (2006) reported resource use in the NHS (52). To reflect current practice, GPs were consulted to adjust the reported resource use to the current care practice. The updated resource use included one diagnostic endoscopy, two GP visits, one out-patient visit, a H. pylori test and prescriptions of PPI. For peptic ulcer treatment UK treatment guidelines recommend treatment of a full dose PPI for eight weeks (159).

3.12.4. *Resource use associated with serious GI events*

Healthcare costs in the serious GI event state were derived from a multi-centre, randomised trial assessing resource use of GI bleeding events in six UK hospitals (160). The study reported resource use of the initial admission to hospital and 28 days after discharge. It was the only study that reported post-discharge costs in addition to in-patient hospital resource use and included a comprehensive list of resource use items. The in-hospital admission incorporated resource use for intravenous fluids, lab tests, medication, fluids, blood component transfusion, endoscopies, surgery or radiological interventions to control bleeding, and adverse drug events. Resource use for post-discharge care for 28 days included re-admission, admission to nursing homes/residential care, accident and emergency department (A&E) visits, outpatient clinic visits, GP visits etc. Unit costs were attached to resource use from the cost year 2012/2013 using UK sources for reference costs. Inflated to 2020/21 cost a serious GI event generated £3,280.44 (standard error (SE) £259.08).

3.12.5. *Resource use associated with stroke and post-stroke*

The majority of the patients in the UK prescribed an OAC have a diagnosis of AF (161). Stroke severity and costs are higher in patients with AF than in non AF populations (162). Resource use of stroke wastherefore applied from an AF population. Luengo Fernandez et al. (2015) reported the cost of stroke events by event severity from patients in the Oxford Vascular study (OXVASC), a prospective cohort study of all vascular events in Oxfordshire, UK (163). The reported acute and long-term cost estimates were previously used in two NICE technology appraisals (57, 117). Resource use for the acute event was assessed for the in-hospital episode (diagnostic tests) and post discharge care for 90 days after stroke event (re-admission, emergency transport, A&E visits, outpatient clinic visits, admission to nursing homes/residential care, GP/ nurse visits etc). Resource use for long-term care after stroke include resources used for re-admission, inpatient

diagnostic tests, emergency transport, A&E visits, outpatient clinic visits, admission to nursing homes/ residential care and GP/ nurse visits. Costs were reported for the first 90 days of each event (acute event costs) and long-term costs were calculated as the mean annual excess cost of five years after the event.

The reported acute event costs were used to generate cost for the stroke health state and the excess long-term annual costs were used to generate costs in the post-stroke health state of this model. The annual cost were divided by four to generate three month costs. Costs were reported by stroke severity in Luengo Fernandez et al. (2013)(163). To generate input parameters for this study a weighted average based on the severity distribution of strokes in patients with apixaban and warfarin in the ARISTOTLE trial was calculated (101). The costs [cost year 2008/2009] were inflated to the cost year 2020/2021 using the Hospital & community health services (HCHS) pay and price index and the NHS cost Inflation Index (NHSCII) (164).

3.13. UTILITIES

3.13.1. Utilities associated with „No adverse event“/Post-GI event

The Euro heart survey collected data on quality of life in patients with AF, the most common diagnosis associated with the use of OACs, in 35 European countries from 2003 to 2004 (54). The study used the EQ-5D-3L questionnaire and results were translated into utilities using UK tariffs for the different dimensions. The Euro heart survey estimates have been used in other peer-reviewed models (55, 56) and a NICE technology appraisal (57, 58). Mean age of participants in the survey was slightly lower than the start age of this model with a mean age of 66 years. Percentage of men (60%) and women (40%) was very similar to the proportions in this model population. The majority of patients (68%) were prescribed OACs. A mean utility was reported of 0.779 (SD 0.253). This utility was used to calculate QALYs generated in the "No adverse event" health state in the HPE and non-HPE model and in the Post-GI event state. To account for quality of life decreasing with age, the utility was multiplied by the ratio of the utility for a given age range relative to a reference age (66–70 years), based on general population utilities estimate. This method to account for the decreasing utility with age, was also used in a HTA (57). The relative relationship between the general population estimate from the English household survey for the age group 66-70 years, and the utility estimate from Berg et al, was used to calculate age dependent utilities.

3.13.2. Utilities associated with GI discomfort

A UK-based RCT of people aged 18–65 presenting in primary care with dyspepsia reported a baseline utility of 0.744 (n=679). (165) The mean age of the sample is not reported in the paper; however, the authors suggest that an appropriate expectation of utility in the comparable general population would be 0.82. This is closely comparable with the general population utility values for the 55–60 age group (0.8222) reported by Ara and Brazier (2012). Using this estimate as our general population anchor we calculate an absolute decrement of 0.079 caused by dyspepsia. To corroborate this estimate, we note that a small study in Malaysia asked people with headaches to complete the EQ-5D-3L and then compared the derived utility values by whether or not people also had dyspepsia (166). The utility decrement for dyspepsia from this study is also 0.08. Additionally, a 2001 study included a time trade-off study and reported that the QALY loss associated with dyspeptic symptoms was 0.09 (59). Therefore, we use the estimate derived from the UK RCT as our input value.

3.13.3. Utilities associated with symptomatic ulcer

We calculated disutility for this state using EQ-5D-3L values from the Health Surveys for England for people with 'other digestive complaints (stomach, liver, pancreas, bile ducts, small intestine

duodenum, jejunum and ileum)' (53). The 666 respondents who reported experiencing complaints of this type had a mean EQ-5D-3L of 0.734 (95%CI: 0.707 to 0.760), whereas respondents of a similar age not affected by the health condition (n=620) reported a value of 0.836 (95%CI: 0.815 to 0.856). This equates to a decrement of −0.102 (95%CI: −0.136 to −0.068). For comparison, a 2001 study included a time trade-off study and reported that the QALY-loss associated with a peptic ulcer was 0.11 (59), and the previous PINCER model included a decrement of −0.13(23, 60).

3.13.4. Utilities associated with serious GI events

A recent systematic literature review of utility decrements associated with bleeding events in people taking dual antiplatelet therapy reported that for 'gastrointestinal bleeds' the QALY-losses reported in the literature identified ranged from 0.005 to 0.016 (61). Campbell et al reported utility values derived from EQ-5D-3L for a cohort of 936 participants who were admitted to one of 6 UK university hospitals with an upper GI bleed (50). The participants completed the EQ-5D 28 days after they were admitted. The mean age of the sample was 59.4 years; the general population mean utility value for the 55 to 60 group is 0.8222 (53). In the Campbell paper the utility value for those who survived to 28 days was 0.735 (SD 0.02). Using these values to calculate a decrement (i.e. 0.8222 − 0.735 = 0.09) would imply less impact on quality of life than the utility decrements we use for symptomatic ulcer and a similar level of disutility as for GI discomfort. We judged this to lack face-validity. For the previous PINCER model the authors estimated a greater utility decrement (0.18); however, this was based on values originating from an obsolete US instrument with unclear valuation methods (23) (60).

In our base case, we use an estimate from Doble et al. (2018). The authors elicited EQ-5D values from people with experience of DAPT – and, in some cases, experience of DAPT-related bleeding – using vignettes describing minor and major bleeding events (61). When using EQ-5D-3L, they found that a major bleed is associated with a decrement of −0.239 (95%CI: −0.384 to −0.0933). The authors also elicited participants' opinions about the likely length of impairment (their average guess was 45.38 days); however, we do not use these data, instead applying the disutility to a full, 3-month cycle of our model. We have multiple reasons for doing so: (i) the participants had no experiential basis on which to provide an estimate, and – unlike the vignette describing the severity of the imagined incident – no other support to guide their answer; (ii) the vignette describes a bleeding event that is somewhat less serious than the defined state in our model (it describes an event that requires some sort of medical advice and/or treatment, whereas we envisage hospital admission will be necessary in every case); (iii) we know from other evidence that disutility from GI bleeds last for longer than 45 days (e.g. Wang et al. (2017) (62)show persisting deficits at 12 months after a major GI bleed). Therefore, we judged that applying the decrement to a full cycle would represent an appropriate level of disutility for the type of event our model simulates.

3.13.5. Utilities associated with Stroke

The reduction in health-related quality of life due to stroke was assumed to be a constant absolute decrement relative to having no stroke. The OXVASC study collected utilities of all stroke events (167). From 2002-2007, EQ-5D-3L utilities were available for 445 patients with stroke and 381 controls, with a mean age of 75 years. Other OAC models used in HTAs submitted to NICE (57, 58) used utilities from a time trade-off study published in 2001. From 57 patients, utilities for mild and severe stroke were assessed showing highly skewed utilities. The state-transition model by Sterne et al. (2015) used the utility for severe stroke for all severities of strokes identified in their model (57). This overestimates the disutility of stroke neglecting the impact of the more common minor and moderate strokes (167). Edwards et al. (2011) applied

the utility for the different severity levels in their model submitted as part of an HTA (58). In our *de novo* state-transition model, the utility from the OXVASC study was used (167). This was the largest and most recent study and the only English study that used the preferred utility elicitation method. Both HTAs assumed that the disutility from Robinson et al. (2001) persisted over the cycle length of three months. The more recent data from the OXVASC study, however, clearly showed that the utility after one months was much higher than this (167). The reported utility at one month after the stroke were assumed to capture the average utility over the first three month after the event. The utility difference between stroke compared with the controls was of 0.22 (95% CI 0.18 to 0.26). This difference was used as the three-months decrement applied to the age dependent utility.

3.13.6. Utilities associated with the post-stroke state

The OXVASC study reported not only utilities at one months after the stroke event, but also long-term utilities (167). The study reported a utility difference between patients with a previous stroke and patients in the control group of 0.18 (95% CI: 0.13; 0.23; p < 0.001). Therefore, this recent, English utility difference estimated in the OXVASC study was used as the three months utility decrement for post-stroke.

3.13.7. Disutility of correcting HPE

Correction of this HPE involved the discontinuation of the NSAID and a switch to paracetamol therapy. According to the GPs, this was the preferred action [3]. It was assumed that this switch did not affect utilities. Alternative treatments were considered to have the same treatment effect (i.e., the same pain relief as NSAIDs). The same assumption was previously made in a HPE model involving NSAID use in the elderly (80). The patient representative highlighted that this might not be the case, because paracetamol has a slightly smaller effect on pain relief. However, this could not be included in the model, because no quantitative data exists on the disutility of paracetamol treatment compared with NSAID treatment.

4. Prescription of an oral NSAID to a patient with heart failure (Query I)

4.1. BACKGROUND

Heart failure (HF) is a clinical syndrome characterised by the inability of the heart to pump enough blood to meet the body's demands. Symptoms include dyspnoea and fatigue which may limit exercise tolerance as well as fluid retention which may lead to peripheral oedema and pulmonary congestion.

A recent review of economic evaluations in interventions to manage heart failure (168) noted that the majority of studies categorised the severity of heart failure according to criteria established by the New York Heart Association (NYHA) which are summarised in Table 4.1 (169).

Table 4.1. New York Heart Association (NYHA) heart failure descriptive system (169)

Class	Patient Symptoms
I	No limitation of physical activity. Ordinary physical activity does not cause undue fatigue, palpitation, dyspnoea (shortness of breath).
II	Slight limitation of physical activity. Comfortable at rest. Ordinary physical activity results in fatigue, palpitation, dyspnoea (shortness of breath).
III	Marked limitation of physical activity. Comfortable at rest. Less than ordinary activity causes fatigue, palpitation, or dyspnoea.
IV	Unable to carry on any physical activity without discomfort. Symptoms of heart failure at rest. If any physical activity is undertaken, discomfort increases.

4.2. HEART FAILURE AND NON-STEROIDAL ANTI-INFLAMMATORY DRUGS (NSAIDs)

Heart failure is a progressive disease, generally presenting in people over the age of 70, that is generally managed by lifestyle modification, guideline-directed medical therapy and/or implantable cardiac devices, and/or surgery. Progression of the disease leading to lack of control of symptoms means that regular treatment reviews are required. In addition to this, a range of factors can trigger symptoms of uncontrolled heart failure which can range from mild to life-threatening in people with previously well-managed HF. Consumption of NSAIDs has been associated with acute worsening of heart failure in people with pre-existing heart failure via fluid retention. The economic model needs to reflect the likely events that occur when people with heart failure experience an NSAID-induced worsening of heart failure.

4.3. THE MODEL

A recent review of economic evaluations related to heart failure found that the Markov approach was the most common (168). If possible, the structure of the economic model was planned to be developed from previously published relevant models that reflect the short and long-term consequences of an NSAID-induced acute worsening of heart failure in people with pre-existing heart failure. Initial scoping suggested that there were very few models that addressed this specific question. Therefore, the literature review was widened to include economic models that described heart failure events attributed to any cause. Model structure was further informed by consultation with experts in heart failure management.

4.4. LITERATURE SEARCH

The starting point of the literature search was an existing systematic review published in 2011(170). The search strategy in that review is shown in Table 4.2 (below).

Table 4.2. Search strategy employed in Goehler et al 2011 systematic review (170)

In MEDLINE/PreMEDLINE (1966 to June 2010), we used one of the following Medical Subject Heading (MeSH) terms: ‘Technology Assessment, Biomedical’, ‘Decision Support Techniques’, ‘Models, Statistical’, ‘Markov Chains’, ‘Monte Carlo Method’, ‘Economics’, ‘Health Planning’ or ‘Quality-Adjusted Life Years’, combined with the MeSH term ‘Heart Failure’.<br><br>In EMBASE Drugs and Pharmacology (1966 to June 2010), our keywords were ‘decision analysis’, ‘decision-analytic’, ‘decision model’, ‘health care model’, ‘health care evaluation model’, ‘decision tree’, ‘Markov model’, ‘cost-effectiveness’, ‘cost-utility’, ‘cost-benefit’, ‘cost-minimi[z/s]ation’ or ‘QALY’ in combination with the term ‘heart failure’.<br><br>In EconLit and the Cost-Effectiveness Analysis Registry, we searched for ‘Heart Failure’.

The databases and search terms used in the development of this model are reported in Table 4.3.

Table 4.3. Literature searches conducted

Searches	Search terms
Model structure and costs*	• Medline<br>economic evaluation OR economic analys* OR cost analys* OR cost effective* analys* OR cost-effective* analys* OR cost benefit* analys* OR cost utility* analys* OR cost-benefit* analys* OR cost-utility* analys*<br>AND<br>heart failure[MeSH Terms]<br>AND

	model[Title/Abstract]<br>• Scopus<br>economic AND evaluation OR economic AND analys* OR cost AND analys* OR cost AND effective* AND analys* OR cost-effective* AND analys* OR cost AND benefit* AND analys* OR cost AND utility* AND analys* OR cost-benefit* AND analys* OR cost-utility* AND analys*<br>AND<br>TITLE (heart AND failure)<br>AND<br>TITLE-ABS (model)<br>• Embase<br>((economic and evaluation) or (economic and analys*) or (cost and analys*) or (cost and effective* and analys*) or (cost-effective* and analys*) or (cost and benefit* and analys*) or (cost and utility* and analys*) or (cost-benefit* and analys*) or (cost-utility* and analys*)).af.<br>AND<br>(heart and failure).ti.<br>AND<br>(model).ab.<br>NHS EED/HTA databases: heart failure
Utilities	• Medline<br>EQ-5D*[Title/Abstract] OR SF-12[Title/Abstract] OR QALY*[Title/Abstract] OR quality-adjusted*[Title/Abstract]<br>AND<br>heart failure[MeSH Terms]))<br>• Scopus<br>ABS (eq-5d* OR sf-12 OR qaly* OR quality-adjusted*)<br>AND<br>TITLE (heart AND failure)<br>• Embase<br>((EQ-5D*) or (SF-12) or (QALY*) or (quality-adjusted*)).ab.<br>AND (heart failure).ti.
Risks (death)	• Medline<br>survival[Title] OR mortality[Title]<br>AND<br>heart failure[MeSH Terms]<br>AND<br>UK[Title/Abstract] OR Engl*[Title/Abstract]<br>• Scopus<br>TITLE (survival OR mortality)<br>AND<br>TITLE (heart AND failure)<br>AND<br>TITLE-ABS(UK OR Engl*)<br>• Embase<br>(survival OR mortality).ti.<br>AND<br>(heart failure).ti.<br>AND<br>(UK OR Engl*).ab.

Risks (impact of NSAIDs)	• Medline<br>NSAID[Title/Abstract] OR (nonsteroidal[Title/Abstract] AND anti-inflammatory[Title/Abstract]) OR (non-steroidal[Title/Abstract] AND anti-inflammatory[Title/Abstract]))<br>AND<br>heart failure[MeSH Terms]<br>• Scopus<br>TITLE-ABS(NSAID OR (nonsteroidal AND anti-inflammatory) OR (non-steroidal AND anti-inflammatory))<br>AND<br>TITLE (heart AND failure)<br>• Embase<br>(NSAID OR (nonsteroidal AND anti-inflammatory) OR (non-steroidal AND anti-inflammatory)).ab.<br>AND<br>(heart failure).ti.

*identified models also searched for potential utility and probability parameters; EED = economic evaluation database; HTA = health technology assessment

Titles and abstracts were screened and full texts of potentially relevant records used to identify potential model structures and parameters. Models focussed on heart transplant or left ventricular assist device (LVAD) implant procedures were excluded as these related exclusively to people with very severe/end-stage heart failure which was not the focus of this model. For model parameters, studies were considered preferable if they were large, UK-based/relevant, and recent (i.e. published since 2010). In March 2019 the searches for model parameters were re-ran to identify any new papers. A 2019 systematic review of economic models related to heart failure (168) was screened to ensure all relevant models were identified.

The published systematic reviews (168, 170) and subsequent literature searches identified 21 non-duplicated Markov models relating to outcomes in people with heart failure published between 2009 and 2019. The models ranged from two-state models (alive, dead) to more complex structures with 5 or more states. The review by Di Tanna et al noted that the majority of models that included hospitalisations classified them as 'general hospitalisations' rather than cause-specific (168). Only one model included 'minor events' (peripheral artery disease, transient ischaemic attack, and angina) (171). The most common time horizons were lifetime (7 studies) or 10 years (6 studies), the shortest time horizon was 4 years. The most common cycle lengths were 1 month (8 studies) or 1 year (8 studies).

4.5. EXPERT CONSULTATION

The mechanism of action whereby NSAIDs exacerbate heart failure (increased fluid retention leading to increased cardiac pre-load which may lead to cardiac decompensation) means that it is likely that this adverse event could either lead to a minor or major exacerbation of heart failure. However, there is little literature that clearly describes how NSAID administration to people with heart failure will manifest as an adverse event, so it was necessary to use expert opinion to help clarify this aspect of the model.

Model structure development was informed by the published literature and expert opinion. We convened a virtual panel of experts with experience of primary and secondary care management of heart failure (one patient safety pharmacist, and one pharmacist, GP, and cardiologist with expertise/special interests in heart failure). Key uncertainties remaining after the literature review centred on: 1) how would NSAID-induced exacerbations of heart failure manifest, and

would they differ from other exacerbations, and in primary or secondary care? 2) how would NSAID-induced exacerbations of heart failure be managed, and would they differ from other exacerbations? 3) would the NSAID continue to be prescribed after an episode of heart failure exacerbation, and would there be drop in quality of life (QOL) if it was not? 4) would NSAID-induced exacerbations of heart failure lead to permanent reduction in cardiac function?

The **key points** from the consultations were:

- NSAID-induced exacerbations of heart failure would resemble other exacerbations (e.g. an additional burden on a compromised system with a similar impact to a chest infection) and it is likely that NSAIDs would not be identified/recorded as a causative factor for the exacerbation at the time
- Heart failure exacerbations are managed in one of three ways, dependent on symptom severity and speed of onset of symptoms: self-managed (self-titration of diuretics), primary care, or secondary care and this would be the same for NSAID-induced exacerbations. Other than withdrawing the NSAID, management of NSAID-induced exacerbations of heart failure would not differ from other exacerbations caused by fluid overload
- There are not likely to be any long-term consequences of an NSAID-induced exacerbation of heart failure once the heart failure symptoms are back under control
- People with heart failure would not experience a loss in utility when NSAIDs are withdrawn as alternative analgesics or anti-inflammatories (e.g. paracetamol or prednisolone) would be prescribed

4.6. MODEL STRUCTURE

From literature review and expert panel input, the following model structure was developed.

Fig 4.1. Final model structure

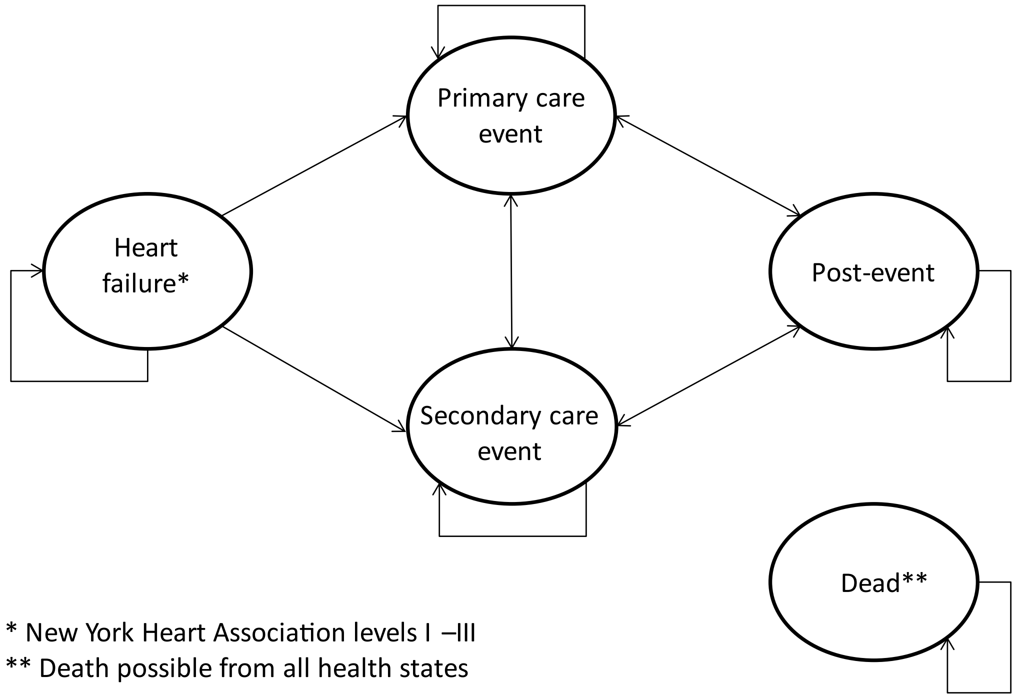

This structure reflects the fact that when people with stable heart failure ("heart failure') suffer an exacerbation, it can be sufficiently minor to allow primary care management ("primary care event"), or sufficiently serious to warrant secondary care admission ("secondary care event"). Mild worsening of heart failure symptoms that is self-managed is excluded from the model

because without a record of healthcare utilisation it is very difficult to identify the likelihood of this happening without studies that have specifically investigated this.

Recycling is possible within exacerbation event states (that is, a proportion of the population will spend consecutive cycles in the same exacerbation state). This is not to suggest that single exacerbation events lasting more than 3 months are likely; rather, it reflects the probability that a person will experience another event of the same type in the next 3-month period. Similarly, people may experience a secondary care event in the cycle following a primary care event and vice versa. People who experience an exacerbation that is not immediately followed by another return to their pre-exacerbation heart failure status in the following cycle ("post-event"). All people have a risk of death, according to the health state they are in. "Dead" is an absorbing state.

4.7. HEALTH STATES

The health states are summarised in Table 4.4.

Table 4.4. Health States

Health states	
Heart failure	People are assumed to enter the model with mild to moderate, controlled, heart failure (NYHA levels I-III)
Primary care event	People experience an acute, moderate, worsening of heart failure symptoms requiring primary care management
Secondary care event	People experience an acute, serious, worsening of heart failure symptoms requiring secondary care management
Post-event	Following a primary or secondary care event, people who do not experience a further event in the next cycle return to a health state characterised by controlled heart failure from where they can experience further events
Dead	There is a risk of death from all health states

In this model, the probability of a primary care or secondary care event is adjusted according to the presence or absence of NSAIDs, to allow calculation of the differential effect on outcomes and costs in a theoretical cohort. Once the event potentially triggered by NSAID prescribing has occurred, it is assumed that clinicians will take corrective action and cancel the NSAID prescription. Then the patient moves from the exacerbation health state to the "Post-event" health. Patients can still experience exacerbations from this health state, but the probability reverts to the "non-HPE" probability of exacerbations, i.e. the probability in the absence of an NSAID.

4.8. COHORT CHARACTERISTICS

The cohort is assumed to be aged 65 years at model entry because older adults (65+ years) with heart failure are particularly at risk of being prescribed NSAIDs due to a high frequency of comorbid conditions (172). The cohort is assumed to have mild to moderate heart failure (which corresponds to NYHA levels I-III) as discussion with experts suggested these groups are most likely to be prescribed NSAIDs i.e. as opposed to people with severe (NYHA level IV) heart failure.

4.9. TRANSITION PROBABILITIES

The transition probabilities used in the model are described in this section and summarised in Table 6.1. Additional searches (also described in Table 4.2) were conducted in relation to mortality and the impact of NSAIDs.

4.9.1. *Probability of primary care event in the absence of an NSAID*

Only one study was identified that reported use of primary care services in people with heart failure. The study reported data from UK electronic medical records (CPRD, accessed in 2015) to identify mean annual use of primary care services in people with a diagnostic code for heart failure (173). The probability of an episode of heart failure exacerbation requiring primary care (primary care event) in a person with heart failure was taken from this study. The mean annual use of emergency GP visits (0.14) and GP home visits (1.23) were used as a proxy for the probability of a primary care event (i.e. 1-year probability of primary care event = 0.14+1.23 = 1.37) so that routine check-up visits were excluded. The 1-year probability was converted to a 3-month probability (0.290) which was used in the model.

4.9.2. *Probability of secondary care event in the absence of an NSAID*

The probability of an episode of heart failure exacerbation requiring secondary care (secondary care event) in a person with heart failure was taken from a study which used UK routinely collected data (CPRD–HES–ONS linked data) from April 2005 to April 2014 to estimate the monthly hazard rate of all-cause hospitalisation in people with heart failure (174). This source was chosen because it is a very large sample (n=52,122) of people with heart failure in the UK. The monthly rate of all-cause hospitalisation for people aged <75 years reported in the source study was 0.066 (SE 0.008) (174), which was converted to a 3-month probability (0.180) for use in this model.

4.9.3. *Probability of death in the absence of an NSAID*

A UK study used a large database of routinely collected electronic primary care records (The Health Improvement Network (THIN)) between 1 January 1998 and 31 December 2012 to identify and follow people age 45 or over with heart failure from diagnosis until death (n=54,313) (175). There were no exclusion criteria based on the severity of heart failure and no measure of this was reported therefore it is unclear how well-matched this sample is with the model cohort. However, as the study sample were identified at diagnosis of heart failure it is unlikely that a large proportion would have severe symptoms (NYHA level IV) at that stage. The study reported survival rates at 1, 5, and 10 years post-diagnosis by age group (10-year groups) (175). The duration of heart failure in the model cohort includes those with recent onset and longer symptom duration therefore 5-year survival rates were used to estimate the likelihood of death in the model by age group [survival - 55-64yrs: 0.750 (95% CI 0.736, 0.764); 65-74yrs: 0.645 (95% CI 0.634, 0.654); 75-84yrs: 0.495 (95% CI 0.487, 0.503); 85-94yrs: 0.285 (95% CI 0.275, 0.296)]. The five year survival data was converted to a 3-month probability of death for use in the model. This source was chosen because it is a very large sample of people with heart failure in the UK.

The mortality rate was assumed unchanged following a primary care event. However a higher likelihood of death was assumed in the first cycle following a secondary care event. The National Institute for Cardiovascular Outcomes Research (NICOR) publishes annual statistics on outcomes of people admitted with acute heart failure to hospitals in England and Wales. Earlier publications contained age-stratified mortality data but the most up-to-date publication provides less detail. Therefore, we took the most recent audit with age-stratified results (2012–13)(176) and assumed that the relationship between in-hospital deaths in that period compared with the most recent report (2018–19)(177) is constant across all age-groups, applying the overall ratio to the age-stratified results to estimate present-day values. Because these data represent the probability of dying within 30 days of an acute admission, we added 2 months' background mortality to arrive at a 3-month cycle probability of death.

4.9.4. *Impact of NSAIDs on the probability of a primary or secondary care event, or death*

The literature search identified a number of studies on the relationship between NSAIDs and heart failure outcomes but the majority focus on incident heart failure while on NSAIDs which is not the question being addressed with this model. Two studies (Gislason et al (178) and Girouard et al (172)) were identified which used robust routine datasets and appropriate methodology to isolate the impact of NSAIDs in people with heart failure. Neither of these studies were conducted in the UK therefore the samples contributing data may be different from the cohort in this model. Table 4.5 shows the sample characteristics of these two studies and the UK samples reported in Taylor et al (175) and Pufulete et al (174) analyses of people with heart failure in large primary care databases.

Table 4.5. Comparison of sample characteristics in source studies

	Taylor et al (175)	Pufulete et al (174)	Gislason et al (178)	Girouard et al (172)
Country	UK	UK	Denmark	Canada
Sample size	54,313	17,095	107,092	1,112,491
Age	Mean not reported<br>64.8% >75yrs	Median age at incidence 77.9<br>60.4% >75yrs	Mean age of sample<br>74.8	Mean age of sample<br>78.7
% male	51.5%	56.1%	51.7%	46.1%
Inclusion criteria	Patients age 45 or over with a first diagnostic label of heart failure	Adults with a HF diagnosis* between 1 April 2005 and 31 March 2013.<br>*In CPRD: specific HF diagnosis codes only AND the presence of a BNP test or echocardiography test within 6 months of the HF code AND more than one HF medication prescription within 6 months of HF code.	Patients aged 30 years or older who between January 1, 1995, and December 31, 2004, survived their first hospitalization because of HF.	Patients >65 with at least 1 registered HF diagnosis in the outpatient medical visits database or the hospitalizations' registry from January 1, 2000, to December 31, 2009
	% of sample with comorbid condition			
Diabetes	19.8%	4%	17.7%	30.1%
Hypertension	53.1%	not reported	not reported	57.6%
Myocardial infarction	22.6%	12.1%	13.7%	not reported
Ischaemic heart disease	42.9%	16.7%	not reported	59.6%
Stroke	9.2%	1.8%	not reported	13.1%
Atrial fibrillation	28.5%	19.6%	not reported	27.7%

BNP: B-type natriuretic peptide: CPRD: Clinical Practice Research Datalink; HF: Heart failure

Both UK samples included patients with similar ages and there was a similar proportion of males. The incidence of comorbid conditions in the Pufulete (174) sample appear somewhat lower than the Taylor (175) sample. Compared with the UK samples, the Danish sample (Gislason (178)) was similar in terms of age and gender. The prevalence of comorbid diabetes and myocardial infarction in the Danish sample were both within the range from the two UK samples. There were a few more differences between the UK and the Canadian (Girouard (172)) samples - there were around 5-10% fewer men in the Canadian sample, and higher rates of diabetes and ischaemic heart disease. The samples were similar in terms of age and comorbid hypertension, stroke, and atrial fibrillation. Generally the Canadian sample had more comorbidities than the other samples, this may reflect that this sample was restricted to older adults only (even though the mean age was similar). In summary although there are some differences, they are similar enough that parameters derived from them are applicable to the cohort in our model.

4.9.5. NSAIDs and secondary care events in people with heart failure

The model parameter relating to the impact of NSAIDs on the likelihood of someone with heart failure being admitted to hospital was taken from a study of the electronic secondary care records for the province of Quebec in Canada between January 2000 and December 2009 (n=1,112,491) (172). The study was restricted to people over the age of 65 with confirmed (i.e. coded more than once in medical records) incident heart failure during the study period. During the study period 101,273 people were hospitalised. The odds ratio for being hospitalised (for any cause) following a prescription for NSAIDs was 1.16 (95% CI 1.13–1.20) compared with those who were not prescribed NSAIDs.

Although Gislason et al (178) also report on the relationship between NSAIDs and hospitalisations it was not used in the model. Gislason reports only heart-failure specific admissions whereas the health state in the model includes all hospitalisations. The experts consulted when developing this model believed that it may not always be identified that heart failure is the 'cause' of the hospitalisation when reason for admission is being recorded. Therefore, only looking at heart-failure specific admissions would underestimate the actual rate of admissions. Having said this the risk ratios reported in the two studies were remarkably similar (Girouard: 1.16 (172) and Gislason: 1.18 (178)).

4.9.6. NSAIDs and primary care events in people with heart failure

No data were found that specifically estimated the impact of NSAIDs on primary care events in people with heart failure. It was assumed that the relative increase in likelihood of a primary care event in the presence of NSAIDs was the same as the increased likelihood of a secondary care event in the presence of NSAIDs (i.e. odds ratio of 1.16) (172).

4.9.7. NSAIDs and mortality in people with heart failure

It is possible that NSAIDs could increase mortality only indirectly by their effect on the probability of a secondary care event. It is also possible that there could be an additional direct effect on mortality, through an additional lethal effect of the drug over and above the increased risk of an event, i.e. an increased risk of death once the event has occurred. In our base case, we assumed that observed excess mortality is additional, but we explored the impact of this assumption by removing it in sensitivity analysis. We also examined the excess deaths predicted by the model under both settings, to see if predicted deaths arising from secondary care events could explain the observed difference in overall deaths.

The impact of NSAIDs on the likelihood of death in people with heart failure was identified from a Danish study by Gislason et al (178) of linked administrative data for prescriptions, hospitalisations, and deaths. The study included patients aged 30 years or older who, between

January 1995 and December 2004, survived their first HF hospitalisation (n=107,092). Almost 60% of the sample died during follow-up (60,974; 56.9%). The paper reported a hazard ratio for the risk of death in the presence and absence of a number of different NSAIDs. In our base case, we used the hazard ratio for naproxen as it is by far the most common NSAID prescribed in England (178) (179). Table 4.6 shows the hazard ratios for naproxen and other NSAIDs as well as prescribing proportions. In a sensitivity analysis, we use an average of the hazard ratios, weighted according to the prescribing proportions, to estimate the impact of NSAIDs on risk of death.

Table 4.6. Total number of prescriptions for NSAIDs in all practices in England in January 2019 and hazard ratio for risk of death in people with heart failure compared with people not exposed to any NSAIDs

NSAID	Total number of prescriptions	% of total	Hazard ratio (95% CI) – risk of death versus no NSAIDs (178)
celecoxib	26,533	3%	1.75 (1.63-1.88)
diclofenac	63,892	8%	2.08 (1.95-2.21)
ibuprofen	184,623	23%	1.31 (1.25-1.37)
naproxen	533,821	66%	1.22 (1.07-1.39)

Data from: English Prescribing Dataset via Open Prescribing (179)

The ratios used in the model to reflect the presence of NSAIDs on the likelihood of the events in the model are summarised in Table 4.7.

Table 4.7. Impact of NSAIDs on transition probabilities

Description	OR/HR/RR (95% CI)	Reference
Increased likelihood of primary care events	1.16 (95% CI 1.13–1.20)	Girouard et al (172)
Increased likelihood of secondary care events	1.16 (95% CI 1.13–1.20)	Girouard et al (172)
Increased likelihood of death	1.22 (95% CI 1.07-1.39)	Gislason et al (178)

OR: odds ratio; HR: hazard ratio; RR: relative risk

The transition probabilities used in the model to represent the impact of NSAIDs in people with heart failure are summarised in Table 6.1.

4.9.8. Detection and correction of NSAID prescription

It was assumed that after a primary or secondary care event the HPE (i.e. the NSAID prescription) would be identified and withdrawn. Therefore following either type of event the transition probabilities were the same as in the absence of NSAIDs.

4.10. COSTS

It was assumed that people in all health states (other than dead) would receive their standard treatment/monitoring for heart failure therefore no costs were included for this.

In the heart failure state and the post-event state, no cost other than the resource use required for the hazardous prescription or the alternative treatment are generated. Paracetamol was used as the alternative non-hazardous prescription. The cost of paracetamol for 90 days is included in the no adverse event state in the non-HPE cohort and in the post-GI event state. The costs of NSAIDs for 90 days are included in the no adverse event health state in the HPE cohort.

The studies identified through the literature searches regarding model structure and parameters were also screened as possible sources of resource use associated with the events in the model. Observational data for the preferred model specification were not identified. The costs included in the model are summarised in Table 6.2.

4.11. UTILITIES

Electronic databases were searched to identify utility values for each health state in the model. There were no recent, robust UK studies that reported utility values derived from the EQ-5D or other directly measured or derived generic health status/utility measures which could be linked to primary or secondary care events. Therefore we identified sources which reported utility by NYHA level. Following discussion with experts it was agreed that a primary care event could be assumed to be equivalent to a short-term (i.e. one cycle length of the model) 1-class worsening of heart failure (i.e. level I to level II, or level II to level III) and a secondary care event equivalent to a 2-level worsening of heart failure. For the heart failure and post-event health states a mean utility value across NYHA levels I-III (i.e. corresponding to the model cohort) was assumed.

Although there was one UK-based study which reported utility values by NHYA class (180) that study used regression modelling to derive estimated utility values for NYHA levels I-IV (rather than directly collecting EQ-5D data from people with all levels of heart failure). Furthermore the regression-based estimates were from a cohort with more severe heart failure (NYHA II-IV) than the cohort in the model therefore this was not used in the model. An alternative study was identified which recruited people with heart failure in Australia, using the UK health state index for the EQ-5D-3L to generate utility values (181). The study included a sample of 598 people with predominantly mild-to-moderate heart failure (97% of the sample were NYHA classes I-III). The utility values derived from this study and included in the model are summarised in Table 6.2.

4.11.1. Disutility of correcting HPE

As described above, consultation with clinical experts concluded that people with heart failure would not typically experience a loss in utility when NSAIDs are withdrawn as alternative analgesics or anti-inflammatories (e.g. paracetamol or prednisolone) would be prescribed.

5. Prescription of an oral NSAID to a patient with chronic kidney disease with an eGFR <45ml/min (Query K)

5.1. BACKGROUND

Consumption of NSAIDs has been associated with acute kidney injury (AKI) in people with pre-existing chronic kidney disease (CKD). The grades of CKD, as defined by estimated glomerular filtration rate (eGFR) are summarised as:

- eGFR ≥60 ml/min: no or mild CKD
- eGFR 45-59.9 ml/min: stage 3a CKD
- eGFR 30-44.9 ml/min: stage 3b CKD
- eGFR 15-29.9 ml/min: stage 4 CKD
- eGFR <15 ml/min: stage 5 CKD.

5.2. DEVELOPING THE MODEL

For this model, individuals in the model cohort are at high risk of AKI, due to pre-existing CKD, and are either prescribed NSAIDs (HPE) or not (non-HPE). If possible, the structure of the economic model was planned to be developed from previously published relevant models that reflect the short and long-term consequences of an NSAID-induced episode of AKI in people with pre-existing CKD. Initial scoping suggested that there were very few models that addressed this specific question. Therefore, the literature review was widened to include economic models that

described AKI attributed to any cause. Model structure was further informed by consultation with experts in AKI management.

5.3. LITERATURE SEARCH

The databases and search terms used in the development of this model are reported in Table 5.1.

Table 5.1. Literature searches conducted

Searches	Search terms
Existing models (including costs and utility values)	Medline, Embase, Scopus<br>1. Acute kidney injury<br>2. Acute renal failure<br>3. Renal insufficiency<br>4. Acute kidney tubular necrosis<br>5. acute kidney injur$<br>6. acute renal injur$<br>7. acute kidney insufficienc$<br>8. acute renal insufficienc$<br>9. acute tubular necrosis<br>10. 1 or 2 or 3 or 4 or 5 or 6 or 7 or 8 or 9<br>11. kidney diseases<br>12. CKD<br>13. Renal failure<br>14. Kidney failure<br>15. Kidney failure, chronic<br>16. Renal insufficiency, chronic<br>17. 11 or 12 or 13 or 14 or 15 or 16<br>18. 10 and 17<br>19. Models, economic/ or models, econometric/<br>20. (Cost-Benefit Analysis or Cost Analysis or Cost Effective or Cost Utility).<br>21. Markov chain<br>22. Decision trees<br>23. Decision support techniques<br>24. Microsimulat*<br>25. (patient level adj8 simulat*)<br>26. (simulat* adj3 model*). and decision*.<br>27. (discrete event* adj5 simulat*).<br>28. (discrete event* adj8 model*)<br>29. (decision* adj5 model*)<br>30. (model* adj5 markov*)<br>31. (econom* or cost or costs) adj6 model*<br>32. state transition model<br>33. (transition probabilit* and (state or states or model*))<br>34. 19 or 20 or 21 or 22 or 23 or 24 or 25 or 26 or 27 or 28 or 29 or 30 or 31 or 32 or 33<br>35. 18 and 34<br>36. remove duplicates from 35<br>37. limit 36 to "all adult (19 plus years)"<br>HTA database: acute kidney injury OR chronic kidney disease
Risks (impact of NSAIDs)	Medline, Embase, Scopus:<br>1. NSAID<br>2. Nonsteroidal<br>3. Anti-inflammatory<br>4. Non-steroidal<br>5. CKD<br>6. Chronic kidney disease<br>7. 2 and 3

	8. 3 and 4<br>9. 1 or 7 or 8<br>10. 5 or 6<br>11. 9 and 10
Primary care management of AKI	Medline, Embase, Scopus:<br>1. Primary<br>2. Healthcare<br>3. Care<br>4. Practice$<br>5. Family<br>6. General<br>7. Community<br>8. AKI<br>9. Acute kidney injury<br>10. 8 or 9<br>11. Management<br>12. Treatment<br>13. 11 or 12<br>14. 2 or 3 or 4<br>15. 1 and 14<br>16. 5 and 14<br>17. 6 and 14<br>18. 7 and 14<br>19. 10 and 13 and 18
Utility values	Medline, Embase, Scopus:<br>1. acute kidney injury<br>2. acute renal failure<br>3. renal insufficiency<br>4. acute kidney tubular necrosis<br>5. acute kidney injur$<br>6. acute renal injur$<br>7. acute kidney insufficienc$<br>8. acute renal insufficienc$<br>9. acute tubular necrosis<br>10. 1 or 2 or 3 or 4 or 5 or 6 or 7 or 8 or 9<br>11. Kidney disease<br>12. CKD<br>13. Renal failure<br>14. Kidney failure<br>15. Kidney failure, chronic<br>16. Renal insufficiency, chronic<br>17. 11 or 12 or 13 or 14 or 15 or 16<br>18. 10 and 17<br>19. EQ5D<br>20. EurQoL 5d<br>21. EQ-5D$<br>22. AQOL<br>23. Quality Adjusted Life Year$<br>24. Quality Adjusted Life Year*<br>25. SF-36<br>26. SF6D

	27. SF12
	28. ADL Questionnaire
	29. HUI Questionnaire
	30. HUI 3
	31. HUI 2
	32. 19 or 20 or 21 or 22 or 23 or 24 or 25 or 26 or 27 or 28 or 29 or 30 or 31
	33. 18 and 32

* HTA = health technology assessment

5.4. EXPERT CONSULTATION

Key uncertainties remaining after the literature reviews centred on:

- What would typical primary care management of AKI involve?
- What would typical secondary care management of AKI involve?
- Would there be a utility decrement after NSAIDs had been withdrawn?

The model structure and key assumptions were discussed with the multi-disciplinary members (including GPs, pharmacists, and lay collaborators) of the study team and other experts in AKI/CKD.

5.5. MODEL STRUCTURE

Our review of economic evaluations related to AKI found that the state transition (usually Markov) model approach was the most common. The literature searches identified three Markov models which examined costs and outcomes associated with AKI (182-184) and a cost of illness study exploring the impact of AKI in England (185). The only model which included health states for both AKI and CKD was from an HTA report by Hall et al (184), however this model only included health states relating to AKI in people on a critical care unit. This was also the case in two of the other models identified (182, 183). The cost of illness study included only people who developed AKI during an inpatient hospital admission and did not include community-acquired AKI. An additional search was conducted to try and find literature relating to the treatment of AKI in primary care (see Table 1).

The health states in our final model were based on those used in the model by Hall et al (184), and also incorporate AKI that is manged in primary care. The cohort can experience AKI that is managed in primary or secondary care with or without the need for renal replacement therapy (RRT). After these acute states, the cohort can progress to more severe CKD states, including end-stage kidney disease (ESKD) and to the absorbing “dead” state from any of these states.

Key assumptions:

- Patients who experience secondary care management of AKI can progress to a more severe CKD state (ESKD), reflecting potential permanent damage caused by AKI.
- Patients who experience secondary care management of AKI with RRT can progress to both ESKD and ESKD+RRT, reflecting potential permanent damage caused by AKI requiring RRT.
- Once someone enters ESKD they can no longer experience an AKI.

Fig 5.1. Final model structure

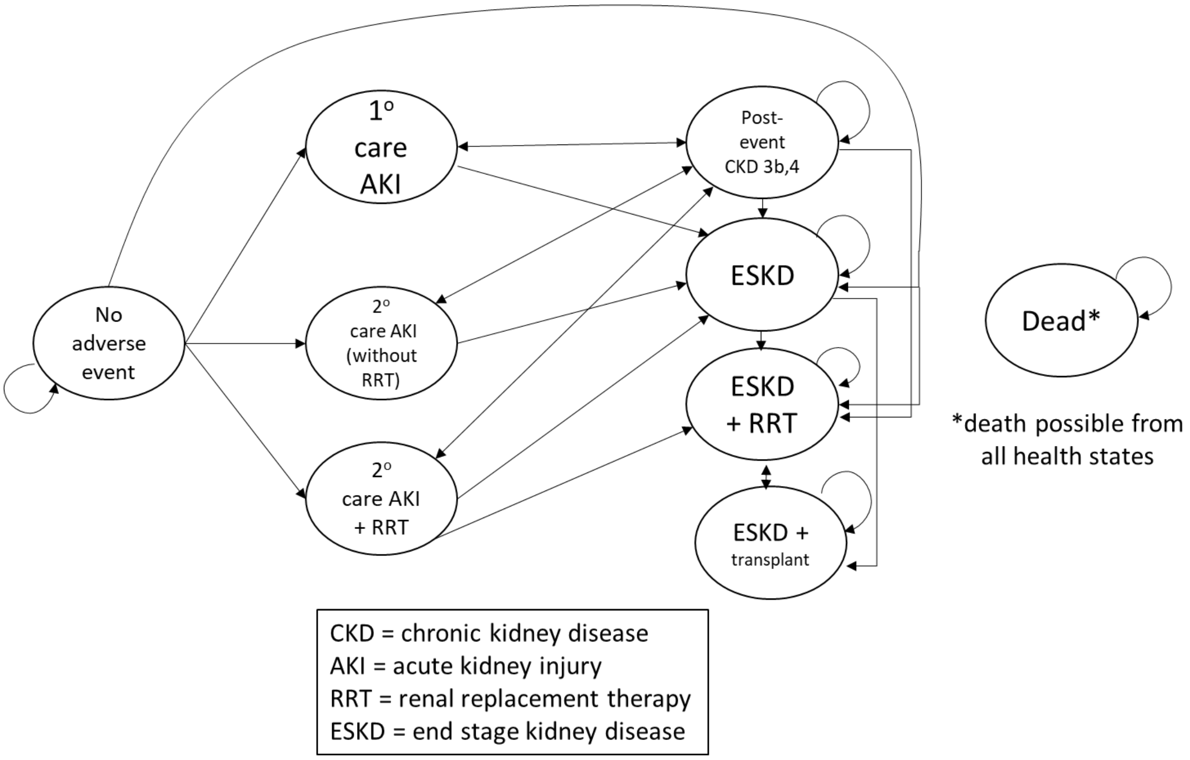

5.6. HEALTH STATES

The health states are summarised in Table 5.2.

Table 5.2. Health states

Health states	
No adverse event	This is the point of model entry.<br>People with an eGFR lower than 45 ml/min (i.e. CKD stage 3b-4). No assumptions are made regarding the underlying reason for NSAID prescription.
Primary care AKI	People experience AKI requiring treatment by a primary care physician
Secondary care AKI (no RRT)	People are admitted to hospital experiencing an AKI and do not require RRT
Secondary care AKI (with RRT)	People are admitted to hospital experiencing an AKI and do require RRT
Post-event CKD stage 3b-4	People with an eGFR lower than 45 ml/min (186)
EKRD	End stage renal disease (eGFR<15 ml/min; stage 5 CKD)) (186) not treated with RRT or transplant
EKRD + RRT	End stage renal disease (eGFR<15 ml/min) (186) treated with RRT
EKRD + transplant	Kidney transplant – successful transplant assumes normal kidney function; unsuccessful transplant assumes return to EKRD+RRT
Dead	There is a risk of death from all health states.

5.7. COHORT CHARACTERISTICS

This query specifies that the model cohort have an eGFR <45ml/min, which equates to stage 3b-4 CKD (stage 5 CKD is considered to be equivalent to ESRD, therefore these people were not included in the starting health state) (187). A CPRD study identified 165,942 adults in UK primary care databases with stage 3-5 CKD (188). Ninety-three percent of these people were aged over

60 years, but no further breakdown of the age of the sample was reported. Although not from the UK, a large Canadian study of routine data reported a mean age for people with stage 3b/4 CKD was 77 (189). An observational study exploring medical records for people from 3 regions in the UK reported the incidence of AKI by age group (190). The rate per 10,000 people was negligible in the 40-69 age group (90/10,000) whereas there was a large increase in those aged over 70 (500/10,000). Together, these studies suggest that the age group mostly affected by both AKI and CKD are those aged over 70, therefore the age at model entry was set at 70 years.

5.8. TRANSITION PROBABILITIES

The transition probabilities used in the model are described in this section and summarised in Table 6.1.

5.8.1. Probability of secondary care AKI without RRT

A large Canadian cohort study of 920,985 adults reported the rate per 1000 person years of hospital admissions for AKI not requiring RRT for sub-groups of people according to their eGFR (≥60 ml/min (no or mild CKD), 45-59.9 ml/min (stage 3a CKD), 30-44.9 ml/min (stage 3b CKD), and 15-29.9 ml/min (stage 4 CKD)). (189) The last two of these subgroups are equivalent to stage 3b/4 CKD (i.e. the model cohort) so a weighted mean rate across these two subgroups was calculated (38.6 [95%CI 37.0 to 40.3] per 1000 person years). The 3-month probability of secondary care AKI without RRT was derived from this mean rate.

5.8.2. Probability of secondary care AKI with RRT

James et al also reported the rate per 1000 person years of hospital admissions for AKI which *did* require RRT, again for sub-groups of people according to their eGFR (≥60 ml/min (no or mild CKD), 45-59.9 ml/min (stage 3a CKD), 30-44.9 ml/min (stage 3b CKD), and 15-29.9 ml/min (stage 4 CKD)) (189). A weighted mean rate across the stage 3b/4 eGFR subgroups was calculated (3.3 [95%CI 2.8 to 3.8] per 1000 person years). The 3-month probability of secondary care AKI with RRT was derived from this mean rate.

5.8.3. Age-specific probability of AKI

An unpublished analysis of Scottish health records data (from Bruce Guthrie) identified that in people with CKD (eGFR<60) the risk of AKI increases with age. The rate (95% CI) of AKI (per 1000 person years) in the *absence* of NSAIDs was as follows: 65-74 21.6 (20.4, 22.9); 75-84 31.0 (29.7, 32.2); 85+ 38.8 (36.6, 41.1). The probability of secondary care AKIs described above, came from an observational study where the mean age of the participants was in the 75-84 age group (189). Therefore, in the model it was assumed that the probabilities derived from that study reflected the likelihood of AKI in that group. The relative difference in the 3-month probability of AKI compared to the 75-84 group were used to estimate 3-month probabilities for the 70-74 group (relative difference 0.698) and the 85-89 group (relative difference 1.250) for the model.

5.8.4. Probability of primary care AKI

An analysis of health records covering a population of over 3m people in Wales identified cases of 22,723 cases of community-acquired AKI in 21,093 people, 6,534 of which were identified in primary care (191). Within this cohort of people with AKI that was identified in primary care, 2816 people had pre-existing CKD. Of the cases of AKI in people with CKD, 69.1% were not hospitalised (i.e. treated in primary care). The 3-month probability of having either type of secondary care AKI was assumed to be 30.9% of the cases of AKI. This was used to derive the probability of a primary care AKI (i.e. 69.1/30.9 times the probability of both of the secondary care AKIs).

5.8.5. Probability of CKD progression following secondary care AKI

The probability of progression to ESRD was estimated from an observational study, by Wonnacott et al, of 686 patients admitted for AKI to two UK general hospitals between 2011 and 2012 (192). There were 228 people in the study with pre-existing CKD, 42 (18%) were observed to have progression of their CKD following their AKI. It was not reported how many of the people with pre-existing CKD that were discharged on RRT, but of the whole sample of 686, only 9 (1%) were discharged on RRT. Following a secondary care AKI in the model, a probability of 0.01 (i.e. 1%) was used for the progression of CKD to ESKD requiring RRT. This 1% was deducted from the 18% in total with progression of CKD following AKI and a probability of 0.17 (i.e. 17%) was used for the progression of CKD to ESKD not requiring RRT.

5.8.6. *Probability of CKD progression following primary care AKI*

Following discussion with experts, the possibility of CKD progression following a primary care AKI was included in the model. No published data was identified regarding the likelihood of progression to ESRD following a primary care AKI. The consensus from the consulted experts was that it would be reasonable to assume that the likelihood of progression following a primary care AKI would be approximately half of that following a secondary care AKI. As described above, an observational study reported that 18% of people with CKD who were admitted to hospital with AKI, saw a progression of their CKD (192). Therefore, in the model it was assumed that 9% of people progressed to ESKD following a primary care AKI.

5.8.7. *Probability of progression of CKD (from no event or post-event) to subsequent ESKD health states*

Hall et al (184) derived transition probabilities relating to the background progression (natural history) of CKD from the SHARP study (193). This was a multinational study of individuals aged 40 years or over with moderate-to-severe CKD. Patients with prior myocardial infarction or coronary revascularization were excluded. The one-year rate of progressing from stage 3b/4 to ESRD *not* requiring RRT was 0.027 (SE 0.050) and from stage 3b/4 to ESKD requiring RRT (0.037 (SE 0.043)). The one-year probability of initiating RRT from ESKD was 0.149, SE 0.041. The 3-month probabilities of progression were derived from these values.

5.8.8. *Transition probabilities relating to kidney transplant*

Kent et al report the one-year rate of kidney transplantation (from ESKD no RRT: 0.053 (SE 0.069); from ESKD + RRT: 0.065 (SE 0.041)), which was derived from the SHARP study (193). As per the model by Hall et al, in the PROTECT model it was assumed that failure of a kidney transplant resulted in transition back to the ESKD+RRT health state. Data from the Renal Association was used in the Hall et al model to estimate the one-year probability of transplant failure (0.014 (SE 0.007) (184, 194). The risk of death following successful transplant was assumed to be equivalent to the general population probability of death which was derived from the Office for National Statistics (ONS) (41); this assumption was also used in the Hall et al model (184). The 3-month probabilities were derived from these values.

We note that the transplantation probabilities used here reflect people of all ages, whereas the population we are simulating has a relatively high mean age (70), and thus would be less likely to receive transplantation. The UK Renal Registry reports some age-stratified transplantation rates; however, these only go up to age 60–64, above which transplantation events are rare. We were unable to identify any data estimating probability of transplantation among the older people we are simulating; therefore, we performed a sensitivity analysis in which we set the rate to zero, to test whether this uncertainty has an important influence on model results.

5.8.9. *Probability of death (from non-AKI states)*

The probability of dying from the no event, post-event, and all of the ESKD health states were derived from the SHARP study, as reported by Kent et al (193). This was also the source study used by Hall et al (184). Kent et al report the one-year rate of death from CKD 3B/4 (0.030 (SE 0.047)), ESKD not requiring RRT (0.060 (SE 0.065)), and ESKD + RRT (0.074 (SE 0.038)). The 3-month probabilities for the 70-74 age group were derived from these values. The probability of death for the other age groups was estimated, based on the age-related increase in mortality in the general population according to ONS (41).

5.8.10. *Probability of death from AKI states*

The observational study by Wonnacott et al of people with secondary care AKI reported that the difference in post-AKI mortality was not significantly different between people with (versus without) pre-existing CKD (HR 0.92 (95% CI 0.76 to 1.13)) (192). This suggests that mortality data from studies of cohorts with or without CKD could be suitable for the model.

An analysis of population-level health records in Wales reported a 90-day mortality of 9.6% for AKI which was identified and treated in primary care (191); 38.5% of this group had pre-existing CKD. The same paper reported a 90-day mortality of 27.5% for community acquired AKI which was identified in secondary care; 34.6% of this group had pre-existing CKD. This figure combines cases of AKI which did or did not require RRT. Mortality is likely to be higher where RRT was required. A pair of papers was published by Kolhe et al using HES data for England estimated the in-hospital case-fatality following AKI for cases not requiring (195) and requiring RRT (196) – both papers included hospital and community acquired AKI therefore are not directly applicable to our model (which focuses on community acquired AKI). For the period 2008-2013, 27.1% of cases of AKI not requiring RRT died during the hospital admission (36.2% of these people had a history of renal disease) and 41.1% of cases where RRT was required died (39.0% of these people had a history of renal disease). This suggests that a relative risk of death for cases involving RRT versus those that do not is 0.441/0.271 = 1.517. Noting that the overall probability of death is a weighted sum of the (unknown) probabilities of death with and without RRT, weighted according to the probability that any case of AKI will require RRT, we can express the relationship using the following simultaneous equations:

$$\begin{cases} P_{death} = P_{death|RRT}P_{RRT} + P_{death|noRRT}(1 - P_{RRT}) \\ RR_{RRT-v-noRRT} = \dfrac{P_{death|RRT}}{P_{death|noRRT}} \end{cases}$$

Rearranging these gives

$$\begin{cases} P_{death|noRRT} = \dfrac{P_{death}}{(1 - P_{RRT}) + P_{RRT}RR_{RRT-v-noRRT}} \\ P_{death|RRT} = P_{death|noRRT}RR_{RRT-v-noRRT} \end{cases}$$

Data from James et al. (2011), as used in 4.3.1 and 4.3.2, give a probability of 0.078 that any secondary care AKI episode will require RRT. Plugging this number in gives our final probabilities that an episode of secondary care AKI will prove fatal:

$$\begin{cases} P_{death|noRRT} = \dfrac{0.275}{(1 - 0.078) + 0.078 \times 1.517} = 0.264 \\ P_{death|RRT} = 0.264 \times 1.517 = 0.401 \end{cases}$$

Kolhe et al. (2016) give adjusted odds ratios for the effect of age on probability of death following hospitalised AKI (1.42 [95%CI: 1.40 to 1.45] for people aged 65–74; 2.01 [95%CI: 1.98 to 2.04] for 75–84; 2.93 [95%CI: 2.89 to 2.97] for 85+; all compared with people younger than 65). We used these to stratify probability of death following secondary care AKI according to age. In the absence of similar figures for primary care AKI, we assumed the same relationship with age would obtain, and used the same odds ratios to adjust probabilities. The age-specific probability of death from each health state is summarised in Table x.

5.8.11. Detection and correction of HPE

We assume that an AKI will trigger a medication review during which the HPE will be identified. Once the HPE is identified it is rectified through stopping the prescription of NSAIDs. Both cohorts (HPE and non-HPE) have the same probability of an AKI once the HPE has been corrected; this approach was used in our earlier work and has been used in other Markov models in this area (30).

5.8.12. Impact of NSAIDs

In the model it is assumed that prescription of an NSAID to someone with stage 3b/4 CKD can increase the risk of an AKI. A 2017 meta-analysis reported a pooled odds ratio (OR) for the risk of AKI in the presence of NSAIDs in community-dwelling adults with pre-existing CKD. The OR was 1.63 (95% CI 1.22 to 2.19) (197). An unpublished analysis of Scottish health records data (from Bruce Guthrie) identified that in people with CKD (eGFR<60), the impact of NSAIDs on the risk of AKI increases with age. Adjusted incidence rate ratios (IRRs) (95% CI) for AKI in people exposed to NSAIDs in the previous 30 days were as follows: 70-74 1.70 (1.14, 2.54); 75-79 1.92 (1.39, 2.65); 80-84 2.21 (1.61, 3.03); 85-89 1.94 (1.35,2.77). The meta-analysis by Zhang et al (197) reported an odds ratio of 1.63 for the impact of NSAIDs on risk of AKI which is similar to the IRR for the 70-74 group in the Scottish data. Therefore, the relative differences in the values from the Scottish data, compared with the 70-74 group, were used to inflate the impact of NSAIDs on AKI risk from the meta-analysis for the 75-79 group (relative difference 1.13), 80-84 group (relative difference 1.30), and the 85-89 group (relative difference 1.14). The age-specific probabilities of AKI in the absence and presence of NSAIDs are summarised in Table 5.3.

Table 5.3. Age-specific 3-month probability of AKI events

	**Health state**	**Age group (years)**				**Source**
		70-74	75-79	80-84	85-89	
No NSAIDs	1° care AKI	0.0163 (0.0010)*	0.0233 (0.0011)*	0.0233 (0.0011)*	0.0291 (0.0017)*	Holmes (191); James (189)
	2° care AKI (no RRT)	0.0067 (0.0003)*	0.0096 (0.0002)*	0.0096 (0.0002)*	0.0120 (0.0005)*	James (189)
	2° care AKI (with RRT)	0.0006 (0.00005)*	0.0008 (0.0001)*	0.0008 (0.0001)*	0.0010 (0.0001)*	James (189)
Impact of NSAIDs (OR)		1.630 (0.241)*	1.841 (0.578)*	2.119 (0.671)*	1.860 (0.620)*	Zhang (197)
NSAIDs	1° care AKI	0.0262 (0.0042)*	0.0421 (0.0133)*	0.0481 (0.0147)*	0.0529 (0.0170)*	Holmes (191); James (189)
	2° care AKI (no RRT)	0.0109 (0.0017)*	0.0175 (0.0057)*	0.0201 (0.0063)*	0.0221 (0.0074)*	James (189)
	2° care AKI (with RRT)	0.0009 (0.0002)*	0.0015 (0.0005)*	0.0017 (0.0006)*	0.0019 (0.0007)*	James (189)

* SE calculated by simulation because probability reflects a combination of parameters and/or transformations (e.g. from annual rates to 3-monthly probabilities) as described above

Probabilities of AKI in the top part of the table were derived for the 75–84 age group. Age-specific multipliers from the Scottish medical records data were then applied to these probabilities to generate values for the other age groups (relative difference in risk of AKI vs.75-84 - 70-74: 0.698; 85-89: 1.250). The values in the top part of the table were transformed to odds, multiplied by the respective age-specific ORs in the middle of the table, with the result converted back to a probability scale to produce the values in the bottom part of the table.

5.8.12.1. Impact of NSAIDs on the progression of CKD

No published evidence was found on the impact of NSAIDs on the progression of CKD in the absence of an AKI, therefore in the model it is assumed that the likelihood of progressing from stage 3b/4 CKD to ESKD is the same in the HPE and non-HPE models. This was discussed with experts.

5.8.13. Summary of transition probabilities

The transition probabilities used in the model for both the HPE and non-HPE conditions are summarised in Table 6.1.

5.9. COSTS

In the other models, the costs for routine management of the underlying health condition was not included. However for this model, because the HPE (via the increased risk of AKI), is associated with a risk of accelerated disease progression (to health states associated with important management costs i.e. RRT and transplant), routine management costs were included in this model. The costs for the CKD and ESKD health states were derived from a UK-based trial which used hospital records to estimate annual secondary care costs for people with CKD (193). These costs were inflated and converted from a 1-year to a 3-month cycle length. An additional cost was included in the 'no event' health state in the HPE model for the NSAID prescription. In common with other models, we assumed this was naproxen 500mg four times daily. In the 'no HPE' arm, we accounted for the cost of an alternative painkiller, paracetamol 2×500mg three times daily.

The costs associated with a primary care AKI were estimated using a bottom-up approach through discussion with GPs as to how they would treat someone with stage 3b/4 CKD who presented with AKI. The GPs agreed that there would be a blood test to establish the occurrence of AKI, an appointment to try and resolve the AKI (e.g. removing the causal agent if something like NSAIDs), advice to the patient to take fluids and food, a second blood test to confirm the resolution of the AKI, and a follow-up appointment.

The costs for secondary care AKI were derived from a UK observational study of AKI admissions (192) and the UK Renal Registry AKI report which was published in 2020 (198). In the observational study, for people with community-acquired AKI, the paper reported a median length of stay (7 days; IQR 3-16) and proportion requiring ICU admission (4.7%). This paper also reported data on 334 people with hospital acquired AKI – it is important to note that the resource use is somewhat different for community-acquired and hospital-acquired AKI (i.e. community-acquired AKI: 4.7% had intensive care (ICU) admission, median length of stay 7 days (interquartile range (IQR) 3-16) vs. hospital-acquired AKI: 9.9% had ICU admission, median length of stay 15 days (IQR 7-26)). The Renal Registry AKI report included similar findings; in 2018 the median length of stay for community acquired AKI was 6 days (IQR 4-12) for 10,131 elective admissions and 8 days (IQR 4-16) for 142,897 emergency admissions. For hospital-acquired AKI the median length of stay was 14 days (IQR 8-26) for 15,594 elective admissions and 18 days (IQR 10-34) for 107,208 emergency admissions. Together this gives a clear message that it is

important that the cost parameters associated with AKI used in this model are for community-acquired AKI where possible, or costs will be over-estimated. The length of hospital stay assumed in the model was 7 days as this was the median from the UK observational study (192) and the mid-point of the medians for elective (6 days) and emergency (8 days) admissions from the Renal Registry report (198).

Typically, current practice in the NHS in England is for RRT to be carried out 3 times per week (186); therefore for people requiring this during their hospital admission for AKI, the cost of 3 sessions of RRT was added. Professor of Nephrology, Nick Selby, confirmed that where facilities were available for inpatient haemodialysis this would be carried out 3 times per week. Where facilities are not available, RRT would be administered continuously within an ICU setting. Costs associated with admission to ICU are included in the model (as described above). The cycle length in the model is 3 months (i.e. 12 weeks) therefore it was assumed that the hospital costs applied for 1 week and routine management costs applied for the other 11 weeks (i.e. 11/12 of the routine management cost). The post-admission management costs were calculated based on the proportion of people moving to the different post-event health states (rather than the health state they were in prior to admission). For example, if half of the people went from hospital to CKD and half to ESKD, then the cost for the health state would be the hospital admission cost plus 50% of the 11-week CKD routine cost and 50% of the 11-week ESKD routine cost. People who died after having been admitted to hospital, only incurred the costs associated with the hospital admission.

The costs included in the model are summarised in Table 6.2.

5.10. UTILITIES

The utility values used in the model are reported in Table 6.2. For each of the health states a decrement compared to the general population was calculated. Utility values (and SDs) for the general population were used from a publication by Ara and Brazier which derived them from the 3-level EQ-5D which was collected for over 40,000 people across four waves (2003, 2004, 2005 and 2006) of the Household Survey for England (HSE) (53). The HSE is a well-established annual survey conducted on randomly selected samples of the population living in private households in England. A systematic review of utility weights for different health states associated with CKD was published in 2020 (199). The review paper was screened to identify any additional potential source studies for this model.

5.10.1. Stage 3b/4 CKD

A UK based study (200) reported a mean utility value (derived from the EQ-5D-3L) for people with stage 4 CKD of 0.74 (95% CI 0.62-0.85). The mean age of the sample was 64 years – in the 60-64 age group the general population utility value (as reported by Ara and Brazier (53)) is 0.807 so we derived a 0.067 decrement for people with CKD. A study of people with CKD from France, Germany, Italy, Spain and the UK (201) reported mean utility values for people with stage 4 CKD of 0.737 (SD 0.28), with a mean age of 66.9 (i.e. in the age group 65-69). This also equates to a decrement of 0.067 compared with the general population value for the same age group reported by Ara and Brazier (53) (i.e. 0.804 – 0.737 = 0.067). This decrement was applied to the general population utility value from Ara and Brazier for all age groups spanned in the model and applied to the 'no event' and 'post-event'.

5.10.2. Primary care AKI

No literature was identified which reported utility values for people experiencing primary care AKI, therefore the decrement used in the model was the mid-point between the decrement for stage 3b/4 CKD (i.e. no event) and for secondary care AKI. It was assumed that people would

have the AKI level utility decrement for one week, followed by a decrement equivalent to ESKD (no RRT) for eleven weeks. This assumption was supported by experts consulted during model development.

5.10.3. Secondary care AKI (with or without RRT)

A UK-based study by Mistry et al asked 48 people who had been admitted to one of two hospitals due to AKI, to complete the EQ-5D during their admission (202). It was not possible to distinguish between people who received RRT during their admission and those who did not; therefore it was assumed that the values reported were for people not receiving RRT as a larger proportion of people who are admitted for an AKI do not receive RRT than receive it (189). The weighted mean utility value across both hospitals in the study was 0.368 (SD 0.35). The mean age of the sample was 66 years (i.e. in the 65-69 age group), therefore compared with the general population utility value for this age group (0.804 (53)), the utility decrement whilst in hospital was 0.436 (i.e. 0.804 – 0.368 = 0.436). To estimate the additional disutility associated with receiving RRT, the difference between the decrement applied to the health states in this model for ESKD with RRT (decrement = 0.127) and without RRT (decrement = 0.077) (see sections 4.5.4 and 4.5.5 below) was used (i.e. 0.127-0.077=0.05). For the secondary care AKI + RRT health state the additional decrement of 0.05 was added to the decrement for secondary care AKI derived from the paper by Mistry et al (i.e. 0.05+0.436 = 0.486). It was assumed that people would have the respective inpatient utility value for seven days (the average length of hospital admission due to community acquired AKI (192, 198)). It was also assumed that after discharge from hospital, utility values would not immediately return to pre-admission levels and so for the remainder of the model cycle (i.e. eleven weeks) utility would be equivalent to people with ESKD requiring RRT (i.e. lower utility than CKD stage 3b/4).

5.10.4. ESKD (no RRT)

A UK based study (200) reported a mean utility value (derived from the EQ-5D-3L) for people with stage 5 CKD (i.e. ESKD) of 0.73 (95% CI 0.62-1.0). The mean age of the sample was 64 years – in the 60-64 age group the general population utility value (as reported by Ara and Brazier (53)) is 0.807 so we derived a 0.077 decrement for people with ESKD. This decrement was applied to the general population utility value from Ara and Brazier for each age group included in the model.

5.10.5. ESKD + RRT

No literature from UK studies was identified but a study was identified from Singapore, which asked people with ESKD who were undergoing RRT to complete the EQ-5D-5L (203). The mean utility value reported was 0.68 (SD 0.36), with a mean age of 60.1 (i.e. in the age group 60-64). This equates to a decrement of 0.127 compared to the general population value for the same age group reported by Ara and Brazier (53) (i.e. 0.807 – 0.68 = 0.127). This decrement was applied to the general population utility value from Ara and Brazier for all age groups spanned in the model for this health state.

5.10.6. *ESKD + transplant*

A UK-based study administered the EQ-5D-5L to 512 kidney transplant recipients and 1704 people on the waiting list (i.e. ESKD + RRT) (204). They reported a mean utility of 0.773 for people on the waiting list and a utility gain of 0.054 for people who had had a transplant. They did not report a mean utility value for the transplant group therefore for the model this was calculated by adding the utility value reported for the waiting list condition (0.773) and the utility increment reported for the post-transplant condition (0.054) (i.e. 0.773 + 0.054 = 0.827). The median age of people in that study was in the 50-54 group, therefore this equates to a decrement of 0.007 compared to the general population value for the same age group reported by Ara and Brazier (53).

5.10.7. Disutility of correcting HPE

Consultation with clinical experts concluded that people with CKD would not typically experience a loss in utility when NSAIDs are withdrawn as alternative analgesics or anti-inflammatories (e.g. paracetamol or prednisolone) would be prescribed.

6. INPUT PARAMETER TABLES FOR EACH STATE TRANSITION MODEL

Table 6.1. Summary of probabilities for each state transition model

Transition probabilities (and effect of removing HPE	Estimate (SE/SD/95%CI), distribution and source
Query A&B: *Prescription of an oral NSAID, without co-prescription of an ulcer healing drug, to a patient aged ≥65 years (A) or with a history of peptic ulceration (B)*	
Annual rate of GI discomfort in HPE cohort over 1 year	0.648 (95% CI: 0.609, 0.688) Lognormal (40)
Annual rate of symptomatic ulcer in HPE cohort	0.0112 (95% CI: 0.0066, 0.0169) Lognormal (40)
Annual rate of serious GI event in HPE cohort	0.00343 (95% CI: 0.00332, 0.00356) Lognormal, NHS Medication Safety Dashboard (Q1 2015/16 - Q3 2019/20) (45)
3 month P[death from 'no adverse event', GI discomfort, or post-event] by age group	55-59: 0.0012; 60-64: 0.0018; 65-69: 0.0029; 70-74: 0.0046; 75+: 0.0079, Fixed(41)
SMR for death in 30 days after ulcer diagnosis	11.6 (95% CI: 9.6, 13.9) Lognormal(42)
SMR for death for remainder of cycle in which ulcer diagnosed	2.16 (95% CI: 2.05, 2.29) Lognormal(43)
3 month P[death from symptomatic ulcer] by age group	55-59: 0.0064; 60-64: 0.0099; 65-69: 0.0157; 70-74: 0.0244; 75+: 0.0429 (combination of p[death 'no adverse event'] and SMRs)
3 month Case-fatality probability from serious GI event	0.0810 (95% CI: 0.0785, 0.0835) Beta(46)
3 month P[death from serious GI event], by age group	55-59: 0.0328; 60-64: 0.0514; 65-69: 0.0810; 70-74: 0.1277; 75+: 0.2211 (combination of p[death 'no adverse event'] and case-fatality probablity)
Rate of recurring adverse events (1st cycle after event only)	0.158 (95% CI: 0.110, 0.205) Lognormal (18)
Effect of removing HPE	
RR GI discomfort in presence of PPI	0.34 (95% CI: 0.22, 0.54) Lognormal (21)
OR symptomatic ulcer in presence of PPI	0.28 (95% CI: 0.17, 0.46) Lognormal (22)
OR serious GI event in presence of PPI	0.35 (95% CI: 0.24, 0.52) Lognormal (22)
Query D – *Patients aged ≥18 years prescribed warfarin or OAC in combination with an oral NSAID*	
Annual rate GI discomfort (non-HPE)	0.0289 (95% CI: 0.0259, 0.0321) Lognormal (127) (205)
Annual rate symptomatic ulcer (non-HPE)	men: 0.0014 (95% CI: 0.0013, 0.0015)<br>women: 0.0011 (95% CI: 0.0010, 0.0012) Lognormal (132)
Annual rate serious GI event (non-HPE)	0.0069 (95% CI: 0.0053, 0.0090) Lognormal (145)
3 month P[recurring GI discomfort, symptomatic ulcer or serious GI event]	0.1009 (95% CI: 0.0878, 0.1149) Beta(137)

Transition probabilities (and effect of removing HPE	Estimate (SE/SD/95%CI), distribution and source
Annual rate stroke (non-HPE)	0.008 (95%CI: 0.006, 0.010) Lognormal (145)
HR for increase in risk of stroke per 10yr age	1.45 (95%CI: 1.26, 1.66) Lognormal(145)
3 month P[repeat stroke (in first 3 months after first event)] by age group	70-74: 0.0210; 75-79: 0.0210; 80-84: 0.0243; 85-89: 0.0243; Beta (146, 148)
3 month P[repeat stroke (more than 3 months after the first event)] by age group	70-74: 0.0067; 75-79: 0.0067; 80-84: 0.0097; 85-89: 0.0097; Beta (145, 150, 151)
3 month P[death in no adverse event, post-event states, after GI discomfort] by age group	70-74: 0.0115; 75-79: 0.0202; 80-84: 0.0355; 85-89: 0.0651. Lognormal (153) Age adjustment: ONS
3 month P[death after symptomatic ulcer] by age group	70-74: 0.0591; 75-79: 0.1019; 80-84: 0.1730; 85-89: 0.2982. Lognormal (153), SMR from (42, 43); Age adjustment: ONS
3 month P [death after serious GI event] by age group	70-74: 0.0636; 75-79: 0.1121; 80-84: 0.1981; 85-89: 0.3693. Beta, (141) Age adjustment: ONS
3 month P [death after stroke] by age group	70-74: 0.0564; 75-79: 0.0995; 80-84: 0.1759; 85-89: 0.3278. Beta, (141) Age adjustment: ONS
3 month P [death after surviving stroke] by age group	70-74: 0.0146; 75-79: 0.0256; 80-84: 0.0448; 85-89: 0.0818. Lognormal, (151, 153), Age adjustment: ONS
Effect of HPE	
OR GI discomfort	2.12 (95% CI: 1.73, 2.58) Lognormal (154)
OR symptomatic ulcer	1.70 (95% CI: 1.49, 1.94) Lognormal (155)
HR serious GI event	3.01 (95% CI: 1.63, 5.55) Lognormal (145)
HR stroke	2.71 (95% CI: 1.48, 4.96) Lognormal (145)
Query I – *Prescription of an oral NSAID to a patient with heart failure*	
3 month P[primary care heart failure event]	0.2900 Lognormal (173)
3 month P[secondary care heart failure event]	65-74: 0.1808; 75+: 0.2204 Lognormal (174)
3 month P[death from no event, post-event, or following primary care event], by age	65-74: 0.0217; 75-84: 0.0345; 85-94: 0.0608 Beta (175)
3 month P[death in cycle following secondary care event], by age	65-74: 0.1330; 75-84: 0.1706; 85-94: 0.2589 Beta, NICOR 2018-19 fo(177)r 30 day probability of death; NICOR 2012-13 for age stratification; Taylor (175) for remaining 2m of cycle
Effect of HPE	
OR primary care event in presence of NSAIDs	1.16 (95% CI: 1.13, 1.20) Lognormal (172)
OR secondary care event in presence of NSAIDs	1.16 (95% CI: 1.13, 1.20) Lognormal (172)
HR death in presence of NSAIDs	1.22 (95% CI: 1.07, 1.39) Lognormal (178)
Query K – *Prescription of an oral NSAID to a patient with chronic renal failure with an eGFR <45ml/min*	
3 month P[primary care AKI] by age	70-74: 0.0163; 75-79: 0.0233 ; 80-84: 0.0233; 85-89: 0.0291 (189, 191)

Transition probabilities (and effect of removing HPE	Estimate (SE/SD/95%CI), distribution and source
3 month P[secondary care AKI (no RRT)] by age	70-74: 0.0067; 75-79: 0.0096; 80-84: 0.0096; 85-89: 0.0120(189)
3 month P[secondary care AKI (+RRT)] by age	70-74: 0.0006; 75-79: 0.0008; 80-84: 0.0008; 85-89: 0.0010 (189)
3 month P [progression from no adverse event or post-event (CKD stage 3b/4)<br>to ESRD (CKD stage 5, no RRT)]<br>to ESRD (CKD stage 5, + RRT)]	0.0067 Lognormal (193)<br>0.0092 Lognormal (193)
3 month P [progression from ESRD (CKD stage 5, no RRT) to ESRD (CKD stage 5, + RRT)]<br>to ESRD (CKD stage 5, + transplant)]	0.0366 Lognormal (193)<br>0.0133 Lognormal (193)
3 month P[progression from ESRD (CKD stage 5, + RRT) to ESRD (CKD stage 5, + transplant)]	0.0162 Lognormal (193)
3 month P[progression from primary care AKI to ESRD (CKD stage 5, no RRT)]	0.0921 (192); expert opinion (half of analogous probability for secondary care AKI)
3 month P[progression from secondary care AKI (no RRT) to ESRD (CKD stage 5, no RRT)]	0.1842 (95% CI: 0.1367, 0.2369) Beta (192)
3 month P[progression from secondary care AKI (+ RRT) to ESRD (CKD stage 5, no RRT)]<br>to ESRD (CKD stage 5, + RRT)]	0.1711 (192)<br>0.0131 (95% CI:0.0060, 0.0229) Beta (192)
3 month P[transplant failure - return to ESRD (CKD stage 5, + RRT)]	0.0035 Beta (184, 194)
3 month P[death from no event or post-event], by age group	70-74: 0.0074; 75-79: 0.0131; 80-84: 0.0231; 85-89: 0.0431(193, 195)
3 month P[death after primary care AKI], by age group	70-74: 0.0960; 75-79: 0.1306; 80-84: 0.1306; 85-89: 0.1797 Beta (191, 195, 206)
3 month P[death after secondary care AKI (no RRT)], by age group	70-74: 0.2643; 75-79: 0.3371; 80-84: 0.3371; 85-89: 0.4257 Beta (41, 191, 206)
3 month P[death after secondary care AKI (+RRT)], by age group	70-74: 0.4009; 75-79: 0.4864; 80-84: 0.4864; 85-89: 0.5799 Lognormal(41, 195, 196)
3 month P[death from ESRD (no RRT)], by age group	70-74: 0.0149; 75-79: 0.0262; 80-84: 0.0461; 85-89: 0.0850 Lognormal (41, 193)
3 month P[death from ESRD (with RRT)], by age group	70-74:0.0183; 75-79: 0.0322; 80-84: 0.0566; 85-89: 0.1038 Lognormal (41, 193)
3 month P[death from ESRD (with transplant)], by age group	70-74: 0.0047; 75-79: 0.0083; 80-84: 0.0147; 85-89: 0.0275 Fixed (41)
Effect of HPE	
OR AKI in presence of NSAIDs, by age	70-74: 1.63; 75-79: 1.84; 80-84: 2.12; 85+: 1.86 Lognormal (197) Age multipliers from unpublished analysis of Scottish healthcare records (personal communication B. Guthrie)

AKI: acute kidney injury, CI: confidence interval; CKD: chronic kidney disease; ESRD: end-stage renal disease; GI: gastrointestinal; HPE: high-risk prescribing event; NSAID: non-steroidal anti-inflammatory drug; OAC: oral anticoagulant agent; OR: odds ratio; P: probability; PPI: proton

pump inhibitor; RR: relative risk; RRT: renal replacement therapy; SD: standard deviation; SE: standard error; SMR: standardised mortality ratio

Table 6.2. Summary of utilities and costs for each state transition model

Health state	Utility weight	Mean cost per patient (£, 2021 values)
Query A&B: *Prescription of an oral NSAID, without co-prescription of an ulcer healing drug, to a patient aged ≥65 years (A) or with a history of peptic ulceration (B)*		
No adverse event/post event	50-54: 0.8344 (95% CI: 0.824,0.843); 55-59: 0.8222(95% CI: 0.811,0.833); 60-64: 0.8072(95% CI: 0.793,0.821); 65-69: 0.8041(95% CI: 0.790,0.817); 70-74: 0.7790(95% CI: 0.766, 0.791); 75-79: 0.7533(95% CI: 0.739, 0.767); 80-84: 0.6985 (95% CI: 0.677, 0.719); 85-89: 0.6497(95% CI: 0.624, 0.675), beta (53)	HPE: 0<br>No HPE: 3.85 (SE 0.77*), gamma (7)
GI discomfort	Decrement: 0.079 (0.061, 0.096), beta, (165)	43.08 (SE 8.62*), gamma, (3, 7)
Symptomatic ulcer	Decrement: 0.102 (0.068, 0.136), beta, (53)	628.25 (SE 125.65*) gamma (3) (7)
Serious GI event	Decrement: 0.239 (0.093, 0.384), normal,(61)	3,284.28 (SE 259.08), gamma, (50)
Query D – *Patients aged ≥18 years prescribed warfarin or OAC in combination with an oral NSAID*		
No adverse event/post event	70-74: 0.7547; 75–79: 0.7298; 80-84: 0.6767; 85-89: 0.6294 (54) Beta, age adjustment (53)	HPE: 7.66 (SE 1.53*)<br>No HPE: 16.76 (SE £3.35*) gamma (7)
GI discomfort, symptomatic ulcer, serious GI event	Decrements as per query A/B	Costs as per query A/B
1st 3 months following stroke	Decrement: 0.22 (95% CI: 0.18, 0.26) Normal (207)	11,321.11 gamma (101, 163)
>3 months following stroke (post-stroke)	Decrement: 0.18 (95% CI: 0.13, 0.23) Normal (207)	250.12 gamma (101, 163)
Query I – *Prescription of an oral NSAID to a patient with heart failure*		
No adverse event (mild to moderate heart failure)/ post-event	0.7667 (95% CI: 0.7246, 0.8063), Beta (181)	HPE; cost of NSAID: 7.66 (SE: 1.53*) gamma, (208)<br>No HPE; cost of paracetamol: 16.76 (SE: 3.35*) gamma, (7)
Primary care event (one-level worsening of heart failure)	0.6817 (95%CI: 0.6401, 0.7218) Beta (181)	98.26 (SE: 19.65*) gamma, (7), Expert opinion
Secondary care event (two-level worsening of heart failure)	0.6017 (95%CI: 0.5605, 0.6422) Beta (181)	3,802.94 (SE: 760.59*) gamma (51, 209), Expert opinion
Query K – *Prescription of an oral NSAID to a patient with chronic renal failure with an eGFR <45ml/min*		

Health state	Utility weight	Mean cost per patient (£, 2021 values)
No adverse event/post-event state (CKD stage 3B-4)	70-74: 0.7118; 75-79: 0.6861; 80-84: 0.6313; 85-89: 0.5825 Beta (200) (53)	(HPE – plus NSAIDs): 794.67<br>(no HPE – plus paracetamol): 803.77 gamma, (193), (7)
3 months following primary care AKI	Decrement: 0.0906 (Expert opinion – half the disutility of secondary care AKI for the same duration)	873.04 Gamma, Expert opinion (193), (3, 8)
3 months following secondary care AKI (no RRT)	Decrement: 0.1509(202)	4,322.28 Gamma, (192, 193) (8)
3 months following secondary care AKI (+ RRT)	Decrement: 0.1547 (200, 202, 203)	4,800.29 Gamma, (192, 193) (8)
CKD stage 5 (no RRT) Utility decrement (compared to general population)	0.73 (95% CI: 0.62, 1.00) Beta(200) Decrement: 0.0770 (53, 200)	3,868.84 gamma (193)
CKD stage 5 (+ RRT) Utility decrement (compared to general population)	0.68 (95% CI: 0.6211, 0.7362) Beta(203) Decrement: 0.1272 (200, 202, 203)	6,126.76 gamma (193)
CKD stage 5 + transplant<br>Utility decrement (compared to general population) post-transplant	Decrement: 0.0074 (53, 204)	Transplant: 28,366.32 gamma (193)<br>Maintenance: 2,425.91 gamma (193)

* standard error assumed to be 20% of the mean, in the absence of empirical evidence of variability
AKI: acute kidney injury, CI: confidence interval; CKD: chronic kidney disease; ESKD: end-stage renal disease; GI: gastrointestinal; HPE: high-risk prescribing event; NSAID: non-steroidal anti-inflammatory drug; OAC: oral anticoagulant agent; OR: odds ratio; P: probability; PPI: proton pump inhibitor; RR: relative risk; RRT: renal replacement therapy; SD: standard deviation; SE: standard error; SMR: standardised mortality ratio

Appendix 3: One-way sensitivity analysis of parameters affecting cost and QALY estimates

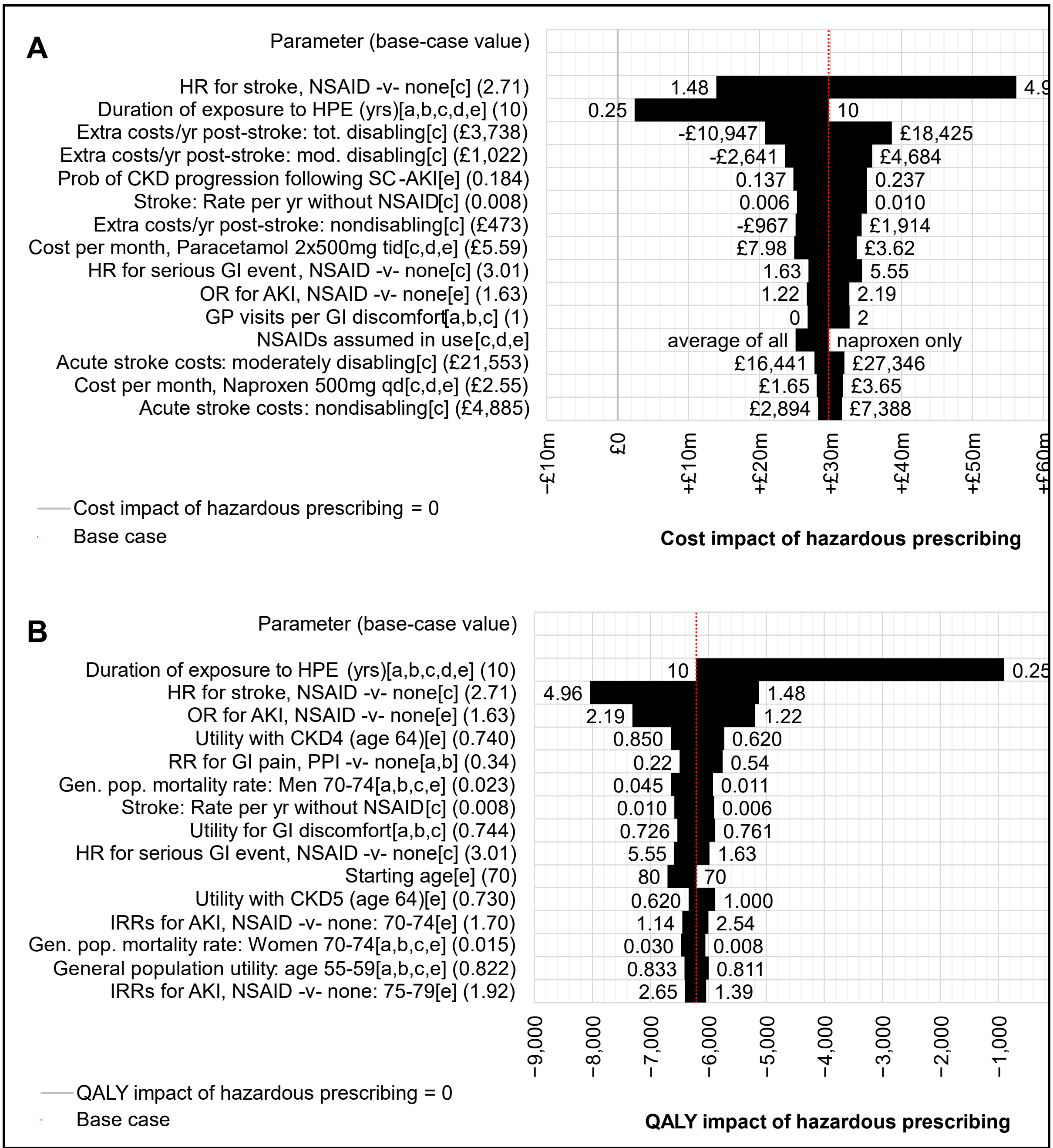

CKD = chronic kidney disease; Gen. pop. = general population; GI = gastrointestinal; HPE = hazardous prescribing event; HR = hazard ratio; IRR = incidence rate ratio; mod. = moderately; OR = odds ratio; PPI = proton pump inhibitor; QALY = quality-adjusted life-year; qd = once per day; RR = relative risk; SC-AKI = acute kidney injury requiring secondary care; tid = 3 times per day; tot. = totally

Each parameter was varied to a predefined upper and lower range, with all other parameters held at their base-case value, to demonstrate the impact that each individual parameter has on incremental costs and incremental QALYs. The 15 parameters with greatest impact on (A) costs and (B) QALYs are shown. The solid bar indicates the range of expected costs/QALYs the model generates when the parameter in question is varied between its lower and upper bound, centred around the base-case (vertical dotted red line). In most cases, we define the range using the upper and lower bounds of the parameter's 95% confidence interval; in cases where empirical uncertainty intervals are not available, we use the 95% confidence bounds of a gamma distribution with a coefficient of variation of 0.2. Note that it is not possible to infer results for parameter values at intermediate points in the range, as relationships between inputs and outputs are unlikely to be linear.

Each parameter name is annotated to indicate which HPE(s) the parameter in question influences:
[a] NSAID in older people without gastroprotection; [b] NSAID with previous peptic ulcer without gastroprotection; [c] NSAID with oral anticoagulant; [d] NSAID with heart failure; [e] NSAID with chronic kidney disease

Figure 6 Tornado diagrams of parameters affecting cost and QALY estimates

REFERENCES

1. PRIMIS Team. PINCER National Rollout Progress Report to NHS England and the AHSN Network. Nottingham; 2020.
2. National Institute for Health and Care Excellence. Guide to the methods of technology appraisal London, UK; 2013.
3. Curtis LB, A. Unit Costs of Health and Social Care 2020. Personal Social Services Research Unit, University of Kent, Canterbury.; 2021.
4. Vemer P, Corro Ramos I, van Voorn GAK, Al MJ, Feenstra TL. AdViSHE: A Validation-Assessment Tool of Health-Economic Models for Decision Makers and Model Users. PharmacoEconomics. 2016;34(4):349-61.
5. Husereau D, Drummond M, Augustovski F, de Bekker-Grob E, Briggs AH, Carswell C, et al. Consolidated Health Economic Evaluation Reporting Standards 2022 (CHEERS 2022) Statement: Updated Reporting Guidance for Health Economic Evaluations. Value in Health. 2022;25(1):3-9.
6. Ara R, Brazier JE. Using health state utility values from the general population to approximate baselines in decision analytic models when condition-specific data are not available. Value Health. 2011;14(4):539-45.
7. National Health Service. NHS Electronic Drug Tariff [Available from: http://www.drugtariff.nhsbsa.nhs.uk/.
8. NHS Digital. Reference Costs (Ref Costs): 2019-20 Reference Costs Collection. 2021.
9. EuroQol Group:EuroQol. A new facility for the measurement of health related quality of life. Health Policy. 1990;16:199-208.
10. van Reenen M, Janssen, B. . EQ-5D-5L User Guide: Basic information on how to use the EQ-5D-5L instrument. 2015.
11. Hernandez Alava M, Wailoo A, Grimm S, Pudney S, Gomes M, Sadique Z, et al. EQ-5D-5L versus EQ-5D-3L: The Impact on Cost Effectiveness in the United Kingdom. Value in Health. 2018;21(1):49-56.
12. NICE Decision Support Unit. Mapping EQ-5D-5L to EQ-5D-3L University of Sheffield2022 [Available from: https://nicedsu.sites.sheffield.ac.uk/methods-development/mapping-eq-5d-5l-to-3l.
13. National Institute for Health and Care Excellence. Position statement on use of the EQ-5D-5L valuation set for England (updated November 2018) 2018 [Available from: https://www.nice.org.uk/about/what-we-do/our-programmes/nice-guidance/technology-appraisal-guidance/eq-5d-5l].
14. Briggs A, Sculpher M, Claxton K. Decision Modelling for Health Economic Evaluation: Oxford University Press; 2006.
15. Lanas A, Chan FKL. Peptic ulcer disease. The Lancet. 2017;390(10094):613 - 24.
16. National Institute for Health and Clinical Excellence. Guidance on the use of cyclo-oxygenase (Cox) II selective inhibitors, celecoxib, rofecoxib, meloxicam and
etodolac for osteoarthritis and rheumatoid arthritis. London; 2001.
17. Ahmed A, Stanley AJ, Aging. Acute Upper Gastrointestinal Bleeding in the Elderly. Drugs & Aging. 2012;29(12):933-40.

18. Lau JY, Sung J, Hill C, Henderson C, Howden CW, Metz DC. Systematic review of the epidemiology of complicated peptic ulcer disease: incidence, recurrence, risk factors and mortality. Digestion. 2011;84(2):102-13.
19. National Institute for Health and Care Excellence. British National Formulary 2023. Available from: https://bnf.nice.org.uk.
20. National Institute of Health and Care Excellence. Non-steroidal anti-inflammatory drugs - KTT 113 National Institute of Health and Care Excellence; 2015.
21. Spiegel BM, Farid M, Dulai GS, Gralnek IM, Kanwal F. Comparing rates of dyspepsia with Coxibs vs NSAID+ PPI: a meta-analysis. The American journal of medicine. 2006;119(5):448. e27-. e36.
22. Scally B, Emberson JR, Spata E, Reith C, Davies K, Halls H, et al. Effects of gastroprotectant drugs for the prevention and treatment of peptic ulcer disease and its complications: a meta-analysis of randomised trials. The Lancet Gastroenterology & Hepatology. 2018;3(4):231-41.
23. Elliott RA, Putman KD, Franklin M, Annemans L, Verhaeghe N, Eden M, et al. Cost Effectiveness of a Pharmacist-Led Information Technology Intervention for Reducing Rates of Clinically Important Errors in Medicines Management in General Practices (PINCER). PharmacoEconomics. 2014;32(6):573-90.
24. Agah S, Akbari A, Heshmati J, Sepidarkish M, Morvaridzadeh M, Adibi P, et al. Systematic review with meta-analysis: Effects of probiotic supplementation on symptoms in functional dyspepsia. Journal of Functional Foods. 2020;68:103902.
25. Tian W, Jiang W, Yao J, Nicholson CJ, Li RH, Sigurslid HH, et al. Predictors of mortality in hospitalized COVID-19 patients: A systematic review and meta-analysis. Journal of Medical Virology. 2020.
26. NHS Specialist Pharmacy Service. Community Pharmacy NSAID Safety Audit 2018-19. 2019.
27. Brown T, Hooper L, Elliott R, Payne K, Webb R, Roberts C, et al. A comparison of the cost-effectiveness of five strategies for the prevention of non-steroidal anti-inflammatory drug-induced gastrointestinal toxicity: a systematic review with economic modelling. NIHR Health Technology Assessment programme: Executive Summaries: NIHR Journals Library; 2006.
28. Leontiadis G, Sreedharan A, Dorward S, Barton P, Delaney B, Howden C, et al. Systematic reviews of the clinical effectiveness and cost-effectiveness of proton pump inhibitors in acute upper gastrointestinal bleeding. NIHR Health Technology Assessment programme: Executive Summaries: NIHR Journals Library; 2007.
29. Chen Y, Jobanputra P, Barton P, Bryan S, Fry-Smith A, Harris G, et al. Cyclooxygenase-2 selective non-steroidal anti-inflammatory drugs (etodolac, meloxicam, celecoxib, rofecoxib, etoricoxib, valdecoxib and lumiracoxib) for osteoarthritis and rheumatoid arthritis: a systematic review and economic evaluation. NIHR Health Technology Assessment programme: Executive Summaries: NIHR Journals Library; 2008.
30. Chau SH, Sluiter RL, Kievit W, Wensing M, Teichert M, Hugtenburg JG. Cost effectiveness of gastroprotection with proton pump inhibitors in older low-dose acetylsalicylic acid users in the Netherlands. Drugs & aging. 2017;34(5):375-86.
31. Chau SH, Sluiter RL, Hugtenburg JG, Wensing M, Kievit W, Teichert M. Cost–Utility and Budget Impact Analysis for Stopping the Inappropriate Use of Proton Pump Inhibitors After Cessation of NSAID or Low-Dose Acetylsalicylic Acid Treatment. Drugs & aging. 2019:1-8.
32. De Groot N, Spiegel B, van Haalen H, de Wit N, Siersema P, van Oijen M. Gastroprotective strategies in chronic NSAID users: a cost-effectiveness analysis comparing single-tablet formulations with individual components. Value in health. 2013;16(5):769-77.
33. De Groot N, van Haalen H, Spiegel B, Laine L, Lanas A, Focks JJ, et al. Gastroprotection in low-dose aspirin users for primary and secondary prevention of ACS: results of a cost-effectiveness analysis including compliance. Cardiovascular drugs and therapy. 2013;27(4):341-57.

34. Saini S, Fendrick A, Scheiman J. Cost‐effectiveness analysis: cardiovascular benefits of proton pump inhibitor co‐therapy in patients using aspirin for secondary prevention. Alimentary pharmacology & therapeutics. 2011;34(2):243-51.
35. Takabayashi N, Murata K, Tanaka S, Kawakami K. Cost-effectiveness of proton pump inhibitor co-therapy in patients taking aspirin for secondary prevention of ischemic stroke. PharmacoEconomics. 2015;33(10):1091-100.
36. Bally M, Dendukuri N, Rich B, Nadeau L, Helin-Salmivaara A, Garbe E, et al. Risk of acute myocardial infarction with NSAIDs in real world use: bayesian meta-analysis of individual patient data. bmj. 2017;9:357.
37. Varas‐Lorenzo C, Riera‐Guardia N, Calingaert B, Castellsague J, Salvo F, Nicotra F, et al. Myocardial infarction and individual nonsteroidal anti‐inflammatory drugs meta‐analysis of observational studies. Pharmacoepidemiology and drug safety. 2013;22(6):559-70.
38. Office for National Statistics NRoS, ; Northern Ireland Statistics and Research Agency,. 2011 Census aggregate data. 2016.
39. Cai S, García Rodríguez L, Masso‐Gonzalez E, Hernandez‐Diaz S. Uncomplicated peptic ulcer in the UK: trends from 1997 to 2005. Alimentary pharmacology & therapeutics. 2009;30(10):1039-48.
40. Latimer N, Lord J, Grant RL, O'Mahony R, Dickson J, Conaghan PG. Cost effectiveness of COX 2 selective inhibitors and traditional NSAIDs alone or in combination with a proton pump inhibitor for people with osteoarthritis. Bmj. 2009;339:b2538.
41. Office for National Statistics. Deaths registered in England and Wales (2018). 2019.
42. Lassen A, Hallas J, de Muckadell OBS. Complicated and uncomplicated peptic ulcers in a Danish county 1993–2002: a population-based cohort study. American Journal of Gastroenterology. 2006;101(5):945-53.
43. Malmi H, Kautiainen H, Virta LJ, Farkkila MA. Increased short- and long-term mortality in 8146 hospitalised peptic ulcer patients. Aliment Pharmacol Ther. 2016;44(3):234-45.
44. Pettitt D, Goldstein J, McGuire A, Schwartz J, Burke T, Maniadakis N. Overview of the Arthritis Cost Consequence Evaluation System (ACCES) a pharmacoeconomic model for celecoxib. Rheumatology. 2000;39(suppl_2):33-42.
45. NHS Business Services Authority. Catalyst - public insight portal 2020 [
46. Crooks CJ, Card TR, West J. Defining upper gastrointestinal bleeding from linked primary and secondary care data and the effect on occurrence and 28 day mortality. BMC health services research. 2012;12(1):392.
47. Hearnshaw SA, Logan RF, Lowe D, Travis SP, Murphy MF, Palmer KR. Acute upper gastrointestinal bleeding in the UK: patient characteristics, diagnoses and outcomes in the 2007 UK audit. Gut. 2011;60(10):1327-35.
48. Straube S, Tramèr MR, Moore RA, Derry S, McQuay HJ. Mortality with upper gastrointestinal bleeding and perforation: effects of time and NSAID use. BMC gastroenterology. 2009;9(1):41.
49. Massó González E, García Rodríguez L. Proton pump inhibitors reduce the long‐term risk of recurrent upper gastrointestinal bleeding: an observational study. Alimentary pharmacology & therapeutics. 2008;28(5):629-37.
50. Campbell H, Stokes E, Bargo D, Logan R, Mora A, Hodge R, et al. Costs and quality of life associated with acute upper gastrointestinal bleeding in the UK: cohort analysis of patients in a cluster randomised trial. BMJ open. 2015;5(4):e007230.
51. Department of Health and Social Care. National Schedule of Reference Costs 2018-2019. 2019.
52. Elliott RA, Hooper L, Payne K, Brown TJ, Roberts C, Symmons D. Preventing non-steroidal anti-inflammatory drug-induced gastrointestinal toxicity: are older strategies more cost-effective in the general population? Rheumatology. 2006;45(5):606-13.

53. Ara R, Brazier JE. Using health state utility values from the general population to approximate baselines in decision analytic models when condition-specific data are not available. Value in Health. 2011;14(4):539-45.
54. Berg J, Lindgren P, Nieuwlaat R, Bouin O, Crijns H. Factors determining utility measured with the EQ-5D in patients with atrial fibrillation. Qual Life Res. 2010;19(3):381-90.
55. Lopez-Lopez JA, Sterne JAC, Thom HHZ, Higgins JPT, Hingorani AD, Okoli GN, et al. Oral anticoagulants for prevention of stroke in atrial fibrillation: systematic review, network meta-analysis, and cost effectiveness analysis. BMJ. 2017;359:j5058.
56. Thom HHZ, Hollingworth W, Sofat R, Wang Z, Fang W, Bodalia PN, et al. Directly Acting Oral Anticoagulants for the Prevention of Stroke in Atrial Fibrillation in England and Wales: Cost-Effectiveness Model and Value of Information Analysis. MDM Policy Pract. 2019;4(2):2381468319866828-.
57. Sterne JA, Bodalia PN, Bryden PA, Davies PA, Lopez-Lopez JA, Okoli GN, et al. Oral anticoagulants for primary prevention, treatment and secondary prevention of venous thromboembolic disease, and for prevention of stroke in atrial fibrillation: systematic review, network meta-analysis and cost-effectiveness analysis. Health Technol Assess. 2017;21(9):1-386.
58. Edwards S, Hamilton V, Nherera L, Trevor N, Barton S. Rivaroxaban for the prevention of stroke and systemic embolism in people with atrial fibrillation (TA 256). 2011.
59. Groeneveld PW, Lieu TA, Fendrick AM, Hurley LB, Ackerson LM, Levin TR, et al. Quality of life measurement clarifies the cost-effectiveness of Helicobacter pylori eradication in peptic ulcer disease and uninvestigated dyspepsia. The American journal of gastroenterology. 2001;96(2):338-47.
60. Spiegel BMR, Chiou C-F, Ofman JJ. Minimizing complications from nonsteroidal antiinflammatory drugs: Cost-effectiveness of competing strategies in varying risk groups. Arthritis Care & Research. 2005;53(2):185-97.
61. Doble B, Pufulete M, Harris JM, Johnson T, Lasserson D, Reeves BC, et al. Health-related quality of life impact of minor and major bleeding events during dual antiplatelet therapy: a systematic literature review and patient preference elicitation study. Health and quality of life outcomes. 2018;16(1):191.
62. Wang K, Li H, Kwong WJ, Antman EM, Ruff CT, Giugliano RP, et al. Impact of Spontaneous Extracranial Bleeding Events on Health State Utility in Patients with Atrial Fibrillation: Results from the ENGAGE AF‐TIMI 48 Trial. Journal of the American Heart Association. 2017;6(8):e006703.
63. Centre for Reviews and Dissemination. Search strategies 2019 [Available from: http://www.crd.york.ac.uk/crdweb/searchstrategies.asp#nhseedmedline].
64. National Institute for Health and Care Excellence. Developing NICE guidelines: the manual. 2014.
65. National Institute for Health and Care Excellence. NICE guideline 50-Cirrhosis in over 16s-Appendices A–H. 2016.
66. National Institute for Health and Care Excellence. NICE guideline 81-Glaucoma-Appendices A-T. 2017.
67. National Institute for Health and Clinical Excellence. NICE guideline 59-Low back pain and sciatica in over 16s: assessment and management-Appendix. 2016.
68. Elliott RA, Putman KD, Franklin M, Annemans L, Verhaeghe N, Eden M, et al. Cost Effectiveness of a Pharmacist-Led Information Technology Intervention for Reducing Rates of Clinically Important Errors in Medicines Management in General Practices (PINCER). Pharmacoeconomics. 2014:1-18.
69. Chau SH, Sluiter RL, Kievit W, Wensing M, Teichert M, Hugtenburg JG. Cost Effectiveness of Gastroprotection with Proton Pump Inhibitors in Older Low-Dose Acetylsalicylic Acid Users in the Netherlands. Drugs Aging. 2017;34(5):375-86.
70. Latimer N, Lord J, Grant RL, O'Mahony R, Dickson J, Conaghan PG. Cost effectiveness of COX 2 selective inhibitors and traditional NSAIDs alone or in combination with a proton pump inhibitor for people with osteoarthritis. Bmj. 2009;339:b2538.

71. Latimer N, Lord J, Grant RL, O'Mahony R, Dickson J, Conaghan PG. Value of information in the osteoarthritis setting: cost effectiveness of COX-2 selective inhibitors, traditional NSAIDs and proton pump inhibitors. Pharmacoeconomics. 2011;29(3):225-37.
72. de Groot NL, Spiegel BM, van Haalen HG, de Wit NJ, Siersema PD, van Oijen MG. Gastroprotective strategies in chronic NSAID users: a cost-effectiveness analysis comparing single-tablet formulations with individual components. Value in health : the journal of the International Society for Pharmacoeconomics and Outcomes Research. 2013;16(5):769-77.
73. de Groot NL, van Haalen HG, Spiegel BM, Laine L, Lanas A, Focks JJ, et al. Gastroprotection in low-dose aspirin users for primary and secondary prevention of ACS: results of a cost-effectiveness analysis including compliance. Cardiovasc Drugs Ther. 2013;27(4):341-57.
74. Wielage R, Bansal M, Wilson K, Klein R, Happich M. Cost-effectiveness of duloxetine in chronic low back pain: a Quebec societal perspective. Spine. 2013;38(11):936-46.
75. Wielage RC, Myers JA, Klein RW, Happich M. Cost-effectiveness analyses of osteoarthritis oral therapies: A systematic review. Applied Health Economics and Health Policy. 2013;11(6):593-618.
76. Brereton N, Pennington B, Ekelund M, Akehurst R. A cost-effectiveness analysis of celecoxib compared with diclofenac in the treatment of pain in osteoarthritis (OA) within the Swedish health system using an adaptation of the NICE OA model. J Med Econ. 2014;17(9):677-84.
77. Brereton N, Winn B, Akehurst R. The cost-effectiveness of celecoxib vs diclofenac in the treatment of osteoarthritis in the UK; an update to the NICE model using data from the CONDOR trial. J Med Econ. 2012;15(3):465-72.
78. Nasef SA, Shaaban AA, Mould-Quevedo J, Ismail TA. The cost-effectiveness of celecoxib versus non-steroidal anti-inflammatory drugs plus proton-pump inhibitors in the treatment of osteoarthritis in Saudi Arabia. Health economics review. 2015;5(1):53.
79. Chung S-J, Park H-J, Park M-C. Cost-effectiveness of Non-steroidal Anti-inflammatory Drugs Adjusting for Upper and Lower Gastrointestinal Toxicities in Rheumatoid Arthritis Patients. Journal of Rheumatic Diseases. 2017;24(1).
80. Moriarty F, Cahir C, Bennett K, Fahey T. Economic impact of potentially inappropriate prescribing and related adverse events in older people: a cost-utility analysis using Markov models. BMJ Open. 2019;9(1):e021832.
81. Elliott RA, Putman K, Franklin M, Verhaeghe N, Annemans L, Eden M, et al. Economic evaluation of a pharmacist-led IT-based intervention with simple feedback in reducing rates of clinically important errors in medicines management in general practices (PINCER). Birmingham, UK; 2013.
82. Varga Z, Sabzwari SRA, Vargova V. Cardiovascular Risk of Nonsteroidal Anti-Inflammatory Drugs: An Under-Recognized Public Health Issue. Cureus. 2017;9(4):e1144.
83. Lee SP, Sung IK, Kim JH, Lee SY, Park HS, Shim CS. Risk Factors for the Presence of Symptoms in Peptic Ulcer Disease. Clin Endosc. 2017;50(6):578-84.
84. Wielage RC, Bansal M, Andrews JS, Klein RW, Happich M. Cost-utility analysis of duloxetine in osteoarthritis: a US private payer perspective. Applied health economics and health policy. 2013;11(3):219-36.
85. Sorensen SV, Dewilde S, Singer DE, Goldhaber SZ, Monz BU, Plumb JM. Cost-effectiveness of warfarin: trial versus "real-world" stroke prevention in atrial fibrillation. Am Heart J. 2009;157(6):1064-73.
86. Spackman E, Burch J, Faria R, Corbacho B, Fox D, Woolacott N. Dabigatran etexilate for the prevention of stroke and systemic embolism in atrial fibrillation (TA 249). 2011.
87. Sorensen SV, Kansal AR, Connolly S, Peng S, Linnehan J, Bradley-Kennedy C, et al. Cost-effectiveness of dabigatran etexilate for the prevention of stroke and systemic embolism in atrial fibrillation: a Canadian payer perspective. Thromb Haemost. 2011;105(5):908-19.

88. González-Juanatey JR, Álvarez-Sabin J, Lobos JM, Martínez-Rubio A, Reverter JC, Oyagüez I, et al. Cost-effectiveness of Dabigatran for Stroke Prevention in Non-valvular Atrial Fibrillation in Spain. Revista Española de Cardiología (English Edition). 2012;65(10):901-10.
89. Kansal AR, Sharma M, Bradley-Kennedy C, Clemens A, Monz BU, Peng S, et al. Dabigatran versus rivaroxaban for the prevention of stroke and systemic embolism in atrial fibrillation in Canada. Comparative efficacy and cost-effectiveness. Thrombosis & Haemostasis. 2012;108(4):672-82.
90. Kansal AR, Sorensen SV, Gani R, Robinson P, Pan F, Plum JM, et al. Cost-effectiveness of dabigatran etexilate for the prevention of stroke and systemic embolism in UK patients with atrial fibrillation. Heart. 2012;98(7):573-8.
91. Janzic A, Kos M. Cost effectiveness of novel oral anticoagulants for stroke prevention in atrial fibrillation depending on the quality of warfarin anticoagulation control. Pharmacoeconomics. 2015;33(4):395-408.
92. Langkilde LK, Bergholdt Asmussen M, Overgaard M. Cost-effectiveness of dabigatran etexilate for stroke prevention in non-valvular atrial fibrillation. Applying RE-LY to clinical practice in Denmark. J Med Econ. 2012;15(4):695-703.
93. Zheng Y, Sorensen SV, Gonschior AK, Noack H, Heinrich-Nols J, Sunderland T, et al. Comparison of the cost-effectiveness of new oral anticoagulants for the prevention of stroke and systemic embolism in atrial fibrillation in a UK setting. Clinical Therapeutics. 2014;36(12):2015-28.e2.
94. Andrikopoulos GK, Fragoulakis V, Maniadakis N. Economic evaluation of dabigatran etexilate in the management of atrial fibrillation in Greece. Hellenic journal of cardiology : HJC = Hellenike kardiologike epitheorese. 2013;54(4):289-300.
95. Bergh M, Marais CA, Miller-Janson H, Salie F, Stander MP. Economic appraisal of dabigatran as first-line therapy for stroke prevention in atrial fibrillation. South African medical journal = Suid-Afrikaanse tydskrif vir geneeskunde. 2013;103(4):241-5.
96. Dorian P, Kongnakorn T, Phatak H, Rublee DA, Kuznik A, Lanitis T, et al. Cost-effectiveness of apixaban vs. current standard of care for stroke prevention in patients with atrial fibrillation. European Heart Journal. 2014;35(28):1897-906.
97. Giorgi MA, Caroli C, Giglio ND, Micone P, Aiello E, Vulcano C, et al. Estimation of the cost-effectiveness of apixaban versus vitamin K antagonists in the management of atrial fibrillation in Argentina. Health Economics Review. 2015;5 (1)(17).
98. Athanasakis K, Boubouchairopoulou N, Karampli E, Tarantilis F, Savvari P, Bilitou A, et al. Cost Effectiveness of Apixaban versus Warfarin or Aspirin for Stroke Prevention in Patients with Atrial Fibrillation: A Greek Perspective. American Journal of Cardiovascular Drugs. 2017;17(2):123-33.
99. Baron Esquivias G, Escolar Albaladejo G, Zamorano JL, Betegon Nicolas L, Canal Fontcuberta C, de Salas-Cansado M, et al. Cost-effectiveness Analysis Comparing Apixaban and Acenocoumarol in the Prevention of Stroke in Patients With Nonvalvular Atrial Fibrillation in Spain. Rev Esp Cardiol (Engl Ed). 2015;68(8):680-90.
100. Kongnakorn T, Lanitis T, Lieven A, Thijs V, Marbaix S. Cost effectiveness of apixaban versus aspirin for stroke prevention in patients with non-valvular atrial fibrillation in Belgium. Clinical Drug Investigation. 2014;34(10):709-21.
101. Lip GY, Kongnakorn T, Phatak H, Kuznik A, Lanitis T, Liu LZ, et al. Cost-effectiveness of apixaban versus other new oral anticoagulants for stroke prevention in atrial fibrillation. Clinical Therapeutics. 2014;36(2):192-210.e20.
102. Pradelli L, Calandriello M, Di Virgilio R, Bellone M, Tubaro M. Comparative pharmacoeconomic assessment of apixaban vs. standard of care for the prevention of stroke in Italian atrial fibrillation patients. Farmeconomia Health economics and therapeutic pathways. 2014;15(1S):15.

103. Lanitis T, Kongnakorn T, Jacobson L, De Geer A. Cost-effectiveness of apixaban versus warfarin and aspirin in Sweden for stroke prevention in patients with atrial fibrillation. Thromb Res. 2014;134(2):278-87.
104. Hallinen T, Soini EJ, Linna M, Saarni SI. Cost-effectiveness of apixaban and warfarin in the prevention of thromboembolic complications among atrial fibrillation patients. Springerplus. 2016;5(1):1354.
105. Gage BF, Cardinalli AB, Albers GW, Owens DK. Cost-effectiveness of warfarin and aspirin for prophylaxis of stroke in patients with nonvalvular atrial fibrillation. Jama. 1995;274(23):1839-45.
106. Lee S, Anglade MW, Meng J, Hagstrom K, Kluger J, Coleman CI. Cost-effectiveness of apixaban compared with aspirin for stroke prevention in atrial fibrillation among patients unsuitable for warfarin. Circ Cardiovasc Qual Outcomes. 2012;5(4):472-9.
107. Lee S, Anglade MW, Pham D, Pisacane R, Kluger J, Coleman CI. Cost-effectiveness of rivaroxaban compared to warfarin for stroke prevention in atrial fibrillation. Am J Cardiol. 2012;110(6):845-51.
108. Lee S, Mullin R, Blazawski J, Coleman CI. Cost-effectiveness of apixaban compared with warfarin for stroke prevention in atrial fibrillation. PLoS One. 2012;7(10):e47473.
109. Singh SM, Micieli A, Wijeysundera HC. Economic evaluation of percutaneous left atrial appendage occlusion, dabigatran, and warfarin for stroke prevention in patients with nonvalvular atrial fibrillation. Circulation. 2013;127(24):2414-23.
110. Ademi Z, Pasupathi K, Liew D. Cost-effectiveness of apixaban compared to warfarin in the management of atrial fibrillation in Australia. European Journal of Preventive Cardiology. 2015;22(3):344-53.
111. Nshimyumukiza L, Duplantie J, Gagnon M, Douville X, Fournier D, Lindsay C, et al. Dabigatran versus warfarin under standard or pharmacogenetic-guided management for the prevention of stroke and systemic thromboembolism in patients with atrial fibrillation: A cost/utility analysis using an analytic decision model. Thrombosis Journal. 2013;11 (1) (no pagination)(14).
112. Ademi Z, Pasupathi K, Liew D. Clinical and Cost Effectiveness of Apixaban Compared to Aspirin in Patients with Atrial Fibrillation: An Australian Perspective. Applied Health Economics and Health Policy. 2016:1-12.
113. Penner LS, Gavan SP, Ashcroft DM, Peek N, Elliott RA. Does coprescribing nonsteroidal anti-inflammatory drugs and oral anticoagulants increase the risk of major bleeding, stroke and systemic embolism? Br J Clin Pharmacol. 2022;88:4789-811.
114. Dalgaard F, Mulder H, Wojdyla DM, Lopes RD, Held C, Alexander JH, et al. Patients With Atrial Fibrillation Taking Nonsteroidal Anti-Inflammatory Drugs and Oral Anticoagulants in the ARISTOTLE Trial. Circulation. 2020;141(1):10-20.
115. Kent AP, Brueckmann M, Fraessdorf M, Connolly SJ, Yusuf S, Eikelboom JW, et al. Concomitant Oral Anticoagulant and Nonsteroidal Anti-Inflammatory Drug Therapy in Patients With Atrial Fibrillation. J Am Coll Cardiol. 2018;72(3):255-67.
116. Avery AJ, Rodgers S, Cantrill JA, Armstrong S, Cresswell K, Eden M, et al. A pharmacist-led information technology intervention for medication errors (PINCER): a multicentre, cluster randomised, controlled trial and cost-effectiveness analysis. The Lancet. 2012;379(9823):1310-9.
117. Edwards S, Hamilton V, Trevor N, Nherera L, Karnar C, Thurgar E. Apixaban (Eliquis®)for the prevention of stroke and systemic embolism in people with non-valvular atrial fibrillation (TA 275). 2012.
118. Halvorsen S, Storey RF, Rocca B, Sibbing D, Ten Berg J, Grove EL, et al. Management of antithrombotic therapy after bleeding in patients with coronary artery disease and/or atrial fibrillation: expert consensus paper of the European Society of Cardiology Working Group on Thrombosis. Eur Heart J. 2017;38(19):1455-62.
119. National Institute for Health and Care Excellence. Atrial fibrillation: management. 2014. Report No.: ISBN: 978-1-4731-0603-1.

120. National Collaborating Centre for Chronic Conditions. Osteoarthritis: National Clinical Guideline for Care and Management in Adults. UK; 2008.
121. Staerk L, Fosbol EL, Lamberts M, Bonde AN, Gadsboll K, Sindet-Pedersen C, et al. Resumption of oral anticoagulation following traumatic injury and risk of stroke and bleeding in patients with atrial fibrillation: A nationwide cohort study. European Heart Journal. 2018;39(19):1698-705.
122. Baker CL, Dhamane AD, Mardekian J, Dina O, Russ C, Rosenblatt L, et al. Comparison of Drug Switching and Discontinuation Rates in Patients with Nonvalvular Atrial Fibrillation Treated with Direct Oral Anticoagulants in the United States. Adv Ther. 2019;36(1):162-74.
123. Coleman CI, Tangirala M, Evers T. Treatment Persistence and Discontinuation with Rivaroxaban, Dabigatran, and Warfarin for Stroke Prevention in Patients with Non-Valvular Atrial Fibrillation in the United States. PLoS One. 2016;11(6):e0157769.
124. Groot AE, Vermeij JM, Westendorp WF, Nederkoorn PJ, van de Beek D, Coutinho JM. Continuation or Discontinuation of Anticoagulation in the Early Phase After Acute Ischemic Stroke. Stroke. 2018;49(7):1762-5.
125. Hellfritzsch M, Grove EL, Husted SE, Rasmussen L, Poulsen BK, Johnsen SP, et al. Clinical events preceding switching and discontinuation of oral anticoagulant treatment in patients with atrial fibrillation. Europace. 2017;19(7):1091-5.
126. Staerk L, Lip GYH, Olesen JB, Fosbøl EL, Pallisgaard JL, Bonde AN, et al. Stroke and recurrent haemorrhage associated with antithrombotic treatment after gastrointestinal bleeding in patients with atrial fibrillation: nationwide cohort study. BMJ. 2015;351.
127. Connolly SJ, Ezekowitz MD, Yusuf S, Eikelboom J, Oldgren J, Parekh A, et al. Dabigatran versus Warfarin in Patients with Atrial Fibrillation. New England Journal of Medicine. 2009;361(12):1139-51.
128. Ho KH, van Hove M, Leng G. Trends in anticoagulant prescribing: a review of local policies in English primary care. BMC health services research. 2020;20(1):279.
129. Staerk L, Gislason GH, Lip GY, Fosbol EL, Hansen ML, Lamberts M, et al. Risk of gastrointestinal adverse effects of dabigatran compared with warfarin among patients with atrial fibrillation: a nationwide cohort study. Europace. 2015;17(8):1215-22.
130. Lip GHY, Kongnakorn T, Phatak H, Kuznik A, Rublee D, Lanitis T, et al. Cost-effectiveness of apixaban against other novel oral anticoagulants (NOACs) for stroke prevention in atrial fibrillation patients. European Heart Journal. 2012;1):54.
131. Rosenstock S, Jørgensen T, Bonnevie O, Andersen L. Risk factors for peptic ulcer disease: a population based prospective cohort study comprising 2416 Danish adults. Gut. 2003;52(2):186-93.
132. Cai S, Garcia Rodriguez LA, Masso-Gonzalez EL, Hernandez-Diaz S. Uncomplicated peptic ulcer in the UK: trends from 1997 to 2005. Aliment Pharmacol Ther. 2009;30(10):1039-48.
133. Lin KJ, Garcia Rodriguez LA, Hernandez-Diaz S. Systematic review of peptic ulcer disease incidence rates: do studies without validation provide reliable estimates? Pharmacoepidemiol Drug Saf. 2011;20(7):718-28.
134. Yoon H, Kim SG, Jung HC, Song IS. High Recurrence Rate of Idiopathic Peptic Ulcers in Long-Term Follow-up. Gut Liver. 2013;7(2):175-81.
135. Malmi H, Kautiainen H, Virta LJ, Farkkila N, Koskenpato J, Farkkila MA. Incidence and complications of peptic ulcer disease requiring hospitalisation have markedly decreased in Finland. Aliment Pharmacol Ther. 2014;39(5):496-506.
136. Malmi H, Färkkilä MA, Virta LJ. Peptic ulcer disease - incidence, associated morbidity and mortality. Helsinki: University of Helsinki; 2018.
137. Tapaskar N, Pang A, Werner DA, Sengupta N. Resuming Anticoagulation Following Hospitalization for Gastrointestinal Bleeding Is Associated with Reduced Thromboembolic Events and Improved Mortality: Results from a Systematic Review and Meta-Analysis. Dig Dis Sci. 2020.
138. Lip GY, Kongnakorn T, Phatak H, Kuznik A, Lanitis T, Liu LZ, et al. Cost-effectiveness of apixaban versus other new oral anticoagulants for stroke prevention in atrial fibrillation. Clin Ther. 2014;36(2):192-210 e20.

139. Lanitis T, Cotte FE, Gaudin AF, Kachaner I, Kongnakorn T, Durand-Zaleski I. Stroke prevention in patients with atrial fibrillation in France: comparative cost-effectiveness of new oral anticoagulants (apixaban, dabigatran, and rivaroxaban), warfarin, and aspirin. J Med Econ. 2014;17(8):587-98.
140. Ariesen MJ, Claus SP, Rinkel GJ, Algra A. Risk factors for intracerebral hemorrhage in the general population: a systematic review. Stroke. 2003;34(8):2060-5.
141. Komen JJ, Forslund T, Mantel-Teeuwisse AK, Klungel OH, von Euler M, Braunschweig F, et al. Association of Preceding Antithrombotic Therapy in Atrial Fibrillation Patients With Ischemic Stroke, Intracranial Hemorrhage, or Gastrointestinal Bleed and Mortality. Eur Heart J Cardiovasc Pharmacother. 2019.
142. Penner LS, Gavan SP, Ashcroft DM, Peek N, Elliott RA. Does coprescribing nonsteroidal anti-inflammatory drugs and oral anticoagulants increase the risk of major bleeding, stroke and systemic embolism? British Journal of Clinical Pharmacology. 2022;88(11):4789-811.
143. Granger CB, Alexander JH, McMurray JJ, Lopes RD, Hylek EM, Hanna M, et al. Apixaban versus warfarin in patients with atrial fibrillation. N Engl J Med. 2011;365(11):981-92.
144. Lip GY, Clementy N, Pericart L, Banerjee A, Fauchier L. Stroke and major bleeding risk in elderly patients aged >/=75 years with atrial fibrillation: the Loire Valley atrial fibrillation project. Stroke. 2015;46(1):143-50.
145. van Walraven C, Hart RG, Connolly S, Austin PC, Mant J, Hobbs FD, et al. Effect of age on stroke prevention therapy in patients with atrial fibrillation: the atrial fibrillation investigators. Stroke. 2009;40(4):1410-6.
146. Hillen T, Coshall C, Tilling K, Rudd AG, McGovern R, Wolfe CDA. Cause of Stroke Recurrence Is Multifactorial. 2003;34(6):1457-63.
147. Hankey GJ, Jamrozik K, Broadhurst RJ, Forbes S, Burvill PW, Anderson CS, et al. Long-term risk of first recurrent stroke in the Perth Community Stroke Study. Stroke. 1998;29(12):2491-500.
148. Flach C, Muruet W, Wolfe CDA, Bhalla A, Douiri A. Risk and Secondary Prevention of Stroke Recurrence: A Population-Base Cohort Study. Stroke. 2020;51(8):2435-44.
149. Rasmussen LH, Larsen TB, Graungaard T, Skjøth F, Lip GYH. Primary and secondary prevention with new oral anticoagulant drugs for stroke prevention in atrial fibrillation: indirect comparison analysis. BMJ : British Medical Journal. 2012;345:e7097.
150. Hankey GJ, Patel MR, Stevens SR, Becker RC, Breithardt G, Carolei A, et al. Rivaroxaban compared with warfarin in patients with atrial fibrillation and previous stroke or transient ischaemic attack: a subgroup analysis of ROCKET AF. The Lancet Neurology. 2012;11(4):315-22.
151. Easton JD, Lopes RD, Bahit MC, Wojdyla DM, Granger CB, Wallentin L, et al. Apixaban compared with warfarin in patients with atrial fibrillation and previous stroke or transient ischaemic attack: a subgroup analysis of the ARISTOTLE trial. The Lancet Neurology. 2012;11(6):503-11.
152. Pamela J B, Joseph H, Matthew K, Thomas G B, Lee N, Judith M K, et al. Warfarin Use and Mortality, Stroke, and Bleeding Outcomes in a Cohort of Elderly Patients with non-Valvular Atrial Fibrillation. J Atr Fibrillation. 2019;12(1):2155-.
153. Gomez-Outes A, Lagunar-Ruiz J, Terleira-Fernandez AI, Calvo-Rojas G, Suarez-Gea ML, Vargas-Castrillon E. Causes of Death in Anticoagulated Patients With Atrial Fibrillation. J Am Coll Cardiol. 2016;68(23):2508-21.
154. Hippisley-Cox J, Coupland C, Logan R. Risk of adverse gastrointestinal outcomes in patients taking cyclo-oxygenase-2 inhibitors or conventional non-steroidal anti-inflammatory drugs: population based nested case-control analysis. Bmj. 2005;331(7528):1310-6.
155. Gonzalez-Perez A, Saez ME, Johansson S, Nagy P, Garcia Rodriguez LA. Risk factors associated with uncomplicated peptic ulcer and changes in medication use after diagnosis. PLoS One. 2014;9(7):e101768.
156. Blak BT, Thompson M, Dattani H, Bourke A. Generalisability of The Health Improvement Network (THIN) database: demographics, chronic disease prevalence and mortality rates. Informatics in primary care. 2011;19(4):251-5.

157. NHS Business Services Authority. English Prescribing Dataset - Jun 2020. 2020.
158. WHO Collaborating Centre for Drug Statistics Methodology. ATC/DD Index 2020: Norwegian Institute of Public Health; 2020 [Available from: https://www.whocc.no/atc_ddd_index/].
159. National Institute for Health and Care Excellence. NICE guideline 184-Gastro-oesophageal reflux disease and dyspepsia in adults: indyspepsia in adults: investigation and management. 2014.
160. Campbell HE, Stokes EA, Bargo D, Logan RF, Mora A, Hodge R, et al. Costs and quality of life associated with acute upper gastrointestinal bleeding in the UK: cohort analysis of patients in a cluster randomised trial. BMJ Open. 2015;5(4):e007230.
161. Vinogradova Y, Coupland C, Hill T, Hippisley-Cox J. Risks and benefits of direct oral anticoagulants versus warfarin in a real world setting: cohort study in primary care. Bmj. 2018:k2505.
162. Ali AN, Abdelhafiz A. Clinical and Economic Implications of AF Related Stroke. J Atr Fibrillation. 2016;8(5):1279-.
163. Luengo-Fernandez R, Yiin GS, Gray AM, Rothwell PM. Population-based study of acute- and long-term care costs after stroke in patients with AF. Int J Stroke. 2013;8(5):308-14.
164. Curtis L, Burns A. Personal Social Services Research Unit: Unit Costs of Health and Social Care 2020. 2021.
165. Delaney BC, Qume M, Moayyedi P, Logan RF, Ford AC, Elliott C, et al. Helicobacter pylori test and treat versus proton pump inhibitor in initial management of dyspepsia in primary care: multicentre randomised controlled trial (MRC-CUBE trial). Bmj. 2008;336(7645):651-4.
166. Tai M-LS, Norhatta N, Goh KJ, Moy FM, Sujarita R, Asraff AA, et al. The impact of dyspepsia on symptom severity and quality of life in adults with headache. PloS one. 2015;10(1).
167. Luengo-Fernandez R, Gray AM, Bull L, Welch S, Cuthbertson F, Rothwell PM, et al. Quality of life after TIA and stroke: ten-year results of the Oxford Vascular Study. Neurology. 2013;81(18):1588-95.
168. Di Tanna GL, Bychenkova A, O'Neill F, Wirtz HS, Miller P, Hartaigh BÓ, et al. Evaluating Cost-Effectiveness Models for Pharmacologic Interventions in Adults with Heart Failure: A Systematic Literature Review. PharmacoEconomics. 2019;37(3):359-89.
169. Levin R, Dolgin M, Fox C, Gorlin R. The Criteria Committee of the New York Heart Association: Nomenclature and Criteria for Diagnosis of Diseases of the Heart and Great Vessels1994. 344 p.
170. Goehler A, Geisler BP, Manne JM, Jahn B, Conrads-Frank A, Gazelle GS, et al. Decision-analytic models to simulate health outcomes and costs in heart failure. Pharmacoeconomics. 2011;29(9):753-69.
171. Rosen VM, Taylor DCA, Parekh H, Pandya A, Thompson D, Kuznik A, et al. Cost effectiveness of intensive lipid-lowering treatment for patients with congestive heart failure and coronary heart disease inthe US. PharmacoEconomics. 2010.
172. Girouard C, Grégoire JP, Poirier P, Moisan J. Effect of contraindicated drugs for heart failure on hospitalization among seniors with heart failure: A nested case-control study. Medicine (United States). 2017.
173. McMurray JJ, Trueman D, Hancock E, Cowie MR, Briggs A, Taylor M, et al. Cost-effectiveness of sacubitril/valsartan in the treatment of heart failure with reduced ejection fraction. Heart. 2018;104(12):1006-13.
174. Pufulete M, Maishman R, Dabner L, Mohiuddin S, Hollingworth W, Rogers CA, et al. Effectiveness and cost-effectiveness of serum B-type natriuretic peptide testing and monitoring in patients with heart failure in primary and secondary care: an evidence synthesis, cohort study and cost-effectiveness model. Health Technol Assess. 2017;21:1-150.
175. Taylor CJ, Ryan R, Nichols L, Gale N, Hobbs R, Marshall T. Survival following a diagnosis of heart failure in primary care. Family Practice. 2017.
176. NICOR: British Heart Foundation. National heart failure audit: April 2012-March 2013. 2013.

177. NICOR: British Heart Foundation. NATIONAL HEART FAILURE AUDIT (NHFA) 2020 SUMMARY REPORT (2018/19 DATA). 2020.
178. Gislason GH, Rasmussen JN, Abildstrom SZ, Schramm TK, Hansen ML, Fosbøl EL, et al. Increased Mortality and Cardiovascular Morbidity Associated With Use of Nonsteroidal Anti-inflammatory Drugs in Chronic Heart Failure. Archives of Internal Medicine. 2009;169(2):141-.
179. Open Prescribing. BNF Chapter 10.1.1: Non-Steroidal Anti-Inflammatory Drugs 2019 [Available from: https://openprescribing.net/bnf/100101/.
180. Griffiths A, Paracha N, Davies A, Branscombe N, Cowie MR, Sculpher M. Analyzing Health-Related Quality of Life Data to Estimate Parameters for Cost-Effectiveness Models: An Example Using Longitudinal EQ-5D Data from the SHIFT Randomized Controlled Trial. Advances in Therapy. 2017;34(3):753-64.
181. Kularatna S, Byrnes J, Chan YK, Ski CF, Carrington M, Thompson D, et al. Comparison of the EQ-5D-3L and the SF-6D (SF-12) contemporaneous utility scores in patients with cardiovascular disease. Quality of Life Research. 2017;26(12):3399-408.
182. Klarenbach S, Manns B, Pannu N, Clement FM, Wiebe N, Tonelli M, et al. Economic evaluation of continuous renal replacement therapy in acute renal failure. Int J Technol Assess Health Care. 2009;25(3):331-8.
183. Ethgen O, Schneider AG, Bagshaw SM, Bellomo R, Kellum JA. Economics of dialysis dependence following renal replacement therapy for critically ill acute kidney injury patients. Nephrology Dialysis Transplantation. 2015;30(1):54-61.
184. Hall P, Mitchell, ED, Smith, AF, Cairns, DA, Messenger, M, Hutchinson, M, Wright, J, Vinall-Collier, K, Corps, C, Hamilton, P, Meads, D, Lewington, A. The future of diagnostic tests of acute kidney injury in critical care: evidence synthesis, care pathway analysis and research prioritisation. Health Technology Assessment. 2018;22(32):308.
185. Kerr M, Bedford M, Matthews B, O'Donoghue D. The economic impact of acute kidney injury in England. Nephrology Dialysis Transplantation. 2014;29(7):1362-8.
186. National Health Service. Chronic Kidney Disease: National Health Service; 2019 [Available from: https://www.nhs.uk/conditions/kidney-disease/diagnosis/.
187. National Health Service. Chronic Kidney Disease: Diagnosis 2020 [Available from: https://www.nhs.uk/conditions/kidney-disease/diagnosis/.
188. Jameson K, Jick S, Hagberg K, Ambegaonkar B, Giles A, O'donoghue D. Prevalence and management of chronic kidney disease in primary care patients in the UK. International journal of clinical practice. 2014;68(9):1110-21.
189. James MT, Hemmelgarn BR, Wiebe N, Pannu N, Manns BJ, Klarenbach SW, et al. Glomerular filtration rate, proteinuria, and the incidence and consequences of acute kidney injury: a cohort study. The Lancet. 2010;376(9758):2096-103.
190. Sawhney S, Robinson HA, Van Der Veer SN, Hounkpatin HO, Scale TM, Chess JA, et al. Acute kidney injury in the UK: a replication cohort study of the variation across three regional populations. BMJ open. 2018;8(6):e019435.
191. Holmes J, Allen N, Roberts G, Geen J, Williams JD, Phillips AO, et al. Acute kidney injury electronic alerts in primary care ‐ findings from a large population cohort. QJM: An International Journal of Medicine. 2017;110(9):577-82.
192. Wonnacott A, Meran S, Amphlett B, Talabani B, Phillips A. Epidemiology and outcomes in community-acquired versus hospital-acquired AKI. Clinical Journal of the American Society of Nephrology. 2014;9(6):1007-14.
193. Kent S, Schlackow I, Lozano-Kühne J, Reith C, Emberson J, Haynes R, et al. What is the impact of chronic kidney disease stage and cardiovascular disease on the annual cost of hospital care in moderate-to-severe kidney disease? BMC nephrology. 2015;16(1):65.
194. The Renal Association. UK Renal Registry: 18th Annual Report of the Renal Association 2015. 2015.

195. Kolhe N, Muirhead A, Wilkes S, Fluck R, Taal M. The epidemiology of hospitalised acute kidney injury not requiring dialysis in England from 1998 to 2013: retrospective analysis of hospital episode statistics. International journal of clinical practice. 2016;70(4):330-9.
196. Kolhe NV, Muirhead AW, Wilkes SR, Fluck RJ, Taal MW. National trends in acute kidney injury requiring dialysis in England between 1998 and 2013. Kidney international. 2015;88(5):1161-9.
197. Zhang X, Donnan PT, Bell S, Guthrie B. Non-steroidal anti-inflammatory drug induced acute kidney injury in the community dwelling general population and people with chronic kidney disease: systematic review and meta-analysis. BMC nephrology. 2017;18(1):256.
198. The Renal Association. UK Renal Registry Acute kidney injury (AKI) in England A report on the nationwide collection of AKI warning test scores from 2018. 2020.
199. Cooper JT, Lloyd A, Sanchez JJG, Sörstadius E, Briggs A, McFarlane P. Health related quality of life utility weights for economic evaluation through different stages of chronic kidney disease: a systematic literature review. Health and quality of life outcomes. 2020;18(1):1-11.
200. Jesky MD, Dutton M, Dasgupta I, Yadav P, Ng KP, Fenton A, et al. Health-related quality of life impacts mortality but not progression to end-stage renal disease in pre-dialysis chronic kidney disease: a prospective observational study. PloS one. 2016;11(11):e0165675.
201. Eriksson D, Goldsmith D, Teitsson S, Jackson J, van Nooten F. Cross-sectional survey in CKD patients across Europe describing the association between quality of life and anaemia. BMC nephrology. 2016;17(1):97.
202. Mistry H, Abdelaziz TS, Thomas M. A prospective micro-costing pilot study of the health economic costs of acute kidney injury. Kidney international reports. 2018;3(6):1285-93.
203. Yang F, Lau T, Lee E, Vathsala A, Chia KS, Luo N. Comparison of the preference-based EQ-5D-5L and SF-6D in patients with end-stage renal disease (ESRD). The European Journal of Health Economics. 2015;16(9):1019-26.
204. Li B, Cairns JA, Draper H, Dudley C, Forsythe JL, Johnson RJ, et al. Estimating health-state utility values in kidney transplant recipients and waiting-list patients using the EQ-5D-5L. Value in Health. 2017;20(7):976-84.
205. Sobieraj DM, White CM, Alikhanov S, Winkler S, Mediouni M, Kluger J, et al. The impact of antiplatelet and anticoagulant therapies on gastrointestinal symptoms in patients with atrial fibrillation: a systematic review. Ann Pharmacother. 2012;46(9):1220-31.
206. Holmes J, Phillips D, Donovan K, Geen J, Williams JD, Phillips AO, et al. Acute kidney injury, age, and socioeconomic deprivation: evaluation of a national data set. Kidney international reports. 2019;4(6):824-32.
207. Luengo-Fernandez R, Gray AM, Bull L, Welch S, Cuthbertson F, Rothwell PM. Quality of life after TIA and stroke: Ten-year results of the oxford vascular study. Neurology. 2013.
208. NHS Prescription Services. NHS Electronic Drug Tariff 2019 [Available from: http://www.drugtariff.nhsbsa.nhs.uk/.
209. National Cardiac Audit Programme. National Heart Failure Audit 2016/17 Summary Report. London: National Institute of Cardiovascular Outcomes Research (NICOR); 2017.

Model validation

1. **Completed CHEERS checklist(5) (Completed CHEERS checklists are available for each model on request)**

Topic	No.	Item	Location where item is reported
**Title**			
**Title**	1	Identify the study as an economic evaluation and specify the interventions being compared.	Title, Page 1
**Abstract**			
**Abstract**	2	Provide a structured summary that highlights context, key methods, results, and alternative analyses.	Abstract, Page 1-2
**Introduction**			
**Background and objectives**	3	Give the context for the study, the study question, and its practical relevance for decision making in policy or practice.	Introduction, Page 3
**Methods**			
**Health economic analysis plan**	4	Indicate whether a health economic analysis plan was developed and where available.	Methods, Page 4
**Study population**	5	Describe characteristics of the study population (such as age range, demographics, socioeconomic, or clinical characteristics).	Appendix
**Setting and location**	6	Provide relevant contextual information that may influence findings.	Methods, Page 5
**Comparators**	7	Describe the interventions or strategies being compared and why chosen.	Methods, Page 5
**Perspective**	8	State the perspective(s) adopted by the study and why chosen.	Methods, Page 5
**Time horizon**	9	State the time horizon for the study and why appropriate.	Methods, Page 5
**Discount rate**	10	Report the discount rate(s) and reason chosen.	Methods, Page 5
**Selection of outcomes**	11	Describe what outcomes were used as the measure(s) of benefit(s) and harm(s).	Methods, Page 4
**Measurement of outcomes**	12	Describe how outcomes used to capture benefit(s) and harm(s) were measured.	Appendix
**Valuation of outcomes**	13	Describe the population and methods used to measure and value outcomes.	Appendix
**Measurement and valuation of resources and costs**	14	Describe how costs were valued.	Appendix

Topic	No.	Item	Location where item is reported
**Currency, price date, and conversion**	15	Report the dates of the estimated resource quantities and unit costs, plus the currency and year of conversion.	Methods, Page 5
**Rationale and description of model**	16	If modelling is used, describe in detail and why used. Report if the model is publicly available and where it can be accessed.	Methods, Page 4; Appendix
**Analytics and assumptions**	17	Describe any methods for analysing or statistically transforming data, any extrapolation methods, and approaches for validating any model used.	Appendix
**Characterising heterogeneity**	18	Describe any methods used for estimating how the results of the study vary for subgroups.	Methods , Last paragraph
**Characterising distributional effects**	19	Describe how impacts are distributed across different individuals or adjustments made to reflect priority populations.	Methods , Last paragraph
**Characterising uncertainty**	20	Describe methods to characterise any sources of uncertainty in the analysis.	Not applicable
**Approach to engagement with patients and others affected by the study**	21	Describe any approaches to engage patients or service recipients, the general public, communities, or stakeholders (such as clinicians or payers) in the design of the study.	Methods, Page 5
**Results**			
**Study parameters**	22	Report all analytic inputs (such as values, ranges, references) including uncertainty or distributional assumptions.	Appendix, Tables 6.1 and 6.2
**Summary of main results**	23	Report the mean values for the main categories of costs and outcomes of interest and summarise them in the most appropriate overall measure.	Table 2
**Effect of uncertainty**	24	Describe how uncertainty about analytic judgments, inputs, or projections affect findings. Report the effect of choice of discount rate and time horizon, if applicable.	Figure 1
**Effect of engagement with patients and others affected by the study**	25	Report on any difference patient/service recipient, general public, community, or stakeholder involvement made to the approach or findings of the study	Page 5
**Discussion**			
**Study findings, limitations, generalisability, and current knowledge**	26	Report key findings, limitations, ethical or equity considerations not captured, and how these could affect patients, policy, or practice.	Discussion, Pages 6-8
**Other relevant information**			
**Source of funding**	27	Describe how the study was funded and any role of the funder in the identification, design, conduct, and reporting of the analysis	Page 9

Topic	No.	Item	Location where item is reported
**Conflicts of interest**	28	Report authors conflicts of interest according to journal or International Committee of Medical Journal Editors requirements.	Page 9

2. Model error checks

Item	Error checks	Works (yes/no)?	Comments
Model structure/parameters			
1.	Is the model structure appropriate? Are all appropriate transitions included? Is 'dead' an absorbing state? Is it possible to die from all appropriate states?	Yes	This applies for models of all queries
2.	Check that the cohort numbers & the sum of all the health state transition probabilities add to 1 in all cycles / across the decision tree.	Yes	This applies for models of all queries
3.	Were half cycle corrections applied consistently for all transitions?	Yes	This applies for models of all queries. Half cycle correction implemented by adding full costs/qalys in the 1st cycle and zero costs/qalys in the last cycle
4.	Have sub-group specific parameters e.g. mortality based on age or disease severity etc been applied?	Yes	Age dependent utility tariffs and age dependent mortality rates were implemented in the model where appropriate. This applies for models of all queries.
5.	Check for unrealistic results (negative costs, more events than possible ...)	Yes	No unrealistic results were found in the models.
Extreme/alternative values			
6.	Set all utilities to 0. The total QALY gain should be 0.	Yes	This applies for all models
7.	Set all costs to 0. The total costs should be 0.	Yes	This applies for all models
8.	Change the time horizon of the model and check that the outputs/results change accordingly.	Yes	Increased costs & qalys when the time horizon increases, decreased costs & qalys when the time horizon decreases. This applies for all models
Discounting			

9.	Confirm that a discount rate of 3.5% has been applied for costs and QALYs	Yes	This applies for all models
10.	Set the discount rates to 0. The undiscounted costs and QALYs should equal the discounted costs and QALYs.	Yes	This applies for all models
External validity			
11.	Are the utilities in the model reasonable compared to patients in the general population of the same age? Check (table below, otherwise here):	Yes	The justification of utilities used in the models is described in the report
PSA			
12.	Are all parameters assigned an appropriate distribution for probabilistic sensitivity analyses?	Yes	This applies for all models
13.	Check PSA output mean costs, QALYS and ICER compared to the deterministic results. If there is a large discrepancy, is this due to the nature of the model, or errors in the PSA?	(cautious) Yes	This applies for all models In the QALYs results, in the model for query A more QALYs are generated than in the deterministic model.
